# Significant perspectives on various viral infections targeted antiviral drugs and vaccines including COVID-19 pandemicity

**DOI:** 10.1186/s43556-022-00078-z

**Published:** 2022-07-15

**Authors:** Gandarvakottai Senthilkumar Arumugam, Kannan Damodharan, Mukesh Doble, Sathiah Thennarasu

**Affiliations:** 1 Bioengineering and Drug Design Lab, Department of Biotechnology, Indian Institute of Technology Madras (IITM), Chennai, Tamilnadu 600036 India; 2 Department of Organic and Bioorganic Chemistry, CSIR- Central Leather Research Institute (CLRI), Chennai, Tamilnadu 600020 India; 3 Saveetha Dental College & Hospitals, Saveetha Institute of Medical & Technical Sciences, Chennai, Tamilnadu 600077 India

**Keywords:** COVID-19, Antiviral drugs, Viral targets, Vaccines, Drug delivery, Nanocarriers

## Abstract

A virus enters a living organism and recruits host metabolism to reproduce its own genome and proteins. The viral infections are intricate and cannot be completely removed through existing antiviral drugs. For example, the herpes, influenza, hepatitis and human immunodeficiency viruses are a few dreadful ones amongst them. Significant studies are needed to understand the viral entry and their growth in host cells to design effective antivirals. This review emphasizes the range of therapeutical antiviral drugs, inhibitors along with vaccines to fight against viral pathogens, especially for combating COVID-19. Moreover, we have provided the basic and in depth information about viral targets, drugs availability, their mechanisms of action, method of prevention of viral diseases and highlighted the significances of anticoagulants, convalescent plasma for COVID-19 treatment, scientific details of airborne transmission, characteristics of antiviral drug delivery using nanoparticles/carriers, nanoemulsions, nanogels, metal based nanoparticles, alike the future nanosystems through nanobubbles, nanofibers, nanodiamonds, nanotraps, nanorobots and eventually, the therapeutic applications of micro- and nanoparticulates, current status for clinical development against COVID-19 together with environmental implications of antivirals, gene therapy etc., which may be useful for repurposing and designing of novel antiviral drugs against various dreadful diseases, especially the SARS-CoV-2 and other associated variants.

**Supplementary Information:**

The online version contains supplementary material available at 10.1186/s43556-022-00078-z.

## Introduction

Antiviral agents inhibit the virus proliferation, and prevent the reproduction of the genome, hinder the access to host cells, diminish the viral protein synthesis, or viral cluster process. Basically, a virus is composed of DNA/RNA genome in protein shell called a capsid [1] and some viruses have an external membrane and the surrounding outer lipid layer, known as "*envelope*^*"*^. Researchers engaged in “rational drug design” focus on developing antivirals [2, 3] that endeavor to assault these at each stage during their life cycle. Several class of mushrooms, exhibit antiviral properties [4] with synergistic effects. The chemical constituents isolated from fruit extracts showed a wide-ranging potential antiviral properties, however, isolation of an antiviral component is a tedious process. Typically, the antiviral drugs are under the category of (i) antimicrobials including antibiotic / antibacterial, antifungal and antiparasitic or (ii) on the basis of monoclonal antibodies [5]. The major antiviral sources can be relatively harmless to the host and as a result, they can be utilized for the treatment of the infectious cells termed as *viricides*. However, they are not suitable for treatment or neutralize/annihilate either internal or external surface regions of the viruses. Some plants containing natural viricides include, *Eucalyptus globulus*, *Leptospermum laevigatum* [6], *Phytolacca Americana, Mirabilis jalapa*, *Dianthus caryophullus* etc. and they can prevent the spread of many plant viruses [7].

## Origin and classification of major human viruses

**Fig. 1:**
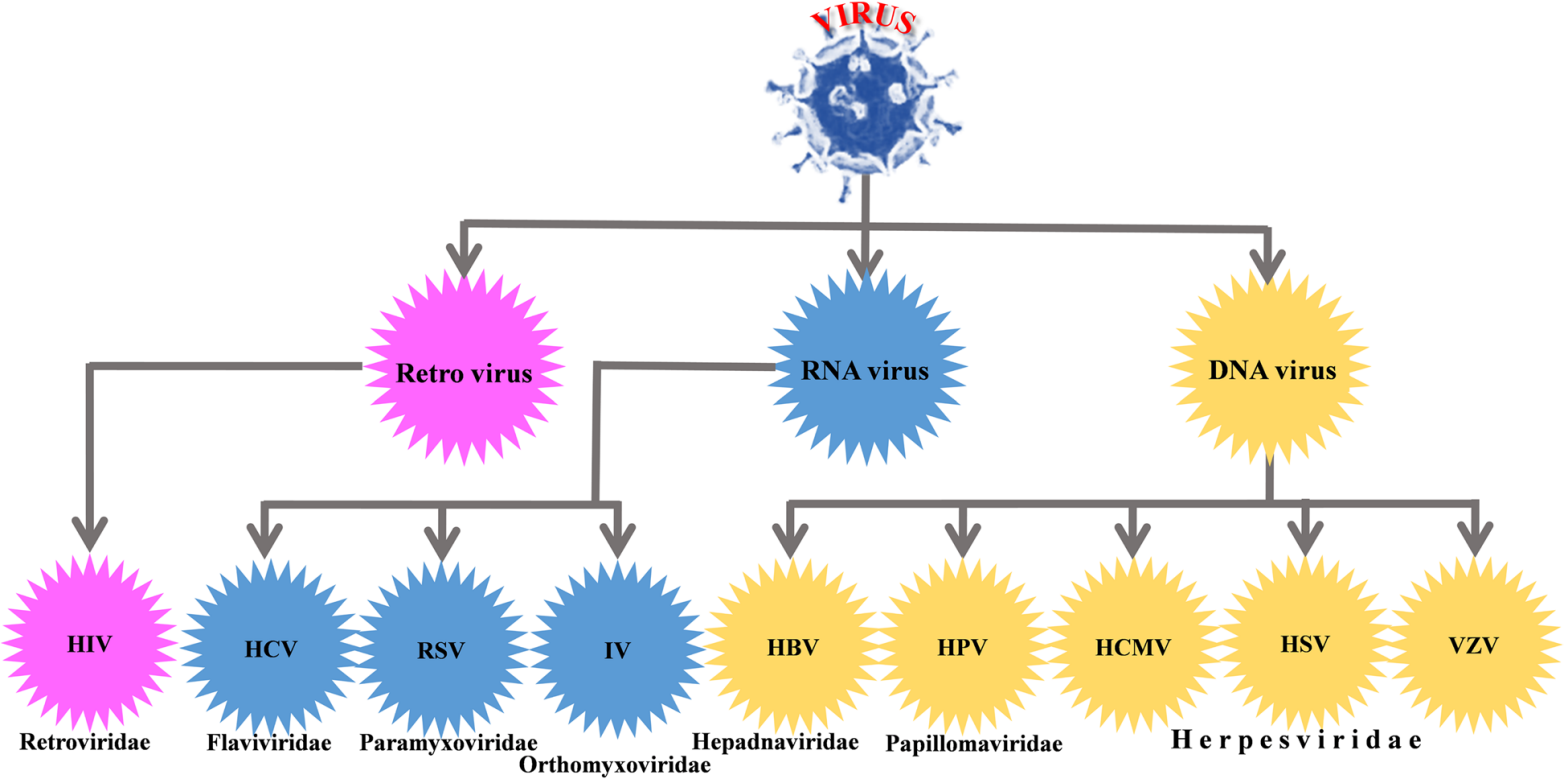
Classification of infective human viral diseases and their characteristic sub division and family are **a** Retrovirus (Human Immunodeficiency Virus (HIV), Family: Retroviridae); **b** RNA virus (Hepatitis C virus (HCV), Family: Flaviviridae), Respiratory Synctial virus (RSV), Family: Paramyxoviridae), Influenza virus (IV), Family: Orthomyxoviridae); **c** DNA virus (Hepatitis B virus (HBV), Family: Hepadnaviridae), Human Papilloma virus (HPV), Family: Papillomaviridae), (Human Cytomegalo virus (HCMV), Herpes simplex virus (HSV), Varicella-Zoster virus (VZV)-Family: Herpesviridae)

W. H. Prusoff reported the first antiviral drug, named idoxuridine (*5-iodo-20-deoxyuridine*). Its mode of action is inhibition of the viral replication by replacing itself by thymidine (pyrimidine deoxynucleoside) in viral DNA [8, 9]. This led to the discovery of many antiviral drugs. In 1963, idoxuridine drug was approved; Antiviral drugs are classified into different groups and are approved for the treatment of transmittable diseases, such as Human Immunodeficiency Virus (HIV), Hepatitis- B & C (HBV, HCV), Herpes-, Influenza-, Human Cytomegalo-, Varicella-Zoster-, Respiratory Syncytial-, Human Papilloma- viruses. Until recently, most of the researchers do not have even a fundamental knowledge about coronaviruses. However, various types of viruses, and the rationale of pneumonia infection in animals and humans have been documented about half a century back. The first human coronavirus was identified by virologist Dr. J. D. Almeida in 1964 at St. Thomas's Hospital in London and communicated in *Nature* journal in 1968 [10]. A variety of antiviral drugs are discovered recently for the treatment of nine major human viral infection including HIV, HCV, IV, RSV, HBV, HPV, HCMV, HSV, and VZV, wherein they fall into three categories: retro-, RNA- and DNA viruses, which are represented (Fig. 1). The more significant details are given in the supplementary information.

### Characteristics of common antiviral agent and in combinations

**Fig. 2:**
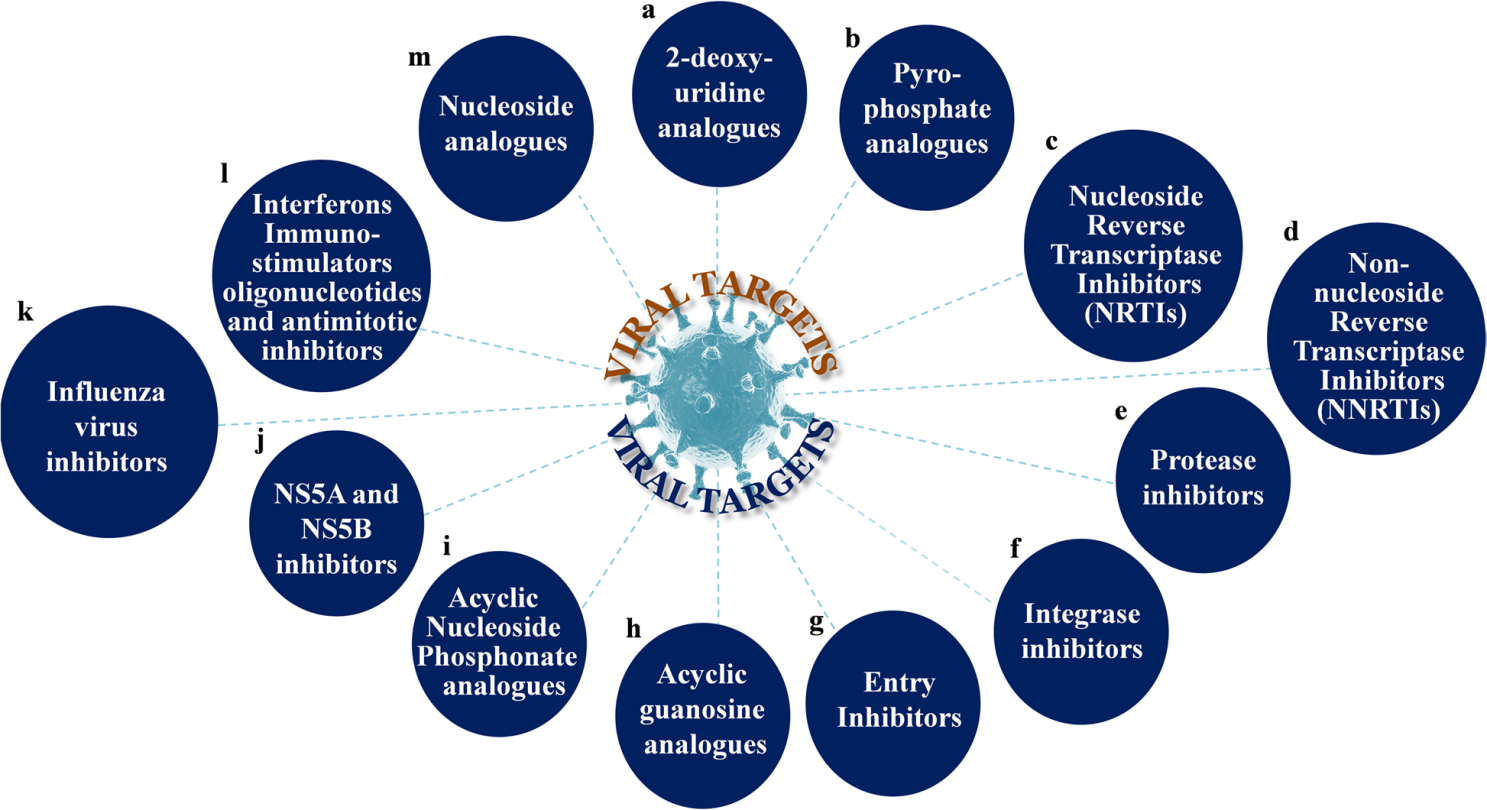
Illustrative lists of approved antiviral drugs (90) in different drug groups (13) against such nine human viruses including a 2-Deoxyuridine analogues: Idoxuridine, Vidarabine, Trifluridine, Brivudine, Entecavir, Telbivudine, Clevudine; **b** Pyrophosphate analogues: Foscarnet; **c** Nucleoside Reverse Transcriptase Inhibitors (NRTIs): Zidovudine, Didanosine, Zalcitabine, Stavudine, Lamivudine, Abacavir, Lamivudine + Zidovudine, Abacavir + Lamivudine + Zidovudine, Emtricitabine; **d** Non-Nucleoside Reverse Transcriptase Inhibitors (NNRTIS): Nevirapine, Delavirdine, Efavirenz, Etravirine, Etravirine, Rilpivirine; **e** Protease Inhibitors: Saquinavir, Ritonavir, Indinavir, Nelfinavir, Amprenavir, Lopinavir-Ritonavir, Atazanavir, Fosamprenevir, Tipranavir, Darunavir, Boceprevi; **f** Integrase Inhibitors: Raltegravir, Elvitegravir, Dolutegravir, Dolutegravir + abacavir + Lamivudine, Dolutegravir + lamivudine; **g** Entry Inhibitors:VZIG Human Varicella-Zoster immune Globulin, RSV-IGIV, Respiratory lsyscytia virus, immunue globulin Intravenous,Palivizumab, Docosanol, Enfuvirtide, Maraviroc, VariZig; **h** Acylic Guanosine Analogues: Acyclovir, Ganciclovir, Famciclovir, Valacyclovir, Penciclovir, Valganciclovir; **i** Acylic Nucleoside Phosphonate Analogues: Cidofovir, Adefovir dipivoxil, Tenofovir disoproxil fumarate, Tenofovir disoproxil fumarate + Emtricitabine, Tenofovir disoproxilfumarate + Emtricitabine + efavirenz, Tenofovirdisoproxil fumarate + Emtricitabine + elvitegravir, cobicistat, Tenofovir disoproxil fumarate + Emtricitabine + elvitegravir + cobicistat, Tenofovir disoproxil fumarate + cobicistat + Emtricitabine + elvitegravir, Tenofovir adafenamide + Emtricitabine, Rilpivirine + Tenofovir adafenamide + Emtricitabine, Tenofovir Alafenamide Fumarate + Emtricitabine, Grazoprevir; **j** NS5A and NS5B Inhibitors: Sofosbuvir + Ribavirin, Sofosbuvir + Ribavirin + pegIFNα, Daclatasvir + Asunaprevir, Daclatasvir Daclatasvir + Sofosbuvir, Daclatasvir + Simeprevir, Dasabuvir, Omibitasvir + dasabuvir + paritaprevir + ritonavir, Ledipasvir + Sofosbuvir, Omibitasvir + paritaprevir + Ritonavir, Daclatasivir + Sofobuvir, Elbasvir + Grazoprevir; **k** Influenza Virus Inhibitors: Amantadine, Ribavirin, Rimantadine, Zanamivir, Oseltamivir, Favipiravir, Laninamivir, Peramivir; **l** Interferons Immunostimulators, Oligonucleotides and Antimitotic Inhibitors: Pegylated interferons a, lfa 2b, Interferon alfacon-1, Imiquimod (INN), Pegylated interferons, alfa- 2b + ribavirin, Fomivirisen, Peginterferon, Alfa-2a, Sinecatechins; **m** Nucleoside Analogues: Vidarabine, Entecavir, Telbivudine, Telaprevir, Boceprevir, Simeprevir, Asunaprevir, Vaniprevir + Ribavirin + Peg-IFNα-2b, Paritaprevir, Asunaprevir, Darunuvir + Cobicistat, Atazanavir + cobicistat + Telaprevir

**Fig. 3:**
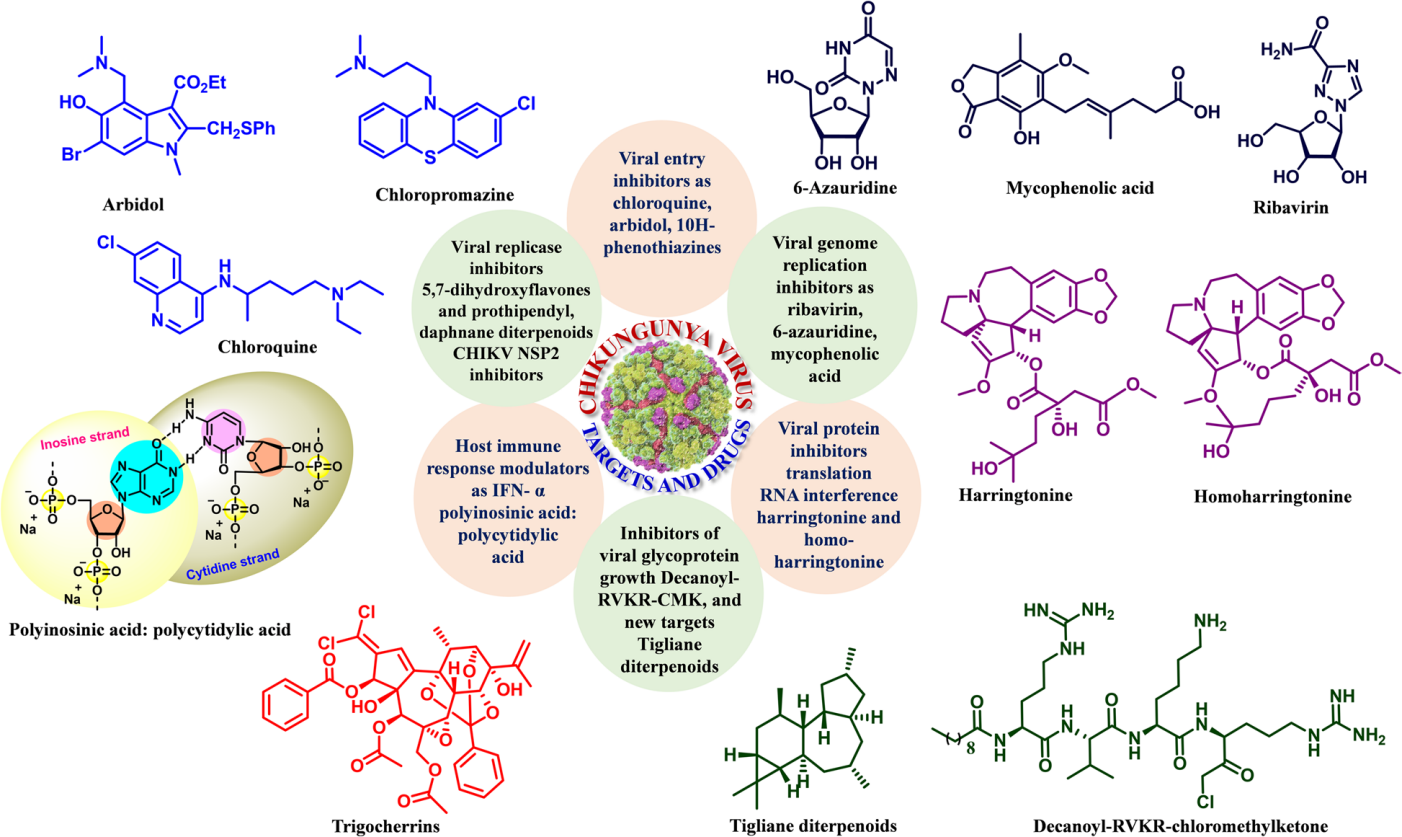
Representative active targets and drugs against chickungunya virus such as Viral entry inhibitors as chloroquine, arbidol, 10H-phenothiazines; Viral genome replication inhibitors as Ribavirin, 6-Azauridine, mycophenolic acid; Viral protein inhibitors translation RNA interference harringtonine and homo-harringtonine; Inhibitors of viral glycoprotein growth decanoyl-RVKR-CMK, and new targets Tigliane diterpenoids; Host immune response modulators as IFN- α polyinosinic acid: polycytidylic acid; Viral replicase inhibitors 5,7-dihydroxyflavones and prothipendyl, daphnanediterpenoids, CHIKV nsP2 inhibitors

**Fig. 4:**
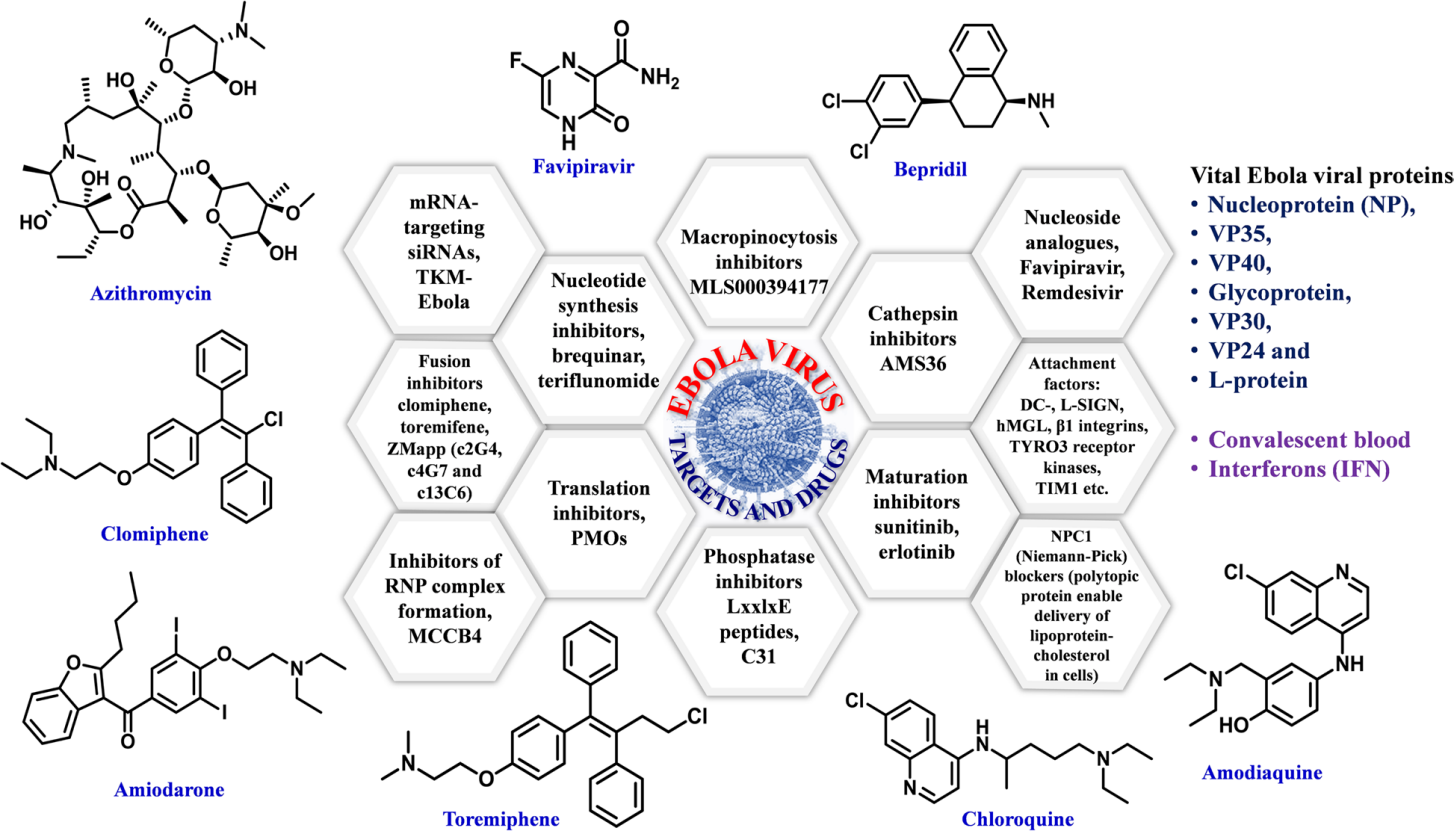
Characteristic targets and potent drugs against Ebola virus are illustrated as follows: **a** Targets as macropinocytosis inhibitors MLS000394177; cathepsin inhibitors AMS36; maturation inhibitors sunitinib, erlotinib; phosphatase inhibitors LxxlxE peptides, C31; Translation inhibitors PMOs; Nucleotide synthesis inhibitors, brequinar, teriflunomide; mRNA-targeting siRNAs, TKM-Ebola; Fusion inhibitors clomiphene, toremifene, ZMapp (c2G4, c4G7 and c13C6); Inhibitors of RNP complex formation MCCB4; Nucleoside analogues favipiravir, remdesivir; Attachment factors: DC-, L-SIGN, hMGL, β1 integrins, TYRO3 receptor kinases, TIM1 etc.; NPC1 (Niemann-Pick) blockers (polytopic protein enable delivery of lipoprotein- cholesterol in cells. **b** Drugs as Azithromycin, Amiodarone, Amodiaquine, Clomiphene, Bepridil, Favipiravir, Toremiphene, Chloroquine; Seven vital ebola viral proteins: Nucleoprotein (NP), VP35, VP40, Glycoprotein, VP30, VP24 and L-protein; Convalescent blood and Interferons (IFN)

**Fig. 5:**
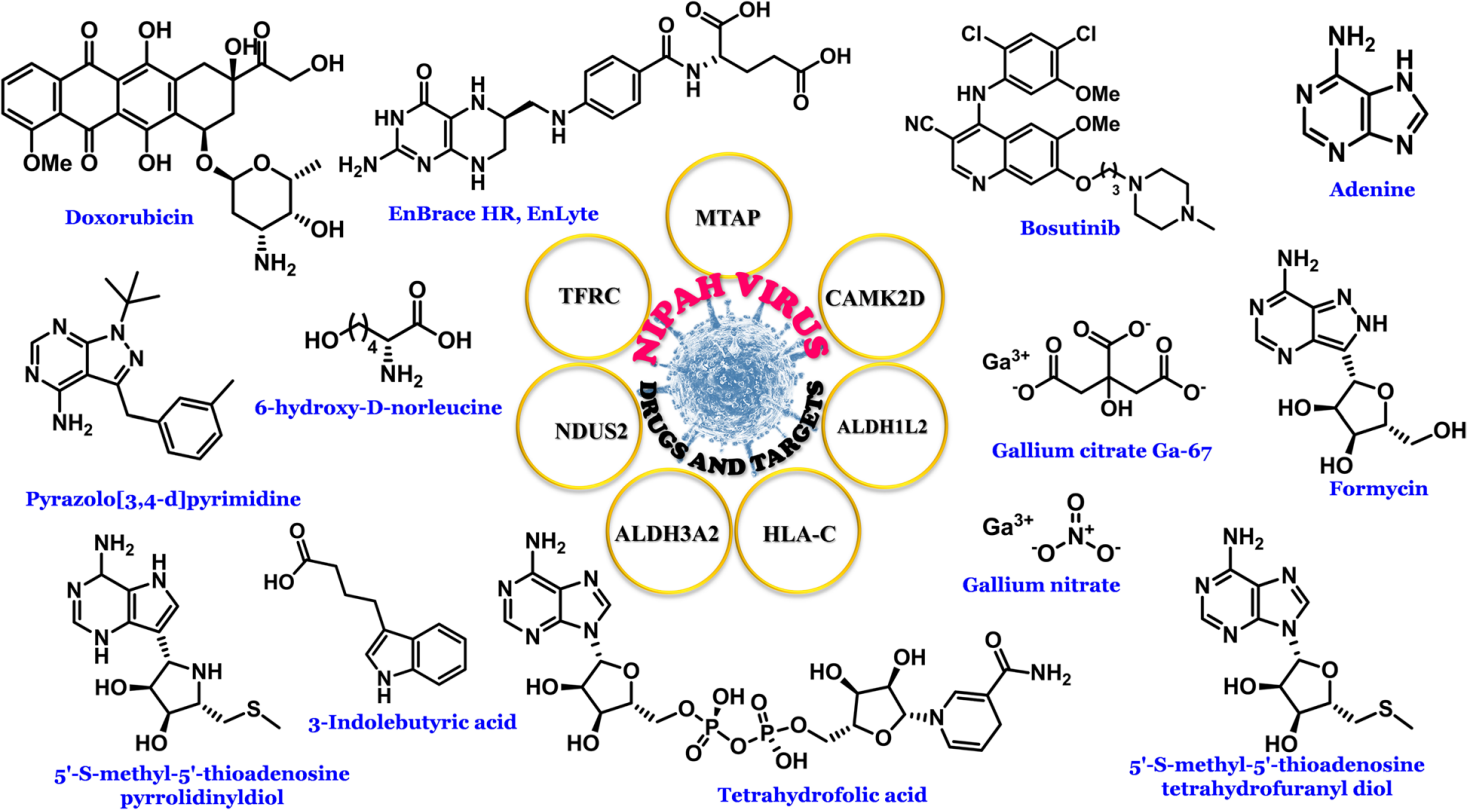
Illustrative representation of some active targets and drugs against nipah virus are as follows: **a** Targets are MTAP, CAMK2D, ALDH1L2, HLA-C, ALDH3A2, NDUS2, TFRC; **b** Drugs are as Doxorubicin, EnBrace HR (EnLyte), Bosutinib, Adenine, Pyrazolo[3,4-d]pyrimidine, 6-hydroxy-D-norleucine, Gallium citrate Ga-67, Formycin, 5'-S-methyl-5'-thioadenosine pyrrolidinyldiol, 3-Indolebutyric acid, Tetrahydrofolic acid, Gallium nitrate, 5'-S-methyl-5'-thioadenosine tetrahydrofuranyl diol

Despite a large number of compounds have been screened from chemical libraries for potential activity in cell culture against whole-virus reproduction, general public world over have still been affected by viral infection. Some of the treatment strategies seems to be a combination of two or more number of drugs. For example, the antiviral drug tenofovir disoproxil fumarate (TDF, Viread®) is a fumaric acid salt of bis-isopropoxycarbonyloxymethyl ester derivative of tenofovir. The salt form improves the solubility for the drug. This is used for chronic hepatitis B virus infected (HBV) patients [11]. Similarly the grouping of nucleotide or nucleoside reverse transcriptase inhibitors (N(t)RTIs) [12] with a tertiary representative through an additional anti-retroviral is suggested in favor of primary anti-retroviral therapy. In spite of exhibiting good antiviral efficacy, N(t)RTIs are useful for successful treatment, where the six nucleoside- and one nucleotide- reverse transcriptase inhibitor along with the two or more combinations may lead to be the permanent dosage respectively. Moreover, such kind of drug agents have diverse potent pharmacological and clinical characteristics. Whenever formulating a regimen, some of the properties such as the toxicological, drug load, dosage rate, potentiality of drug-drug bonding, pre-existing antiretroviral drug resistivity and co-morbidity should be measured. Such a strategy, namely to maximize virological efficiency is dangerous since it can lead to adverse clinical consequences. So, the comprehensive knowledge of the major human virus assists to devastate transmittable diseases that troubles millions of human inhabitants in the world. So we have taken effort to describe the available antiviral drugs for the past few decades. Initially we have listed the characteristic nine major human infectious viral targeting potential inhibitors (Supplementary Fig. 1) and consistent antiviral drugs [13] as shown (Fig. 2). Furthermore, we provided pictorial representation of various targets and drugs against the other dreadful viruses, for example, SARS/MERS-CoV (Supplementary Fig. 2), zika virus (Supplementary Fig. 3), dengue (Supplementary Fig. 4), chickungunya, ebola and nipah viruses, which are illustrated in (Figs. 3, 4 and 5) respectively [14–20]. Further, the descriptions of antiviral drug’s/inhibitor’s trade, generic names, therapeutic mechanism of action, diseases and approval year (Supplementary Table 1), alike, those under clinical trials for the current pandemic SARS-CoV-2 were illustrated.

## Significances and intrinsic studies of COVID-19

Generally, CoV (corona virus) (Fam. *coronaciridae*; sub fam. *orthocoronavirinae* order: Nidovirales, and realm: Riboviria) is a derivative of the Latin word, corona means ‘*crown’* or ‘*halo’*. It resembles a crown or a solar corona in the outer part of the virus, because of the peripheral region enclosed by a club-shaped protein spikes. The international committee on taxonomy of virus has proposed the name SARS-CoV-2 for the coronavirus provisionally called 2019-nCoV, or COVID-19 (Coronavirus Disease-19). Obviously, the deadly disease of COVID-19 is one of the respiratory diseases and this highly pathogenic coronavirus results in Acute Respiratory Distress Syndrome (ARDS), which cause enduring reduction of the lung function, arrhythmia, and leads to death. When compared to SARS or MERS, the SARS-CoV-2 emergent led to more vigorous spreading and generates intricacy to control [21, 22]. COVID-19, has spread rapidly across all the countries and the large number of conform cases prompted the WHO to declare it as a global pandemic on 11^th^ March, 2020.

**Fig. 6:**
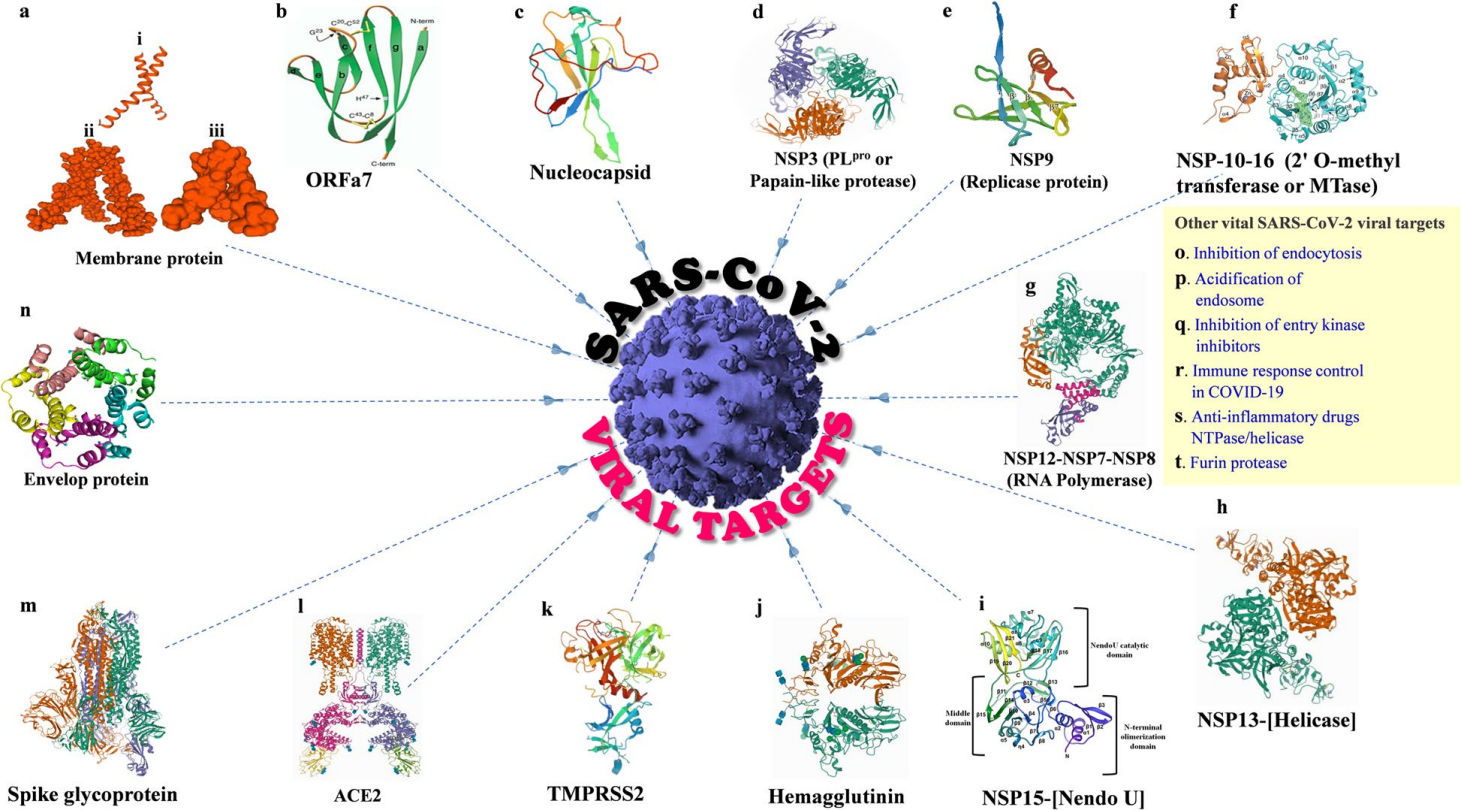
Typical viral targets against SARS-CoV-2 including **a** Membrane protein (i, ii, iii); **b** ORFa7; **c** Nucleocapsid; **d** (NSP9 (Replicase protein)); **e** NSP3 (PL.^pro^ or Papain-like protease); **f** NSP-10–16 (2' O-methyltransferase or MTase); **g** NSP12-NSP7-NSP8 (RNA Polymerase); **h** NSP13-[Helicase]; **i** NSP15-[Nendo U]; **j** Hemagglutinin; **k** TMPRSS2 (Transmembrane serine protease 2); **l** ACE2 (Angiotensin-Converting Enzyme 2); **m** Spike glycoprotein (highly immunogenic); **n** Envelop protein; In addition to that the other important SARS-CoV-2 viral targets are influenced as **o** Inhibition of endocytosis; **p** Acidification of endosome; **q** Inhibition of entry kinase inhibitors; **r** Immune response control in COVID-19; **s** Anti-inflammatory drugs NTPase/Helicase and **t** Furin protease

The current SARS-CoV-2 or COVID-19 is known as the seventh human coronavirus (HCoV) [23] after CoV-229E, NL63, OC43, HKU1, MERS, and SARS. Moreover this CoV is an enveloped positive-sense single standard RNA (ss-RNA), and the genome size ranges from 27–34 kilobase approximately, which is the largest among the known RNA virus/ The major features of CoV structural proteins are expressed as envelop (E), membrane (M), nucleocapsid (N) and the spike (S) (trimeric), proteins respectively. Number of viruses belonging to beta-CoVs contains hemagglutinin esterase (HE) glycoprotein. The RNA genome occurrence in CoV consists of seven genetic materials and are preserved in the arrangement of ORF1a, ORF1b, S, OEF3, E, M, N in 5' to 3' direction respectively. The two out of three equal parts of the RNA genome is enclosed by the ORF1a/b, generates the two viral replicase proteins such as polyproteins (PP1a and PP1ab). Sixteen developed nonstructural proteins (NSPs) occurred from auxiliary processing of polyproteins, and moreover these NSPs contributes in diverse viral functioning. This includes the development of the multifaceted assemblage of viral and cellular proteins like replicase-transcriptase complex. The residual genomic virus component encrypts the mRNA that creates the structural and other accessory proteins. Additionally, the main envelop-appended protein articulated by hemagglutinin esterase (HE) glycoprotein; also the RNA genomic sequential arrangement of CoV cluster surrounded in the nucleocapsid protein and enclosed with envelope. This spike surfaced glycoprotein is the ultimate component in the CoV particles and can detect angiotension-converting enzyme2 (ACE2), a transmembrane receptor protein in host cell of mammalian, this is exploited by the coronavirus to enter the host cell. Other important proteins are *papain-like protease* (PL^pro^) as encoded viral protease, *3C-like protease* (3CL^pro^) also labeled as Main protease (M.^pro^), Spike protein and remains, which make attractive viral targets for drug development (Fig. 6) [24–40]. Conversely, this CoV family seems to occurr in several species, amongst one such variety results in the infection to upper respiratory-, gastrointestinal tract region especially in mammals and birds,. In addition to SARS, MERS and COVID-19 can create morbidity and more threat for humans. [41, 42]

### S ymptoms, reactivity and prevention of COVID-19

This pandemic COVID-19 influenced sick people across the world in different ways. The majority of infected patients showed mild to moderate symptoms. In general, the viral disease led to the loss of smelling sense (Anosmia), dry cough, fatigue, body pain, nasal blocking, severe- fever etc. Sometime no symptoms were observed. Though it may travel up to eight meters from exhalation, it may remain in the air for hours, as stated by WHO and Centers for Disease Control (CDC). Some of the practices used to prevent the COVID-19 such as frequent hand washing, regular cleaning of touched surface, maintain social distancing, follow rules while lockdowns, quarantines, and moreover intensive control measures, travel restrictions etc., have useful to minimize the spread of COVID-19 [43]. Wearing gloves, gowns, personal protective equipment, specifically surgical face masks efficiently defend against respiratory droplets [44].

### COVID-19 pandemic rolling updates

Since 13 March 2022 worldwide there are at least 455,565,230 (100%) cumulative cases (%) who have been diagnosed and 6,039,440 (100%) cumulative deaths (%) recorded due to COVID-19. Among the affected continents/countries, Europe is in leading position of 187,814,829 (41%) cases, whilst, USA has 148,915,745 (33%) cases and the highest death toll 2,663,786 (44%), followed by countries in Asia, Europe and S. America [45]. The number of deaths due to this virus when compared to their population is highest in USA, followed by several European countries.

### Burden and challenges of emerging and re-emerging diseases amid the COVID-19 pandemic

**Fig. 7:**
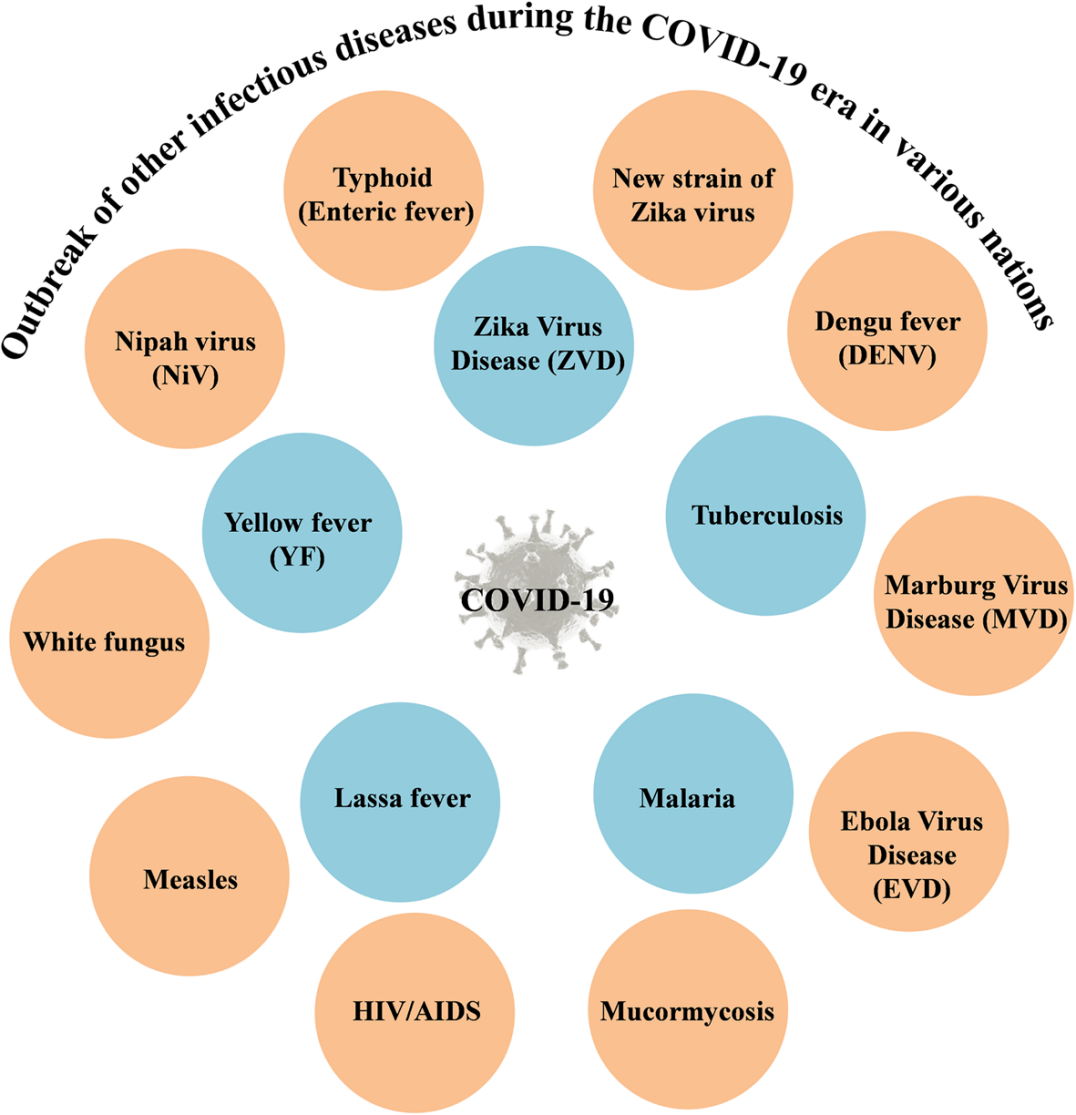
Different characteristic viral diseases outburst during COVID-19 such as Zika, TB, Malaria, Lassa fever, Yellow fever, Nipah, Typhoid, new African’s strain of Zika, Dengue, Marburg, Ebola, Mucormycosis, HIV, Measles and White fungus

Several diseases have emerged or reemerged during this pandemic which has burdened the health care system as well as the people around the world. Also diseases like HIV, Japanese encephalitis, Legionnaires’ and West Nile fever diseases etc., have resurged (Fig. 7). A detailed list of all diseases including Lyme disease, malaria and TB that have reemerged (Supplementary Table 2) The US CDC and the WHO have directed many campaigns to entirely get rid of hazardous diseases, for example smallpox, an extreme infective virus causing disease which was removed through vaccination campaigns in 1980. In fact that had build optimistic conditions and trust to fight and regulate many such diseases in the world. But, plague, yellow fever, influenza, and others have known to be emerging infectious diseases (EIDs) and re-emerging infectious diseases (REIDs) [46, 47]. Previously the EIDs never upsurge in humans; but troubled very few people in remote areas; otherwise have ensued in human life then have merely known as distinctive diseases else due to a novel transmuted strains. In accordance to US CDC, the EIDs are identified as diseases where the occurrence rate increased in humans over the years, affecting limited health issues either nationally or globally. Whilst, REIDs are known as diseases, after weakened vibrantly remain just reappearing, which causing main health problems.

Albeit maximum control and inhibition steps may employed, both infectious diseases are quiet onset sporadically and exhibit fears and burdens to societal welfare. Representative examples of human EIDs and REIDs are presented, wherein they influenced through various characteristic features of human activities, microbial adaptation, ecosystem, globalization, and public health infrastructure. Furthermore, these characteristic factors majorly associated with the increasing amount of human populations, overcapacity in cities by poor sanitary condition, rapid and high international traveling, variation of food habits during usage / processing and increased human’s exposure towards microbial supporting vectors and reservoirs in nature.

### SARS-CoV-2 variants

All viruses, including SARS-CoV-2, leads to COVID-19, transform over time. Most variations have little to no impact on the virus’ properties, however, some changes may influence like how simply it spreads, the related disease severity, or the activities of vaccines, therapeutic drugs, diagnostic tools, or other public health and social measures. The WHO, in association with partners, expert networks, national authorities, institutions and researchers have been monitoring and assessing the evolution of SARS-CoV-2 since January 2020. During late 2020, the emergence of variants that posed an increased risk to global public health prompted the characterization of specific Variants of Interest (VOIs) and Variants of Concern (VOCs), in order to prioritise global monitoring and research, and ultimately to inform the ongoing response to the COVID-19 pandemic (Table 1) [48].

**Table 1:** Presently labelled variants of concerns (VOCs)

WHO label	Pango lineage^a^	GISAID clade	Next strain clade	Additional amino acid changes monitored°	Initial known samples	Designation Date
Alpha	B.1.1.7	GRY	20I (V1)	+ S:484 K, + S:452R	United Kingdom, Sep-2020	18-Dec-2020
Beta	B.1.351	GH/501Y.V2	20H (V2)	+ S:L18F	South Africa, May-2020	18-Dec-2020
Gamma	P.1	GR/501Y.V3	20 J (V3)	+ S:681H	Brazil, Nov-2020	11-Jan-2021
Delta	B.1.617.2	GK	21A, 21I, 21 J	+ S:417 N, + S:484 K	India, Oct-2020	VOI: 4-Apr-2021 VOC: 11-May-2021
Omicron^b^	B.1.1.529	GRA	21 K, 21L, 21 M	+ S:R346K	Multiple countries, Nov-2021	VUM: 24-Nov-2021 VOC: 26-Nov-2021

Only found in a subset of sequences;

Omicron SARS-CoV-2 variant: a new chapter in the COVID-19 pandemic;

Omicron has some deletions and more than 30 mutations, several of which (eg, 69–70del, T95I, G142D/143–145del, K417N, T478K, N501Y, N655Y, N679K, and P681H) overlap with those in the alpha, beta, gamma, or delta VoCs. These deletions and mutations are known to lead to increased transmissibility, higher viral binding affinity, and higher antibody escape. Some of the other omicron mutations with known effects confer increased transmissibility and affect binding affinity

^**a**^Includes all descendent lineages. See the *cov-lineages.org* and the Pango network websites for further details

^**b**^See TAG-VE statement issued on 26 November 2021. Omicron includes Pango lineage B.1.1.529 and descendent Pango lineages BA.1, BA.1.1, BA.2 and BA.3. Omicron-defining constellation of mutations fully overlaps with Pango lineage BA.1, as this accounts for the vast majority of Omicron sequences to date. Most evidence we have to date about VOC Omicron is therefore based on Pango lineage BA.1. As of 24.01.2022, the BA.2 descendent lineage, which differs from BA.1 in some of the mutations, including in the spike protein, is increasing in many countries. Investigations into the characteristics of BA.2, including immune escape properties and virulence, should be prioritized independently (and comparatively) to BA.1

## Prevention and treatment of COVID-19

On account of known elevated abrasion rates, extensive costs and time-consuming paced discovery with development of new drugs and, potential repurposing of previously approved drugs for the treatment of both general and unusual dreadful diseases is ever-increasing to become an appealing suggestions, since it involves to employ for reduce the risk factor with improve lower efficacious compounds costs and shorter growth timelines. Obviously this drug repurposing factor [49–51] (https://covid19.who.int/table) attributes the main options to arise rapidly with a new treatment along with many safety data and other study outcomes are already available for the molecule, which allows substantially to accelerate the drug development process. Mainly the potential regimen against the coronavirus, COVID-19 falls in to two categories such as.

• Treatment for the respiratory symptoms
• Inhibitors for viral growth

In fact, vaccines [52] are anticipated to formulate prior immunity to a patient and also preventing the future disease threat as much as important, however, the drug repurposing factor embark upon to fight against the coronavirus outbreak and its related tribulations are considered for a future prolonging strategy rather than an emergency measure.

### Significant features of vaccines

**Fig. 8:**
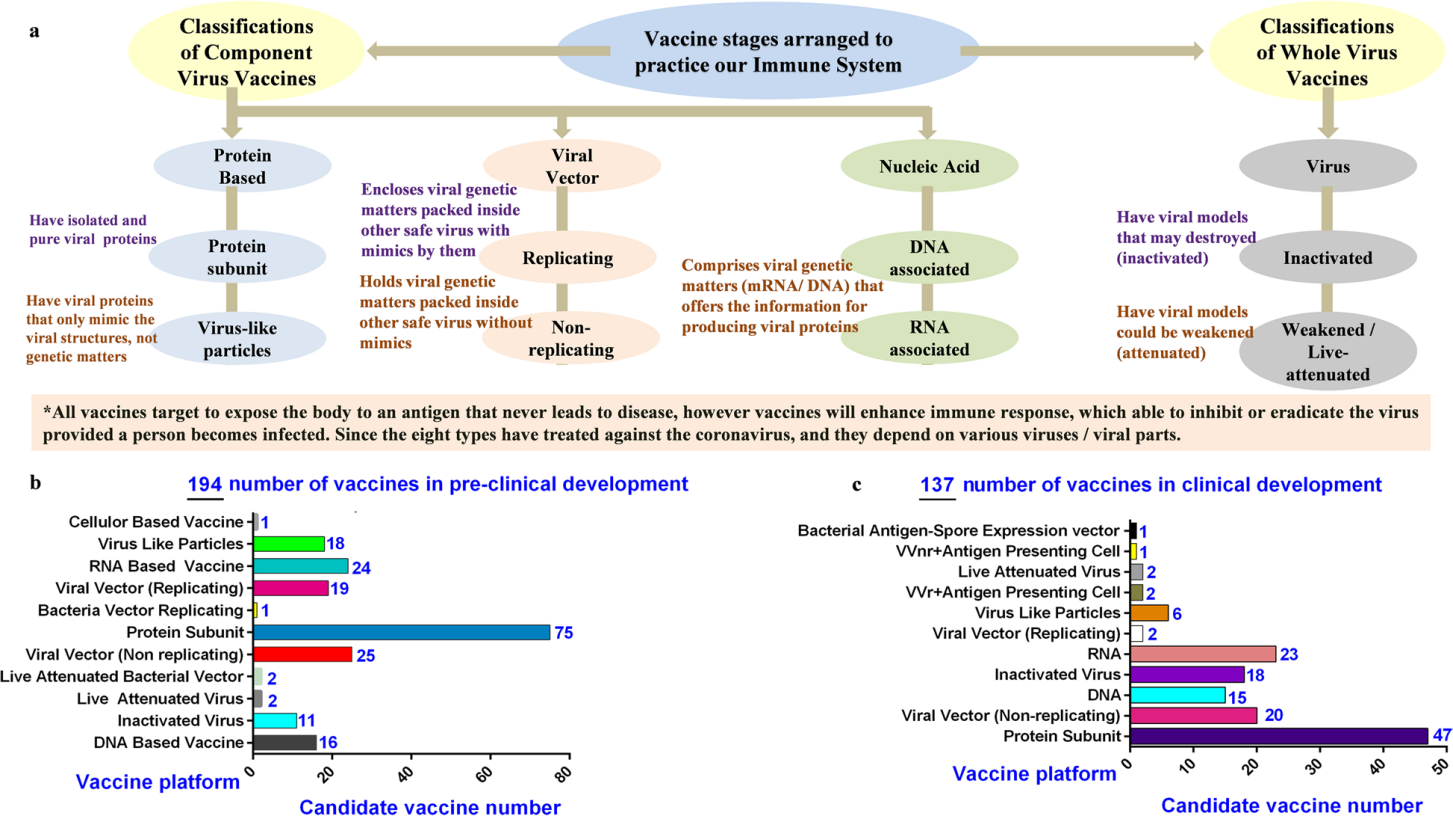
Representative vaccines platforms are arranged to train our immune system as classified in to **a** Component Virus Vaccines with sub divisions of protein based as protein subunit and virus like particles; further viral vector as replicating and non-replicating; nucleic acid as RNA based and DNA based; **b** Whole Virus Vaccines with sub divisions of Virus as inactivated and weakened*/*live-attenuated; **b** Number of vaccines in pre-clinical stage (194); **c** Vaccines under clinical development (137)

Despite, various characteristics of vaccine including the classifications (Fig. 8a), incurred for COVID-19, it is still increasing to get the pandemic under control through vaccine-induced herd immunity. Apart, the number of different types of candidate vaccines for clinical as well as in preclinical examination represented in (Fig. 8b-c). Range of vaccines clinically approved by WHO* (Supplementary Table 3). An effort of vaccination implies the extraordinary scientific analysis of viral genomic sequences to plan, manufacture, clinical examination, and got approval in short duration has involved, gratifyingly nanotechnology application prompted in rapid progress for vaccine development. These technology enable to provide nucleic acid and confirmation-stabilized vaccines subunit to regional lymph nodes, which induce active humoral and cellular immunity can inhibits viral disease or control its severity. Moreover the strategically unique cationic lipid and virus-simulating nanoparticles successfully carried out S protein delivery at exact cognate immune system with adjuvancy and B-, T-cell interactions in lymph node germinal centers, using epitope-based vaccines [53, 54] (https://www.who.int/en/activities/tracking-SARS-CoV-2-variants/).

Conversely current vaccine efforts demonstrated the short-term efficacy and safety, now the key concerns are its durable and adaptable nature against viral variants. Researchers also insights such factors to overcome immune release by viral variants and dignified the influence of nano-enabled COVID-19 vaccines improvement against other infective pathogens that may lead to future pandemics [55].

#### Nanotechnology based applications of vaccines

Nanotechnology occupied a significant pathway in the vaccines domain [56]. Nanoparticles (NPs) are useful to refine their stability by protecting the encapsulated mRNA from ribonucleases and enable distribution of intact mRNA towards the target region. Such mRNA based vaccines unveil ~ 95% efficacy in clinical trials (phase III), due to their distinctive nanocarriers as lipid nanoparticles (LNPs).

#### Nano vaccines

During 1990s, preclinical mRNA-based vaccines and therapeutic viability was reported which suggested the practical achievement of this method. Initial studies confronted major problems like pronounced innate immune response against foreign RNA, fast degradation in RNases, and incapable in vivo distribution to transformational equipment of the host target cells. Research indicated that exogenous RNA induces innate immunity partly by triggering of endosomally confined Toll-like receptors (TLRs), as well as the RNA immune activity can be distorted by impact of transformed nucleosides, such as m5C, m6A, m5U, s2U, or Ψ. They upsurge the safety profile and the mRNA expression efficacy in vivo. The advancement of good-yield in vitro of transcription structures for RNA making, routes for expanding translation by synthetic 5’ cap structural and crowning enzymes, developments in template design and purification, made this technology accessible. In vivo studies with nanotechnology tools for encapsulating mRNA into virus-sized (∼100 nm) cationic lipid nanoparticles (LNPs) led to the escape of these from extracellular RNases, enabling uptake and endosomal release of mRNA in target cells. Moreover the intra- dermal, muscular, or subcutaneous transfer of mRNA in cationic LNPs resulted in extended antigen expression and the stimulation of B-cell and CD4 + T follicular helper (TFH) cell responses which activate the germinal cores of secondary lymph organs, resulting in prolonged antibody responses. Also, mRNA vaccines encourage CD8 + T-cells too to identify and eradicate antigen expressing cells, including SARS-CoV-2 infected cells. mRNA-based nanoparticle vaccines by Moderna Inc. and Pfizer/BioNTech have now entered the market. Likewise the researchers described that the nano-facilitated routes for direct supply of protein subunits containing vaccine, as developed by Novavax, along with in silico based self-assembling NPs, which mimic the virus presence of the RBD provides a range of existing and next-generation vaccines that are currently available for COVID-19 or those already in clinical trials according to WHO (Supplementary Table 4). The remarkable achievement of those aforesaid mRNA based vaccines in phase III clinical trials (with ~ 95% efficacy) have been attributed to their unique nanocarriers, namely "lipid nanoparticles" (LNPs). Since they have bilayered liposomes they impart added stability to the loads. The rigid morphology, assists in good cellular permeability. The antigen might be loaded inside or on the surface of nanocarriers. The two nano vaccines in the market may address COVID-19 as well as pave the way for nanomedicine for treatment against viral diseases.

These vaccines are suitable in countries which have good low temperature storage conditions. At high / room temperature, mRNA based vaccine has poor stability, and so Pfizer-BioNTech vaccine should be maintained at − 80 °C to − 60 °C and the Moderna vaccine at − 25 °C to − 15 °C to circumvent mRNA degradation due to LNPs. Further, the mRNA vaccines have less/normal side effects during usage such as pain, fever, anxieties, fatigue, headache, muscle- and joint- pains. Of course there has been allergic responses due to PEG in the formulation. Meantime the adverse effects, safety duration and very low temperature storage are serious issues, which need to be careful considered for nanovaccines [57–60].

#### Sex and gender related toxicity and COVID-19 vaccine side effects

Based on clinical research, vaccines unveil variances between males and females, where vaccine response contains the immune response with probability of adverse events (AEs), and both are biased by sex. Women have identified as stronger immune responses against the foreign antigens (lead by vaccines and infections) in addition to self-antigens (a sensitivity to autoimmune disease) than men, immune responses differ above the life time, also women possess highly active innate and adaptive responses, T- and B- cells than men. Typically the genetic influences are liable to the female immune response initiate with the X chromosome, whereof women have the XX chromosomes, even they comprises many genes like ACE-2, that control immune and cellular function. MicroRNAs accelerate immunity and are more amount be present in women, also they recognize the valuable outcome of partial inability of several genes on the X chromosome. Sexual steroid based hormone differ over the periods, impact the immune response of female through hormone receptor spots interaction on major immune cells, indicating immune paths, and increasing gene expression. The sex hormone of estrogen lower the rate of ACE-2 receptor and though some of the researchers have distinctly continuing their extensive studies of the hormonal activities amenably on vaccines [61].

### Role of various antiviral drugs for COVID-19

**Fig. 9:**
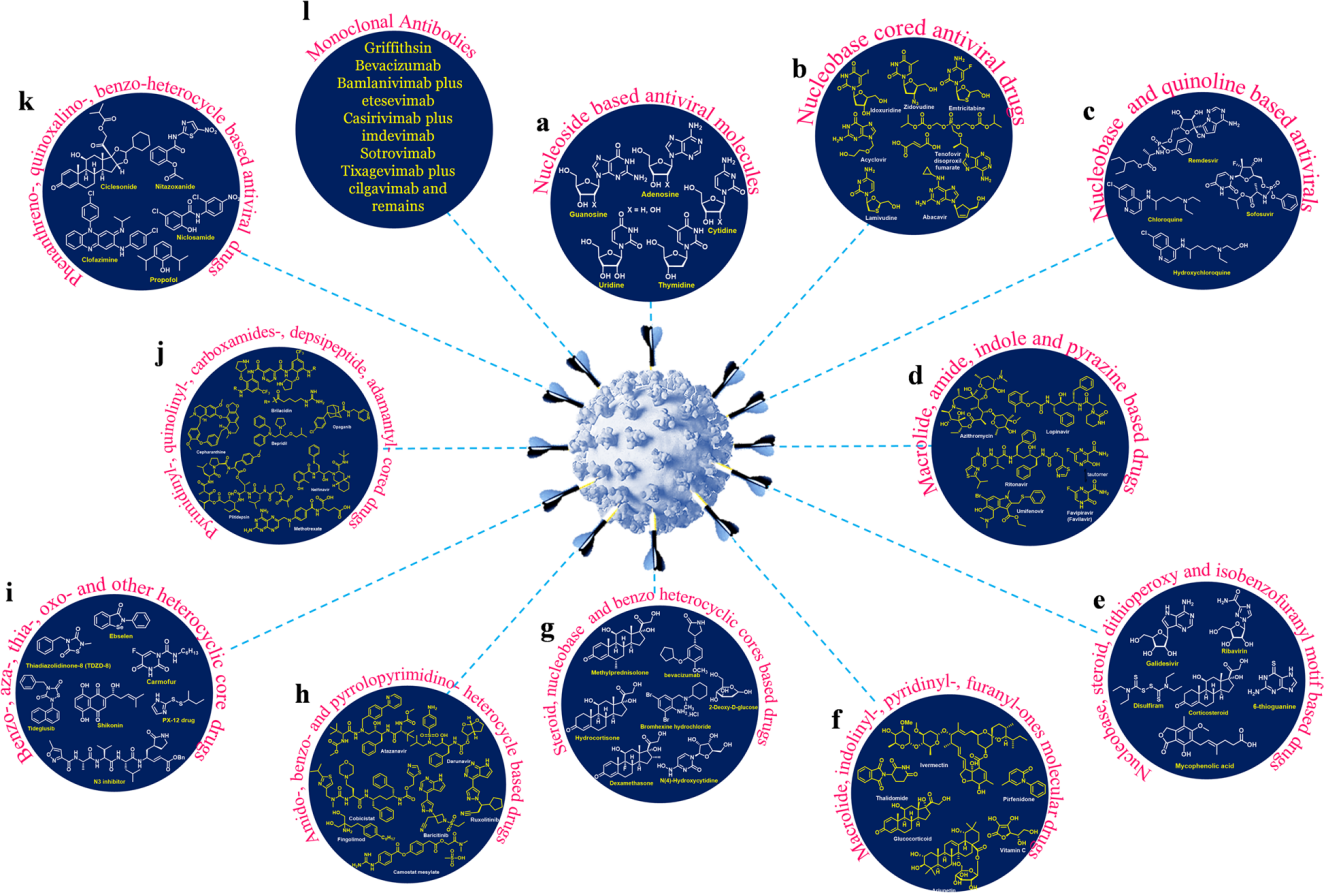
Illustrative examples of antiviral drugs and monoclonal antibodies assembly along with distinctive frameworks including **a** Nucleoside based antiviral molecules: adenosine, guanosine, cytidine, thymidine, and uridine entities; **b** Nucleobase cored antiviral drugs: idoxuridine, zidovudine, emtricitabine, lamivudine, abacavir acyclovir and tenofovirdisoproxil fumarate; **c** Nucleobase and quinoline based antivirals: remdesvir, sofusbuvir, chloroquine and hydroxychloroquine; **d** Macrolide, amide, indole and pyrazine based drugs: azithromycin, lopinavir, ritonavir, umifenovir and favipiravir (favilavir); **e** Nucleobase, steroid, dithioperoxy and isobenzofuranyl motif based drugs: ribavirin, galidesvir, corticosteroid, disulfiram, 6-thioguanine and mycophenolic acid; **f** Macrolide, indolinyl-, pyridinyl-, furanyl-ones molecular drugs: arjunetin, ivermectin, thalidomide, glucocorticoid, pirfenidone and vitamin C (ascorbic acid); **g** Steroid, nucleobase and benzo heterocyclic cores based drugs: methylprednisolone, bevacizumab, bromhexine hydrochloride, 2-deoxy-2-D-glucose, dexamethasone, and β-d-N4-hydroxycytidine; **h** Amido-, benzo- and pyrrolopyrimidino- heterocycle based drugs: atazanavir, darunavir, cobicistat, ruxolitinib, baricitinib, fingolimod and comostat mesylate; **i** Benzo-, aza-, thia-, oxo- and other heterocyclic core drugs: Ebselen, Disulfiram, Tideglusib, Shikonin, Carmofur, PX-12, thiadiazolidinone-8 (TDZD-8) and N3-inhibitor; **j** Pyrimidinyl-, quinolinyl-, carboxamides-, depsipeptide, adamantyl- cored drugs: brilacidin, opaganib, nelfinavir, bepidril, plitidepsin, methotrexate and cepharanthine; **k** Phenanthreno-, quinoxalino-, benzo-heterocycle based antiviral drugs: propofol, clofazimine, niclosamide, nitazoxanide, ciclesonide and **l** Monoclonal antibodies: griffithsin, bevacizumab, bamlanivimab plus etesevimab, casirivimab plus imdevimab, sotrovimab, tixagevimab plus cilgavimab

Despite, many of the clinical assessments be currently in progress to check the potential remedy, the global reaction on the COVID-19 pandemic has been mainly inadequate to screening/inhibition. With respect to that, some of the common potential antiviral drugs under consideration, for instance, several nucleoside analogues [62] associated with characteristic sugar unit and nucleobase, whereas, the nucleotides are referred as nucleosides have minimum one phosphate (or phosphate-like) moiety. Though nucleosides abundantly found in nature, the five major components be present in DNA, RNA and additional bioactive ingredients consist of adenosine, guanosine, cytidine, thymidine, and uridine entities (Fig. 9a). Accordingly, even if little modification in structure behold intense effect, the ideal nucleo(s)tide scaffolds follow the natural nucleoside arrays comprising enzymes associated cell- or viral- organisms, despite the fact that owe to the structural arrangement variation direct to interruption and/or completing the reproduction or biological processes.

#### Nucleobase core antivirals

Adopting this nucleoside based structural motif have encountered the nucleobase tethered antiviral drugs of Idoxuridine, Zidovudine, Emtricitabine, Lamivudine, Abacavir Acyclovir [63] and Tenofovirdisoproxil fumarate (Fig. 9b) [64] that inhibiting the viral DNA replication, also engaged in bio- and photo-transformation processes.

*Remdesivir*: An antiviral drug is examined for treatment for COVID-19 specifically, and also subjected an Emergency Use Authorization (EUA) in the United States of America for the individuals who are hospitalized with acute illness. This drug [65] may reduce the time duration to retrieve from the disease and moreover the treatment provided intravenously. It was initially developed for Ebola and Marburg virus diseases, it was unsuccessful for those diseases. However (GS-5734), it is an effective inhibitor of viral RNA-dependent RNA polymerase [66] through in vitro activity against both SARS- and MERS-CoV, which has also known for therapeutic COVID-19 candidate due to its SARS-CoV-2 inhibitory property through in vitro.

*Sofosbuvir*: A uridine monophosphate based prodrug, targeting HCV ns5b, designed for the regimen of chronic HCV infectious disorder [67]. Since this nucleotide analogue proceeded through interaction with the catalytic position of HCV (ns5b polymerase) enzyme. Both remdesivir & sofosbuvir specified the scaffold protection for the catalytic position involving COVID-19 pneumonia causative virus nsp12 and HCV ns5b polymerase. Quinoline based antiviral drug Further the FDA (Food and Drug Administration) approved anti-malarial drugs such as chloroquine and its derivative as hydroxycholoroquine (Fig. 9c) for treatment against coronavirus due to the emergency reason. Despite, they have been utilized to other diseases of malaria and many inflammatory problems, in addition, the systemic lupus erythematosus (a chronic disease in auto-immune system) and even rheumatoid arthritis. It occurs to us that the merits of both drugs are reasonably safe, inexpensive, relatively lacking of side effects, and availability in all drug stores. Moreover chloroquine is better treatment controller in reducing the degeneration of pneumonia, enhancement of lung imaging results, removing the virus and decreasing the time interval of the disease. Therefore the chloroquinone is an approved malarial drug also under investigation to utilize for an efficient drug source for eradication of COVID-19 significantly [68].

#### Macrolide peptide, indole and pyrazine based antivirals

Based upon the clinical arrangement of azithromycin and hydroxychloroquine, both drug molecules added for the treatment and may lead an efficient role for virus elimination [69, 70]. Then the two protease inhibitors of lopinavir and ritonavir [71] were developed for HIV treatment, recent research study suggested that ineffective to display clinical benefits or minimize the virus population of many patients infected with COVID-19. Similarly, the combination of lopinavir/ritonavir with umifenovir clinically shown apparent positive results than divided lopinavir/ritonavir prologues [72, 73]. The umifenovir is a hemagglutinin inhibitor particularly inhibit the viral fused host cell membrane, viral- DNA and RNA synthesis, also developed the interferon production, and regulating the immune system. Thus combination of drugs with umifenovir may exhibit the enhancement of antiviral efficiency with different mode of actions, though the substitute may cause the potential adverse side effects during treatment. Umifenovir antiviral drug used for potential treatment of influenza virus, since the efficiency and protection of this drug component for COVID-19 is under investigated. Accordingly the favilavir drug [74] fight against RNA infection by inhibiting the RdRp viruses of Chikungunya, Ebola, Enterovirus, Influenza, Norovirus and Yellow fever are reported which could also active against novel SARS-CoV-2 (Fig. 9d). This was the preliminary antiviral drug approved by National Medical Products Administration of China that may have the possibilities to treat for COVID-19, but undeclared for this current pandemic. Obviously this drug is generally used for influenza treatment in Japan and China. Identify the efficiency of favilavir, many clinical trials are on track to eliminate the COVID-19 in global. Since, favipiravir (brand name Avigan) an anti-flu drug was developed by Japanese company Fujifilm also COVID-19 make use of this enzyme to reproduce and is categorized into the identical single-stranded RNA virus as influenza; for this reason, Avigan may be an efficient drug source and also believed that in treating COVID-19. Its preparation and supply is such a preference of Japan’s Health, Labor and Welfare Ministry. As a result, it has not at all circulated in to the medical stores, hospitals and dispensary in Japan or abroad [75, 76].

#### Nucleobase, steroidal and other drug inhibitors

In this streamline, the ribavirin, a guanine analog, possess antiviral activity against few animal coronavirus, and in the SARS-CoV outbreak, ribavirin together with corticosteroids develop into a typical treatment for the exploitation of SARS-CoV, further the in vitro analysis not shown the efficiency of ribavirin against coronavirus, instead it cause few side effects like hemolytic anemia, hypocalcemia, and hypomagnesemia. Subsequently, studies of many patients revealed that combined usage of ribavirin and corticosteroid even showed a raise in viral influence [77, 78], so its applications are rejected over a period.

Similarly, the other important agent of galidesivir (adenosine based analogue) developed for HCV is now in nascent stage clinical test, for calculating their safety nature in healthy subjects along its potency antagonistic yellow fever, exhibiting anti-viral properties in opposition to many RNA viruses, containing SARS and MERS but these are not evaluated for 2019-nCoV till now. It was initially planned to treat against hepatitis C virus, (HCV), later on, executed for the inhibition of dreadful filo viral disease like ebola and marburg viral syndromes, further, it also possess antiviral efficacy against diverse RNA virus clusters, which including arena-, bunya-, corona-, flavi-, paramyxo- and phlebo- viruses. Distinctly it was approved for Hepatitis C Virus (HCV) and Respiratory Syncytial Virus (RSV), may be directed whether an orally, or intravenously and through inhalation [79–83]. Disulfiram, notably plays a significant allosteric inhibitor with respect to the specificity of MERS-CoV, additionally, the manifold inhibition assessment sustained a kinetic method, amid disulfiram combined together with 6TG (6-thioguanine) and/or MPA (mycophenolic acid), (Fig. 9e) be able to collectively reduce the MERS-CoV papain-like protease. Therefore, the recombination of these such medicinally offered drugs may be useful to treat against SARS-CoV-2 [84].

Ivermectin is one of the FDA approved drugs for parasitic infections, in fact, this antiviral agent employed for an effective treatment for chikungunya and yellow fever, showed promising activity for treating Dengue and utilized as drug repurposing efficiency, which may be responsible for inhibition of the COVID-19 [85]. Generally, thalidomide with its combinations of low-dose glucocorticoid leads to exhibit immunomodulatory activities, owed to the potential regulating immune system of thalidomide entities might be the cause for the existence of the inhibition properties as the inflammatory cytokine surge, reduce anxiety to decrease oxygen utilization, relief from vomiting and lung transudation activities [86–88]. Therefore these interesting factors envisaged by the researchers and well documented that the host immune reactions be significantly surviving to critical ARDS in CoV infected patients, which ought to be useful therapeutic strategy for the treatment of severely infected SARS-CoV-2 caused pneumonia patients. In fact, thalidomide has an anti-inflammatory activity caused by its potential rapidity of the mRNA deficiency within blood cell, decrease the tumor necrosis factor-*alpha* (TNFα), enhance the interleukins secretion as represented as IL-12, and triggering usual killer cells. Senthil et al. concluded that Arjunetin acts an efficient protease inhibitor of SARS-CoV-2 that is comparable to Lopinavir and Remdesivir antivirals [89–91] and also assisting as a drug aspirant against SARS-CoV-2 (Fig. 9f). On the other hand, the methylprednisolone (synthetic corticosteroid) have examined in case of COVID-19. Since, the vitamin C (an antioxidant) also known as ascorbic acid, bevacizumab, bromhexine hydrochloride fingolimod, pirfenidone, were under investigation for COVID-19 treatment; amongst the vitamin C might be having oxidative stress and inflammation minimizing properties, simultaneously acts as good enhancer- of vasopressor (antihypotensive agent), immune system, endovascular function, and epigenetic immunologic alterations. Clinical assessment have been recognized, if provided potential data underlying on mortality development in sepsis, and more systematic trials be desirable to substantiate those conditions. Pirfenidone drug suggested for the idiopathic pulmonary fibrosis (IPF), which is a type of lung diseases, in addition, the anti-inflammatory and antioxidative effects, are characteristic inhibition property of IL-1β and IL-4 (Interleukins). Thereby proper examination of pirfenidone having anti-inflammatory property conceivably supportive to participate against an infectious SARS-CoV-2 ailments. Researchers demonstrated that the corticosteroid containing methylprednisolone, dexamethasone, and hydrocortisone has high therapeutic value for treatment for SARS-CoV patients. Bevacizumab. An example for tailored monoclonal antibodies (mAb) that endeavors in Vascular Endothelial Growth Factor (VEGF) along with it might be decreasing the intensities of VEGF attributable to hypoxia, long-lasting chronic inflammation, and stimulate the typical respiratory tract epithelium infection, these entire things may prevent the dissemination of the edema (swelling or inflammation, at injury) in patients affected with COVID-19. Bromhexine**.** One of the members of type II transmembrane serine protease inhibitors (TTSP) plays a pivotal role in development of cell growth, liable for S-glycoprotein the activation in SARS-/MERS- coronavirus in aid of viral access throughout the plasma membrane. Fingolimod**.** An immunomodulatory drug candidate, generally designed for the multiple sclerosis (MS) treatment, some of the clinical studies were underlying to assess the efficiency of bromhexine combinations through usual treatment and/or typical treatment to infected COVID-19 patients. Since, it is an obvious immunomodulator containing sphingosine l-phosphate receptor masking lymphocytes, lessening the immune cell actions may results nerve injuries. The *β*-d-N4-hydroxycytidine, acytidine based ribonucleoside possess wide-ranging of antiviral activity against many viruses such as Chikungunya Virus (CHIKV), Respiratory Syncytial Virus (RSV), Venezuelan Equine Encephalitis Virus (VEEV) Influenza A and B (IAV/IBV) Viruses, Bovine Viral Diarrhea (BVD) Virus, followed by Corona Viruses (CoVs). Since NHC exhibit high potential antiviral effect, initially by mutagenesis of viral RNA genomic sequence owed to presence of NHC directing minimal resistivity for VEEV, however the NHC apparently holding the characteristic high resistance barrier for RSV, IAV and BVD viruses. Significantly, the NHC presented an effective anticorona viral activity, especially for SARS and HCoV-NL63, nevertheless the CoV inhibitory mode of action not been distinguished. Since, the combination of remdesivir along with NHC may suggest a novel way of interaction with the CoV replicase. The potential antiviral effect shown by NHC deserved for more intrinsic studies and for the management of CoV infections, whether singularly or combined with other direct‐acting antivirals (DAAs) and immunomodulators. 2-Deoxy-D-glucose a potential drug source used for the inhibition of glycolysis and glycolytic enzymes, wherein it abolishes the replication of viral particles and cytokine response employing glycolysis as a significant upstream occasion through SARS-CoV-2 pathogenesis (Fig. 9g), while severe glycolytic intermediate and/or end product associated such result was not concluded [92–96].

### Non-enzymatic and enzymatic inhibitors

Due to lack of approved pharmacological treatments, various antiretrovirals (Fig. 9H), including invariable combination of drugs like darunavir/cobicistat, are utilized in the hospital wards with off-label manner as potentially critical medicines for COVID-19 patients. Unfortunately, the desired medicines insufficient to infected patients in emergency situation. So the combinations of drugs were highly influenced to the drug quality, therefore, as mentioned in the EU as cobicistat a technique-based inhibitor of cytochrome P450 (CYP) 3A enzymes, which being a pharmacokinetic booster for the human immuno deficiency virus (HIV-1) protease inhibitors such as atazanavir and darunavir in adult. Cobicistat may enhance the serum creatinine level (probably through inhibition of renal tubular transporters) and as a result reducing the estimated glomerular filtration rate (GFR), distinctly the fixed-dose formulation combination of cobicistat with darunavir and atazanavir are in development for potentially medicines for COVID-19 patients. Currently the combination of cobicistat with darunavir drugs received considerable attention from the United States Food and Drug Administration (FDA) approved for the COVID-19 pneumonia infected patients accordingly, and such a combination already provided for HIV treatment. Moreover, darunavir acting as an additional inhibitors of HIV protease, and cobicistat as a representative enhancer for development of the pharmacodynamics and pharmacokinetics of darunavir through cytochrome P450 (CYP3A) inhibitory effect [97, 98]

Several critically sickened COVID-19 patients on treatment with high dosage of intravenous (IV) vitamin C in anticipation to facilitate the hasten recovery. Nonetheless, there is no obvious scientific facts to get rid of this severe illness, and even it is not a standard ingredients for the remedy and moreover the research is under progress in China to determine whether this treatment is valuable for severe COVID-19 infected patients, the results that are expected in the descent, indeed, the previously utilized high-dose intravenous vitamin C which might facilitate in overwhelming infections. The modern study initiated that strong intravenous dosage of vitamin C treatment (together with corticosteroids and thiamine) to sepsis and acute respiratory distress syndrome (ARDS), However, neither of these studies emerged to vitamin C therapy for COVID-19 affected patients and in major, these are the common conditions leading to emergency admission, oxygen mask support, or decease between severe COVID-19 infections. Concerning safeguard, no evidence that using vitamin C will facilitate to prevent illness with the virus that causes COVID-19 [99, 100].

#### Significances of fingolimod-sphingosine 1-phosphaste receptor modulator

Fingolimod, a sphingosine-1-phosphate receptor, which regulating the cell–cell bond and cell–matrix bond, also mentioned as an efficient immunomodulator, in particularly regimen for the Multiple Sclerosis (MS) infected patients with deterioration cases, the MS is a distinctive high potential disability illness in the central nervous system (brain and spinal cord); wherein the MS leads to the Secondary Progressive Multiple Sclerosis (SPMS) followed by Relapsing–Remitting Multiple Sclerosis (RRMS). These types of disorders worsening the person's ability and gradually changed in to dreadful condition. Substantially, this drug ought to be identified as to prevent the growth of the severely infected patients with acute respiratory distress syndrome (ARDS), and thus, at present considerable attention towards the direction of a high threatening COVID-19 pneumonia infections. In accordance to that the several pathological results of hyaline membrane development, pulmonary edema, multiple sclerosis along with ventilator maintenances made the research study of fingolimod under investigation to explore its worth for COVID-19 [101, 102].

#### Inhibitory effect of potential choloroquine towards anti-coronaviral activity

In the earlier demonstration of chloroquine and hydroxychloroquine, we just known that these two drug candidates are represented as antimalarial, and kept under clinical trials for SARS-CoV-2. Apart from this, these drug were secured with reasonable cost effective drugs for malarial pandemic regions. Intrinsically, this potent remedial source act against human coronavirus HCoV-OC43 in animal model, and SARS-CoV in cell culture analyses, since the anti-viral mode of actions of chloroquine is still speculative. However, this drug unaltered the outercell phase of angiotensin-converting enzyme 2 (ACE2), and moreover the biosynthetic/glycosylated-processes of the SARS-CoV spike glycoprotein respectively. In contrast, if the terminal glycosyl units of the ACE2 receptor was repaired, may led to viral interactions and thus, the chloroquine possessing anti-SARS-CoV activity in cell culture during viral uptake, manifold valuable mechanisms ahead with access of cells through endocytosis, factually the outer surface of entire virus particle containing spike glycoprotein have to be divided by residual endosomal proteases known as cathepsins. Also, the chloroquine- can interrupt the polyproteins as ORF1ab, ORF3a and ORF10 to strike the heme in phorphyrin ring system and also inhibit ORF8 and surfaces of glycoprotein to porphyrins up to specific extent, successfully alleviate the symptom of distress in respiratory system. Meanwhile favipiravir drug component might inhibit the enveloped ORF7a proteins, which combined with porphyrin ring to manage viruses from the penetrating host cells and catch freed porphyrins [103, 104].

#### Ruxolitinib- (JAK) inhibitor and its potential applications

Ruxolitinib (Rux), is an orally administered drug belongs to bioavailable Janus-associated kinase (JAK) inhibitor family with various potent activities like antineoplastic and immunomodulator. Specifically ruxolitinib binds with protein tyrosine kinases JAK 1 and JAK 2, inhibitors may direct to anti-inflammatory and preventing cellular proliferation activities. Similarly the JAK-STAT (Signal Transducer and Activator of Transcription) route act as a key role in the signaling of cytokines and growth factors also involved in hematopoiesis, and the immune response; JAK kinases may be synchronized in myeloproliferative, and various malignancy disorders. More extensive studies of Rux revealed the disease-related symptoms, splenomegaly development, available therapies in myelofibrosis, superior control of blood counts etc. and also suggested the treatment at the opening stage for polycythemia vera, hematological side effects like anemia and thrombocytopenia and moreover some case reports emphasized the potential risks of non-melanoma skin cancers, reactivation of hepatitis B virus, tuberculosis infections. Ruxolitinib, received an approval from US Food and Drug Administration (FDA) forsteroid-resistant acute graft versus host disease (aGVHD), and obviously this investigated data is projected to prevent the disregulated immune system caused by COVID-19, which engender the pneumonia and subsequent severe acute respiratory syndrome. According to the epidemiology, hitherto recommends that the COVID-19 is highly infectious, when compared with earlier SARS- or MERS-CoV, at present many mode of actions were known for SARS-CoV-2 infection and multiplication processes of existing goal used for pharmaceutical mediation, other infections state as macrophage (kind of phagocyte), pneumocytes (alveolar cells), and pulmonary mast cells or centinal cells needs virus containing spike protein, certainly, such invasion route connecting the spike protein accessory towards the ACE2 protein receptor stimulated through host cell is resulting trans-membrane protease serine 2 (TMPRSS2) inhibition. Since the inhibition of TMPRSS2 moderately blocked infectivity by SARS-CoV and human coronavirus HCoV NL63 in cell cultures. Further in vitro analysis demonstrated that camostat substantially decreases the Calu-3 lung cells infection by SARS-CoV-2 liability for COVID-19. Similarly the representative camostat mesilate inhibiting the enzyme may be functional in hindering viral host cell access. After that the virus ss-RNA (positive), is free from virus RNA replication and translation of virus polyproteins eventually divided into fully developed effector proteins by virus proteases [105, 106].

#### Baricitinib-JAK-NAK inhibitors

Baricitinib, a potent and selective Janus kinase, also known as JAK inhibitors or jakinibs therapeutically operating by inhibition of the Janus kinase enzymatic family, various types are available as JAK1, JAK2, JAK3, TYK2, and hence interfering with the signaling route of JAK-STAT accepted for indication like myelofibrosis and rheumatoid arthritis. Baricitinib exhibit strong anti-inflammatory, when JAK–STAT signal inhibitors effectively functioning against the results of complex cytokines (together with interferon-γ), which typically examined towards COVID-19 infected people. However the baricitinib drug inhibitor efficiency have similar to JAK inhibitor, also the superior attraction for Adaptor-Associated protein Kinase 1 (AAK1) represented that the best characteristic inhibition property, in addition baricitinib has recognized as a (Numb Associated Kinase) NAK inhibitor, due to strong bonding with AAK1 behavior as a key controller of clathrin (complex proteins array develop a coated endocytic vesicles) mediated endocytosis, the high efficiency baracitinib possess stronger interactions- with low plasma protein and negligible with CYP enzymes and drug transporters. Meanwhile the baricitinib combined with directly involving antivirals could minimizes the viral infectivity, viral replication, and the unusual host inflammatory action. Therefore researchers suggests the baricitinib drug might be a useful counteracting SARS-CoV-2 infections and also be suitable for clinical investigation [107].

In the year 2002, *β*-coronaviruses (CoV) caused three zoonotic epidemics, along with the new emergence of SARS-CoV-2 in 2019 was added. In the presence of spike protein pseudo virus arrangement, the researchers authenticated the human angiotensin converting enzyme 2 mentioned as hACE2, which is the SARS-CoV-2 receptor, direct in to 293/hACE2 cell mainly through endocytosis. Further research studies on recovered SARS and COVID-19 affected patients’ sera deficient cross-neutralization suggested us the recovered infectious syndrome from an individual may not defend against the other, which has resulted the present potential goals for improvement of drugs and vaccines for COVID-19.

Drug discovery research program used to identify new lead drugs target the COVID-19 main protease (M^pro^) that acts as a vital part in paramount viral reproduction and transcription, creating it as a fascinating drug targeted to virus. So they recognized N3 as typical mechanism supported inhibitor via computer aided drug designing and also characterized the structural arrangement through crystal studies of COVID-19 M^pro^ in multifaceted along with these chemical constituents.

#### Therapeutic study of small and macrolide antivirals

In accordance to the molecular array and their substituents of Ebselen, Disulfiram, Tideglusib, Shikonin, Carmofur, PX-12, thiadiazolidinone-8 (TDZD-8) and N3- are leading potential drug sources (Fig. 9i), which inhibit the activity of COVID-19 virus M^pro^, also exhibited strong antiviral activity in cell-based assays [108].

In the recent investigation of COVID-19 targeting drugs, such as plitidepsin, revealed that the proteins present in virus associates through the eukaryotic changing mechanism, and the transformed inhibitors contains efficient antiviral properties. White et al. identified that the plitidepsin drug has inadequate clinical authorization, however maintains the antiviral character of 90% at IC = 0.88 nM, which is more effective than remdesivir against novel coronavirus in vitro (factor of 27.5), with partial toxic in cell culture analysis [109]. By the drug-resistant mutant utility exhibiting the antiviral activity of plitidepsin initiated by inhibition of the identified target eEF1A and also described the decreasing order of viral replication in the lungs using prophylactic treatment via in vivo efficacy, herein this results indicated as one of the promising therapeutic candidates against COVID-19. The other significant drugs (Fig. 9j) like lamprene (clofazimine), is used for leprosy with safer and pharmacokinetics profile, shows inhibitory activity for pan-coronavirus, and have antagonizing capability of viral replication in numerous in vitro systems, comprising the beginning stem cell-derived cardiomyocytes in human and ex vivo lung cultures. Since the approved drugs by FDA has the characteristic inhibitory property with several phases of viral replication, signifying various essential antiviral characters. While SARS-CoV-2 pathogenesis of prophylactic/therapeutic management to a hamster model considerably minimized the viral capacity in the lung and fecal viral shedding and moreover prevented the cytokine storm linked with viral infectivity through a concomitant utilization of clofazimine drug. Clofazimine showed an interaction once directed with remdesivir. Some of the factors are influenced on clofazimine is oral bioavailability, low production cost, an attractive clinical aspirant for the treatment of outpatient and remdesivir-supported combinatorial therapy for COVID-19 patients emergencies. Therefore clofazimine may control the present and future pandemicities [110]. *Brilacidin*: A host defense peptide mimetics (novel anti-infective), established for the treatment of eye infections, it resembles the characteristics of defensin (a naturally occurring cysteine-based cationic proteins). Globally there is an emergency requirement towards the therapeutically involved approaches, which should be arranged for safer treatment of COVID-19 and minimize the concomitant mortality and morbidity. Hence the researcher suggested that this defensin-mimetics brilacidin may prevent SARS-CoV-2 and overlay the way for the potential clinical utility as therapeutic profiles and the biomedical applications in a human lung cell line (Calu-3) and a monkey cell line (*Vero*). In addition, brilacidin established synergism of antiviral property once combine with remdesivir potentially inhibit SARS-CoV-2 against few viral strains in cell culture [111] *Opaganib*: A sphingosine kinase 2-selective inhibitor examined for antiproliferative activity against different cancer cells, they are significant enzymes in the sphingolipid metabolism path that stimulates development of tumors and chronic inflammation especially for the treatment against cholangiocarcinoma. Since this compassionate drug opaganib utilized in a few group of COVID-19 patients, based upon the potential drug's activities against inflammatory and viral properties. This efficacious analysis leads to continued examination in a clinical Phase II trial [112]. *Nelfinavir* (NFV): It is one of the potential antiretroviral drug candidates used for the treatment for HIV infection; since it falls under the category of drugs called protease inhibitors (PIs), alike nelfinavir mesylate and its active metabolite M8, formerly, the intracellular accretion of protease inhibitors reported through in vivo analysis, showing in the following order: nelfinavir > saquinavir > ritonavir > indinavir. Some characteristic PIs is utilized in combined manner with other antiretroviral drugs. For example, cepharanthine with nelfinavir combination led to the active drugs to treat SARS-CoV, similarly nelfinavir has effectively targeted main protease (M^pro^) in SARS-CoV-2 pathogen [113, 114]. *Bepridil*: It (useful calcium channel blocker) is an example for antianginal, especially exhibit antiviral activities against SARS-CoV-2. It prevents cytopathogenic effect (CPE) stimulated through the nCoV in Vero, E6 and A549/ACE2 cells in lower micro molar (EC_50_) values. Previously, it provides a very high safety against Ebola viral infections for mice (dosage at 12 mg/kg). Owing to the emergency of COVID-19, this bepridil utility and its significant study motivates the succeeding investigation preclinically towards animal models to make unambiguous its direction for COVID-19 patient’s clinical usage [115]. *Methotrexate*: It is generally referred as the prime source for disease-modifying antirheumatic drugs (DMARD) for rheumatoid arthritis (RA) affected patients; it is an effective inhibitor (approved by FDA) of biosynthesis of purine, actively inhibiting (i) the replication of viral RNA, (ii) synthesis of viral protein, and (iii) discharge of virus. Further the methotrexate immunosuppressive effect is advantageous for RA patients to decrease biologically oriented immunogenicity, however, this drug ought to be highly potential in patients during early stages of symptoms with efficiently preventing the SARS‐CoV‐2 viral replication, transmission of the infectivity, and probable fatalities and though it might be reconciliation vaccine responses [116, 117].

*Griffithsin*: One of the protein varieties isolated from the red algae griffithsia, has sequential array of 121-amino acids exhibiting a Jacalin-like lectin fold. Many of the protein structures confirmed without any ambiguity by X-ray crystallographic analysis and perceived in the PDB; the red-algae –derived lectin binding with oligosaccharides on outer region of different viral under glycosylation with HIV-120 and SARS-CoV-2 spike glycoprotein [118].

### Anticoagulants for COVID-19 treatment

Paranjpe et al., found that the anticoagulants utility may develop the survival period for the most severely affected COVID-19 patients. Also their findings suggested that the systemic anticoagulation (AC) possibly associated with better results amongst COVID-19 infected patients. Though their research data study provided the proven insights for considerable thoughts on the supervision of COVID-19 infected patients, the potential randomized desirable clinical trials were used to determine the systemic AC presentation to a survival benefit in hospitalized COVID-19 infected patients. The result may facilitate the doctors and researchers, who have been demonstrated that if the coronavirus patients affected by blood coagulation disorders which can damage vital organs, therefore the doctors suggesting the utility of blood thinner medicines (anticoagulants) may assisting to prevent the blood clots, According to the New York City hospital study reported that the researchers observation for the incubated patients on treatment with anticoagulants leads a minimization of mortality rate from 29 to 63% per cent. Researchers suggested that the systemic anticoagulation may be associated with better effect among the patients hospitalized with COVID-19 [119].

#### Scientific explanations of airborne transmission

Heneghan research group have systematically reviewed and stated that if an infective viral particles transmits mostly by airborne droplets during respiration, the significant regulatory actions has to be taken against this airborne disease including respirational disinfection and dressing high-grade shield one for supposed aerosol-producing health-care protocols. Some of the guidelines not required to differentiate between internal and external, meanwhile a gravity-driven mode for spreading should be same for both situations. However, if this transmittable virus is mainly airborne, a person might be possibly be affected once they intake aerosols formed after infective individuals breathe out, speak, screams, singings, sneezing, or coughing. Falling airborne transmission of virus needs determination to circumvent breathing of infective aerosols with aeration, air purification, decreasing crowd and other factors. Since the airborne respiratory viruses spreading is very hard to exhibit straightly. The ten tributaries of proof cluster assisting the hypothesis in which the novel SARS-CoV-2 is spreading mostly through the airborne route [120]. *Actemra* (Tocilizumab): Basically it is an immunosuppressive drug administered through an intravenous infusion; this drug is used for treatment against rheumatoid as well as the systemic juvenile idiopathic arthritis. Further, it is a developed recombinant mAb (monoclonal antibody) act as an antagonist of Interleukin 6 (IL-6) receptor and afforded proven clinical interests under examination with severe COVID-19 pneumonia. In fact the various clinical studies confirmed the diagnosis factors and the other significantly estimated therapeutic values [121].

*Sotrovimab* (VIR-7831) GSK4182136: It is based on monoclonal antibody used for treatment against COVID-19 patients in a milder to moderate stage at more risk for demise / clinical treatment. Primarily it was isolated from memory B cells in a person recuperated from the SARS virus. The respective antibody aims the spike (S) protein that encourages the SARS-CoV-2 access into host cells and is the foremost objective of neutralizing antibodies [122].

*Bamlanivimab and Etesevimab*: Bamlanivimab (LY-CoV555/LY3819253) and Etesevimab (LY-CoV016/JS016) having synthetic antibodies responsible to neutralize entirely human and recombinant monoclonal antibody, since the targets and the mode of action of etesevimab is to neutralize SARS-CoV-2 by exactly binding to the surface of the spike protein. This high affinity binding later inhibits SARS-CoV-2 from binding to the host cell surface receptor ACE2. Etesevimab is currently being investigated against COVID-19. Moreover, the studies based on X-ray and Cryo-EM suggest that bamlanivimab interacts with RBD of the S protein site overlying the ACE2 binding position, which is available in both the up and down conformations of the RBD. Particularly, bamlanivimab impasses to the S protein with a K_D_ of 0.071 nM and blocks S protein-ACE2 connections with an IC_50_ value 0.025 μg/mL respectively [123, 124].

**REGEN-COV (Casirivimab and Imdevimab):** Casirivimab and imdevimab exist as two recombinant monoclonal antibodies of human IgG1 that binding non-viability to non-overlying spike protein epitopes in SARS-CoV-2 receptor-binding domain, in this manner preventing the viral entry into host cells. Later, the mixture of casirivimab-imdevimab obtained FDA approval from EUA meant for the high-risk patient’s treatment by mild to moderate COVID-19. These mAbs be formulated to interact to various sites on the spike protein. The mode of action of imdevimab is aiming the spike protein’s receptor binding domain of SARS-CoV-2, a protein act as a vital part in viral attachment, combination, and entry into the cell. Combined with casirivimab, imdevimab neutralizes the SARS-CoV-2 spike protein [125].

#### COVID-19 convalescent plasma treatment

This treatment has been practiced against infectivity for longer period. Report states that an inactive immunization is able to lead the immune system to regulate the disease spread till a particular immune response in the affected people. Convalescent plasma has been used in argentine hemorrhagic fever treatment, it is found that it may act against SARS-CoV-2 as. The involvement of convalescent plasma (CP) against influenza A is significant, with a meta-analysis proposed to that initial treatment, before the acute ailment develops, it could be an important efficiency interpreter of passive immunotherapy towards pathogen. Further meta-analysis of convalescent plasma examination and hyper-IgE in influenza A and SARS infected patients advised a mortality benefit “however CP is promptly focused later indication onset” [126–129].

*Itolizumab*: An anti-CD6 humanized IgG1 monoclonal antibodies, where associated to CD-6 domain is liable for priming, activation then T-cells differentiation. Since it minimizes proliferation of T-cell and considerable decrease of cytokines/chemokines production. In 2013, itolizumab recognized for prolonged plaque psoriasis. Currently, it is extended towards the studies against COVID-19 tethered cytokine storm and its complexity. Owing to COVID -19 pandemic removal throughout India, Biocon firm paid their attention towards the repurposed its novel bio applicator ALZUMAb® (Itolizumab) against COVID -19 patients. It has presented positive outcomes to cure cytokine release syndrome (CRS) for COVID -19 patients facing ARDS. Researchers have conducted the clinical trials for infected patients, and also examine the efficacy as well as its safety against COVID -19 patients along with mild to severe complicated conditions. The further studies of itolizumab also revealed the immunomodulatory effect and its association to clinical improvement, through assessing biomarker information including IL1, IL6, IL17, TNF-α, Interferon-A etc. prior and later treatment using itolizumab. A unique mode of action of immunomodulator includes CD6 receptor binding and preventing the T lymphocytes activation whereby in order overwhelms the pro-inflammatory cytokines accordingly minimizes inflammation, an extreme inflammation led to failure of organs and death. [130–132].

Propofol (ICI 35–868, *Diprivan*): It is used for sedation sometimes causes hypotension as a side effect. Furthermore, as propofol may induce an increased ACE2 expression leading to angiotensin production and subsequent vasodilation, propofol-induced hypotension may be aggravated in the patient with COVID-19. Considering the factors mentioned above, the use of propofol in patients with COVID-19 should be avoided when possible in favor of alternative sedative agents, including midazolam and dexmedetomidine. Thus, further studies regarding the risks of propofol use in the patients with COVID-19 (Fig. 9k) are needed [133, 134].

*Baricitinib* (Olumiant) with *Remdesivir* (Veklury): Baricitinib fused with remdesivir minimizes the recovery time of treatment with COVID-19 patients at NIAID-sponsored ACTT-2 trial. Moreover, we know that baricitinib belongs to a selective and reversible JAK1/JAK2 inhibitor at tyrosine protein kinase family and acts as potential role in the pro-inflammatory path indicating that is regularly over-triggered in autoimmune disorders like treatment for moderately to severely active rheumatoid arthritis, also approved in over 70 countries. On examination of baricitinib on controlled trials reveals significantly illustrated its utility, potential benefits and also understood the protection of its COVID-19 treatment. Its mode of action is JAK enzymes bind with an intracellular cytokine receptors and boost the indicating flows of cytokines and growth factors subjective in functions of haematopoiesis, inflammation and immune system in associated with RA pathogenesis. Circulation of pro-inflammatory cytokines fix to these cell surface receptors. By the combination of characteristic factors of extracellular cytokines and its development, JAKs are phosphorylated then stimulates the signal transducer and activator of transcription (STATs). By the signaling flows, inflammatory cytokine and chemokine transcription is prompted to proceed inflammatory mediators together with IL-2, IL-6, IL-12, IL-15, IL-23, IFN-γ and GM-CSF [135].

*Clofazimine* (G-30320): A typical riminophenazine dye that is extensively utilized for the treatment of multibacillary (MB) leprosy, other skin infections and act as an effective immunomodulator in the regulator of continuing ENL reactions. Together with a promising well-being profile, it has good inhibition property against various CoVs, and also have the capability to antagonize the multiplication of SARS-CoV-2 and MERS-CoV through in vitro manner. Based upon the reported data on the effective concentration influences in clofazimine play against SARS-CoV-2 through in vitro value of EC_50_ = 0.31 μM in Vero E6 cells that is clinically feasible with a single dosage of peak serum concentration (*C*_max_) of 0.86 μM at 200 mg d^−1^ and further showed EC_90_ value in different cell lines as 0.81 to 2.35 μM respectively, which is identified in plasma and lung tissue collections afterwards many dosages. Herewith we gained the report of the clofazimine defensive effect for the SARS-CoV-2 / MERS-CoV infectivity in human cell lines as well as in a hamster model [136]. *Niclosamide* (BAY-2353): An antihelminthic/molluscicide agent, which can expel parasitic worms (helminths) and various inner parasites through either striking or exploiting without affecting damage to the host. It also possess an effective antiviral behavior against ssRNA viruses containing coronaviruses that was suggested as an antiviral stuff during the SARS outburst on 2002, which makes a similar significant interest to possess antiviral activity against SARS-CoV-2, before it was focused to inhibit SARS-CoV through in vitro examination and an identical structured RNA viruses (both in vitro and in vivo). Thus the researchers imagined that the antiviral action of this drug may be extended towards COVID-19. In fact the mode of drug action led by exploiting infectious tapeworms and the other worms passed in the stool or may destructed in the intestine. Though tapeworms, even matured one are killing fast, apparently because of suspension of oxidative phosphorylation / ATPase activity inducement [137]. *ANTICOV***:** In Africa, very recently, the ANTICOV clinical trial was launched with the aim of testing the efficacy of amodiaquine, ciclesonide, ivermectin and nitazoxanide in milder and adequate COVID-19 cases. The WHO’s solidarity test was resumed with a new list of treatments targeted in alleviating the immune response. Drug combination, nitazoxanide-ciclesonide, is tested to give people with milder-to-adequate COVID-19 before the cases develop. ANTICOV represents the combination of recognized antiparasite drug nitazoxanide and the corticosteroid ciclesonide. In fact this grouping has two various mode of action able to work at many infection stages like potentially vibrant through the viral replication, minimizes the inflammatory stage, even the both drugs are commercially available [138] amongst the characteristic structure of such antiviral drug molecules and some of the monoclinical antibodies were illustrated (Fig. 9l) respectively.

## Role of drug delivery carriers for antiviral drugs

One of the important factors in antiviral drugs is bioavailability due to poor drug absorption in the gastrointestinal tract. In the antiviral acyclovir drug treatment, number of patients showed low absorption rates (only 15%). Bioavailability depends on solubility and permeability properties. Lower bioavailability requires higher dosage that may lead to toxicity. Typically the drug is administered orally, but topical and intravenous infusion techniques to improve bioavailability is also investigated. Even though the administration method is important, many viruses survive in lymph or synovial fluid system, and current antivirals are incapable to reach. So the antiviral drug has to be permeable, selective to the site and targeted drug release, to reduce the drug exposure to other organs to increase the tolerance / resistance, in particular in severe immunocompromised patients. Indeed, many antivirals have minimum half-life, so the drug administration ratio needs to be improved [139–142].

### Nanoparticles as delivery vehicles for antiviral therapeutic drugs

**Fig. 10:**
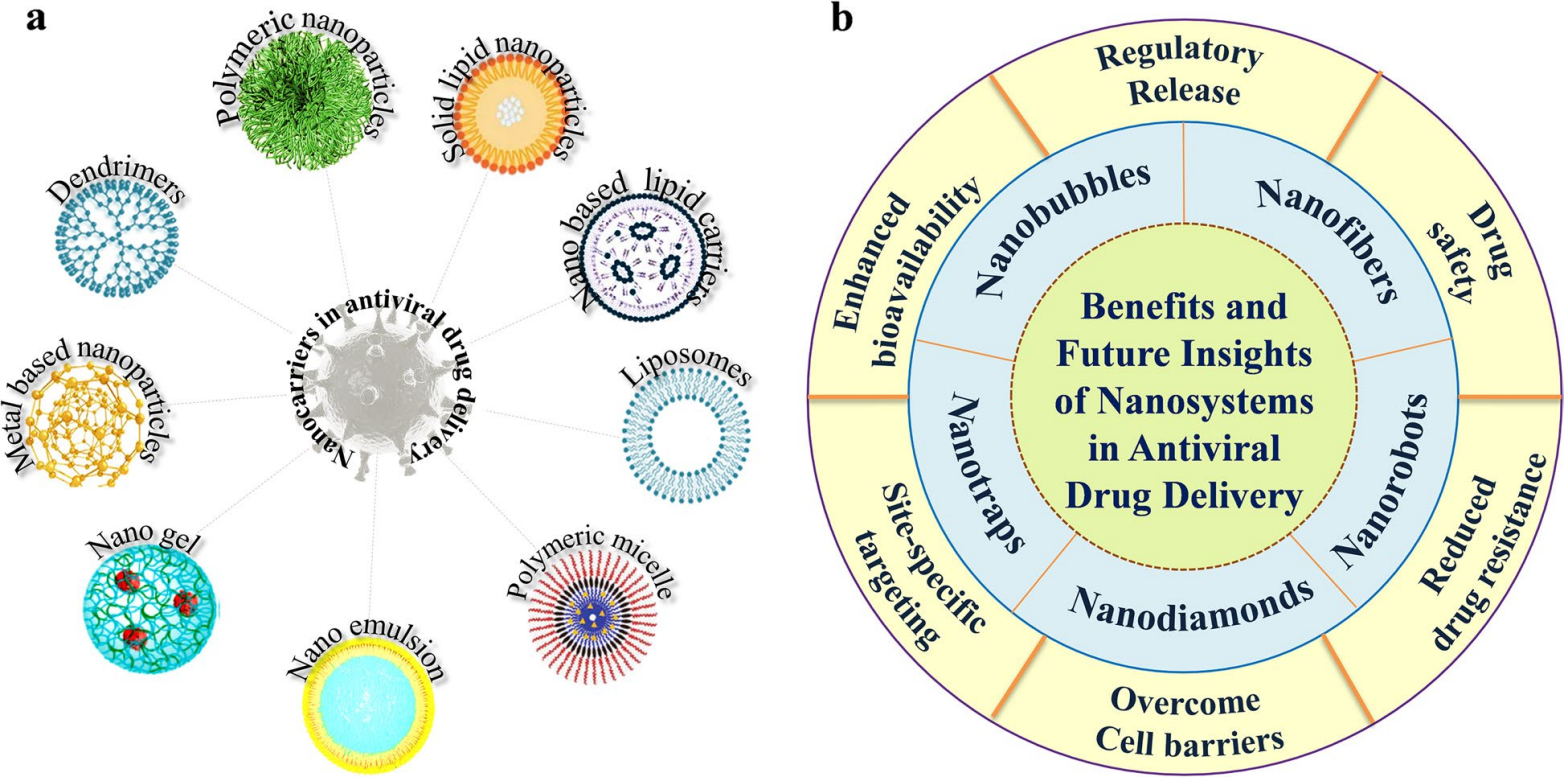
**a** Typical nanocarriers based on solid lipid-, polymeric-, dendrimers-, metal-, nanogels, nanoemulsions, polymeric micelle-, liposomes and lipid carriers nanoparticles utility in antiviral drug delivery; **b** Benefits of nano systems such as drug safety, reduced drug resistance, overcome cell barriers, site specific targeting, improved bioavailability and regulatory release and moreover future perspectives of nanosystems in drug delivery including nanofibers, nanorobots, nanodiamonds, nanotraps and nanobubbles are showed

Nano particle based antiviral drug delivery includes solid-lipid, lipid-carriers, liposomes, polymeric micelles, dendrimers, metal based, nanogels and nanoemulsions (Fig. 10a). The advantages and future ideas of nanosystems in antiviral drug delivery is also shown (Fig. 10b).

### Polymeric nanomaterials

Polymer-based nanomaterials from biodegradable and biocompatible are examined in drug delivery, due to size of the particle and prolonged blood circulation.

**Carbon based NPs**: Carbon-containing nanoparticles such as graphene, carbon dots (C-dots), fullerenes and others have confirmed as highly efficient for treatment as an antiviral drug delivery vehicles. Graphene nanoparticles are known to be active against viruses. Carbon dots (~ 10 nm) and fullerenes have been observed to affect viral activity [143].

**Cyclodextrin-based NPs**: Cyclodextrin (CD)-based materials can form hydrophobic inclusion complex in solution and solid state. The chemical properties can be modified due to the presence of many hydroxyl moieties which can be transformed with various ligands for targeting specific active sites [144].

### Micelles

They possess hydrophobic and hydrophilic characteristics with block copolymer core and corona shell respectively. They have prolonged blood circulation time, cellular selectivity, and controlled drug release. Acyclovir (ACV) drug used for the treatment against herpes, varicella zoster and Epstein-Barr viruses is delivered in the form of micelles. Hydrophilic polymers have been used as drug carriers for acyclovir to address low solubility and bioavailability, but the techniques are expensive and difficult to construct. Acyclovir tethered polycaprolactone (PCL) has been tested. Vesicles secreted by human cells, act as efficient vehicle for antiviral drug delivery. During influenza viral infections, such vesicles promptly changes into antiviral proteins and potentially inhibit viruses binding and disturbing the pulmonary cells. Owing to their innate responses against viruses, they act as promising aspirants for antiviral treatments in nanoscale proportions [145].

### Lipid carriers

They are used in drug delivery systems, particularly for messenger RNA (mRNA) therapeutics, especially for vaccines, for the treatment against cancer, protein therapy and editing of genomes. mRNA is unstable and in short supply or/and moreover this kind of antiviral therapy depends on lipid-based carriers called lipid nanoparticles (LNPs. For example, N^1^,N^1^,N^3^-tris(2-aminoethyl)benzene-1,3,5-tricarboxamides (TT) is used for mRNA delivery [146].

### Liposomes

These are lipid based nanoparticles, which contain small spherical shaped phospholipid bilayer enclosing aqueous cores (size ranges from 15–1000 nm). The aqueous shield captures the drug and delivers through active/passive targeting. Binding site of ligand/amino acid segments like antibodies, proteins / liposomes residue on the surface achieves active targeting. Liposomes, due to their bilayer arrangement easily carries huge hydrophobic / hydrophilic loads easily [147].

### Solid lipid nanoparticles

The structure of solid lipid nanoparticles (SLN) are similar to liposomes, but differs in its aggregation status. The colloidal system contains different solid lipid matrices with size range from 10 to1000 nm. Some solid lipids that make up the matrices include triglycerides, steroids, fatty acids, and waxes etc. So they may have various functionalities and abilities of target drug delivery. They have controlled drug release profile, and stability similar to liposomes [148].

### Dendrimers

They are promising agents for gene therapy. They have many branched polymers with a central moiety, successive monomers repetition as dendrons and surface clusters on the exterior. Dendrimers are spherical, and their diameter are in nanometers. For example, G4 and G5 polyamidoamines are 4 and 5 nm in diameter respectively. The core moiety is hydrophobic and charged, and attract hydrophobic drugs. Dendrimer cores can accommodate drugs for the treatment against influenza, HIV and RSV. Dendrimer carbosilane has the potential to capture drugs to treat tumors and viruses, as well as used for gene therapy [149, 150].

### Nanoemulsions

They contain water, surfactants, co-surfactants and oil existing in a single phase with encapsulated drug particles with 20 to 500 nm in diameter. Nanoemulsions increase solubility, load capability, standing time in GIT, lymph uptake, absorption and bioavailability factors. Oil containing emulsion enables absorption through gastrointestinal tract, thereby increasing the bioavailability, solubility, absorption rate, targeted drug delivery etc., which are general requirements of antivirals in fact. Owing to their dimension, capability to solubilize hydrophilic and lipophilic drugs, low toxicity, ease of penetration in epithelial layer, can solve the main problems of current antiviral drug delivery [151].

### Nanogels

The hydrogel in nanoscale aids retroviral drug delivery. A unique characteristic properties of hydrogel's 3D cross-linked polymer network is that it can swell up to 95% in water (superabsorbent) without dissolving in the aqueous media. This is made with natural, synthetic or both and exhibits solid or fluid properties. The cross-linked polymers are obtained from heterogeneous polymerization with a cross-linker with bifunctional / multifunctional elements. The representative dimension, charge density, permeability, amphiphilic, softness and degradability properties depends on nanogels [152].

### Metal based nanoparticles

Inorganic compounds show high utility in medical field from ancient times. Metal nanoparticles possesses high efficacy. For instance, gold, silver, oxides of copper, titanium, cerium, silicon etc. can treat many viruses including influenza, DENV, HBV, HCV and HIV-1. Gold nanoparticles can inhibit HIV replication, for example Raltegravir drug is associated AuNP. Similarly, silver nanoparticles (AgNPs) have antimicrobial activity, along with various properties and affect viruses. Owing to their multiple targets they possess minimal drug resistance. Silver NPs layered oseltamivir drug is used for the treatment against H1N1 virus [153–156].

## Future nanosystems for antiviral drug delivery

Providing the drug delivery system in nanoscale leads to the best outcome and enhanced properties. A few of the nano based delivery systems are discussed below.

### Nanobubbles

An oxygen-containing core of nanobubbles have echogenic characteristics that facilitates drug loading, moreover the delivery of such loaded drugs can be achieved by ultrasound technology. The nanobubble shells have lipid polymer associated with surfactant affords the stability, the best half-life, factors for prompting drug release. Besides, this nanosystems may be highly useful for theragnostic purpose (multifunctional diagnosis and treatment) [157].

### Nanofibers

Nanofibers are obtainable through electrospinning. The fibers are electro spun from polymers such as PVA (polyvinyl alcohol), chitosan, PEO (polyethylene oxide) and CA (cellulose acetate). The drugs in nanofiber may exists in pure / nanoparticulate form with distinctive properties. Studies showing controlled release of hydrophilic HIV drugs such as raltegravir and maraviroc in nano fibers have bene reported [158].

### Nanodiamonds

This is an example of harmless carbon-based nanomaterial, with stable sp3 hybridized carbon core. The surfaces are manifold and are able to coordinate with adjacent water molecules. Hence, they have the potential to dissolve water-insoluble drugs. Manufacture of these diamonds are performed at high temperature and pressure, or done by detonation process. Nanodiamonds have advantages such as physical stability, harmless, bio-availability, -compatibility, load ability, and extended spreading interval. They can be surface improved for targeted delivery and also functionalized with growth factors [159].

### Nanotraps

These nanotraps are nano-scaled homogenous hydrogels that can capture viruses, therein these are categorized by their charge density, prodigious attracting aromatic moieties surrounded by polymerized shell. Peripheral layer promote hydrophobicity and electrostatic force of interactions, in which they entices the virus, and the existing inner hydrogel preserves the virus. In the presence of different chemical and physical properties, nanotraps have been employed to capture RSV, RVFV (Rift valley fever virus), VEEV (Venezuelan equine encephalitis virus), and FMDV (Foot-and-Mouth disease virus) [160].

### Nanorobots

It is a nano-sized device composed of polymeric, inorganic and biomimetic constituents that can pass through the bloodstream. This method is suitable to provide remedy to cancer and diabetic patients, as well as address neurologic and cardiovascular disorders. In fact, nanorobots are basically miniature circuits, which have actuators, minicomputers, power source, sensors, compartments and others used to drug delivery.These robot modules can actuate, sense, signal and self-replicate, propel, to navigate body for diagnosis and /or treatment [153].

## Therapeutic applications of micro- and nano-particulates in drug delivery system

Novel drug delivery systems (NDDS), are being explored in order to maximize and minimize the therapeutic and adverse effects respectively. Despite a number of improvements in pharmaceutical formulations, one of the main still persisting task is continued drug release. Micro- and nano- particulates encapsulation passes as a smart method due to its specificity and important properties, such as biocompatible, constancy, target specific, even encapsulation, healthy compliance, and regulated and maintained release patterns are responsible for minimalizing the toxic and dose rate [161].

### Role of micro particulate drug delivery systems

Micro-particles are intended as effective delivery systems in which the encapsulation of both water-insoluble and sparingly water-soluble can be carried out to achieve unique properties such as particle dimension, shape, structural arrangement, drug load, entrapment potential, porosity, and drug release profile. To accomplish high therapeutic benefits, delivery of drug in time (with good quality) to reduce adverse effects. Micro particulate drug delivery system (MDDS) entraps solid, liquid, or gas in 1 to 1000 µm size in inert polymer shells, shielding them from the insecure external environment.

Natural polymers like gelatin and albumin and synthetic biocompatible polymers that are used include CMC (carboxyl methyl celluloses), PLA (polylactic acid), PLG (polylactide co-glycolide), PLGA (poly[lactic co-glycolic acid]), PCL (poly ε-caprolactone), and polyketals. Molecular weight, bio-compatibility, degradability, and nature of the polymer determines the drug encapsulation efficiency [162, 163].

### Polysaccharide NPs for treatment of lung fibrosis

Polysaccharides are more superior to other polymers because of their characteristic ranges in size, charge density, biodegradability, available in abundance, biocompatible, and minimal toxicity. Polysaccharides can be functionalized along with nanoparticle- conjugates or therapeutic units including amino acid cluster, peptide molecule, nucleic acids and even small molecular skeletons. They also exhibit good pharmacokinetic and drug delivery properties, which stimulates oral absorption, drug release, in vivo retention ability, and exert synergistic effects. Polysaccharides based NPs prepared with cellulose, chitosan, hyaluronic acid, alginate, dextran, starch, cyclodextrins, pullulan and their combinations exhibit promising activities in organ fibrosis, diabetes and arthritis. Problems with potential drug delivery is the inability to cross the cell membranes. Meantime the plasma membrane actively participated as a boundary between the cells and exert cellular response that is essential for cellular function. Even this membrane blocks the specific therapeutic agent entry into the cellular organelle. Novel drug delivery systems (NDDS) hold potential for target oriented delivery of gene, drug, diagnostic and theranostic agent to specific cells or intracellular organelles and release the contents in a meticulous manner within a specified time. The polymeric NPs with size ranging from 1 to 1000 nm show ultrafine characteristics of colloidal particles. They also exhibit improved- binding ability, reactivity and therapeutic potential when compared to other delivery methods. They are capable of increasing the drugs concentration and intracellular reservoir in prolonged release. These can be rapidly internalized and degraded, and hence facilitate the drugs release / conjugates in the intracellular space.

Treatment of lung fibrosis in the neoteric period, is a critical task. Traditional techniques have led to concern for both local and systemic applications. It includes many contractions like pharmacokinetic instability, drug resistance and low therapeutic index. Recently pulmonary delivery of NPs with therapeutics has been studied with polysaccharide nanocarriers and they have been evidenced as promising applicant to eradicate the barriers of typical drug therapy thereby resulting in lower pulmonary toxic character. NPs interact non-covalently to the drug or by encapsulating and assisting in targeted drug delivery. To preclude adverse effects as pulmonary embolism, submicron particles are administered intravenously. Lungs have higher vascularization with greater surface region of alveolar epithelium that act favorably for drug absorption. Due to presence of lesser toxicity, versatility and biocompatibility, they have been significantly utilized in clinical therapeutics of various disorders. Drug delivery through the pulmonary system is connected with many benefits as compared with other conventional administration routes due to its higher surface area, permeability, vascularization and insignificant viscosity of the blood– alveolar barrier. Drug delivery through pulmonary is favored to treat against respiratory diseases, like chronic obstructive pulmonary disease, pneumonia, TB, and cystic fibrosis. Despite the exceptional properties of the drug delivery via the pulmonary system, with rapid onset of action, local drug delivery, minimization of dose, reduced side effects, and release during metabolism, a necessity is to enhance controlled drug release to evade regular administration, to maintain a drug constant level, and to enhance patient ease and amenability. Other aspects include prevent macrophage uptake, mucociliary clearance, and degradation by metabolic enzymes. Generally particles greater than 6 µm undergo mucociliary clearance and ~ 1–3 µm undergo phagocytosis by macrophages. Sometimes cytochrome P450 enzymes present in the lungs leads to degradation of the drug and hence its inactivation. Though, size is one of the vital factors to determine the drug efficacy in pulmonary delivery, micro particles delivered through the pulmonary system may emerge as an interesting strategy in the future [164–169].

### Role of SeNPs in inflammatory diseases

Among the various NPs, metals like gold, silver, cerium, iron, selenium titanium and zinc have occupied a distinct role due to their unique bioactivities in nano-forms. Selenium (Se) is one of the essential trace elements, which is combined into protein by cysteine as selenocysteine (Sec) exhibiting enzymatic activity. Many selenium based proteins possess oxidoreductase activity, which controls the physiological redox balance. Since selenium nanoparticles (SeNPs) exhibit a low toxicity is explored in various oxidative stress and inflammation disorders such as arthritis, cancer, diabetes and nephropathy for therapeutic benefits. SeNPs associated with Ulva lactuca polysaccharide enhances stability. Use of dextran sulfate sodium (DSS) with this NP can cure colitis by inhibiting the pro-inflammatory cytokines (IL-6 and TNF-α) and NFκB signaling pathway. Natural polysaccharides show improved bio-availability, compatibility and low toxicity. The polysaccharides containing hydroxyl motifs inhibit the NPs aggregation through intermolecular hydrogen bonding. The anti-inflammatory activity of polysaccharide based SeNPs is through the inhibition of NF-κB path via inhibitory protein Iκ-B subunits and inhibit the phosphorylation of JNK1/2, p38 MAPKs. Similarly, melatonin-SeNPs increases the activity of antioxidant enzymes like SOD and GPX, decreases serum ALT, AST, NO, MDA levels, liver pathological abnormalities, pro-inflammatory cytokines and splenocyte proliferation. SeNPs oral treatment improves the pro-inflammatory cytokines including Th 1, cytokines IFN-γ, IL-2, IL-12 and TNF-α and also increases the delayed hypersensitivity reaction [170–174].

## Ongoing status for clinical development against COVID-19

Emergence of SARS-CoV-2 caused universal jeopardy and destroyed millions of human lives. The economic crisis has affected millions. A number of vaccines and drugs are currently practiced in several countries. Several drug and vaccines are still in clinical stage and hence being tested in large scale (Table 2). Currently, different types of viral treatments are under investigations such a*s single agents* and in *combinations* for COVID-19. Accordingly the single agent *antivirals* (50 +), *cell and gene therapies* (60 +), *immunomodulators* (120 +), neutralizing antibodies (60 +), others (110 +), and combinations (40 +) were under examination. Furthermore, 690 + number of drug development program are under progress; 470 + number of clinical trials reviewed by FDA; 15 + number of drugs authorized for emergency purpose; currently one (1) drug is approved by FDA [175].

**Table 2:** Range of antiviral drugs against COVID-19 under clinical trials

Candidate Drugs	Groups	Mode of action	Activity	NCT number/Recruitment Status/ latest update posted/ Phase/ Sponsor/ Estimated enrollment
**Remdesivir (GS-5734)** **DB14761** **Gilead Science**	Adenosine triphosphate analogues Antiviral drug Ebola drug	RNA polymerase inhibitors, block RNA polymerase that incorporates in to nascent viral RNA chain- and premature termination	EC_50_ = 0.77 μM in vero E6 cells, CC_50_ > 100 μM, SI > 129.87 load 200 mg dosage on day 1 is drawn, followed by 100 mg iv once-daily maintenance doses for 9 d	NCT04252664 (**Suspended**) (owing to the COVID-19 pandemicity, it has been controlled at present, no eligible patients can be recruited) April 15, 2020 Phase 3 Capital Medical University 308 participants NCT04257656 (**Terminated**) (The spread of COVID-19 has been controlled well in China, no eligible patients can be enrolled at present.) April 15, 2020 Phase 3 Capital Medical University 237 participants NCT04292730 (**Completed**) January 26, 2021 Phase 3 Gilead Sciences participants 14 countries
**Hydroxychloroquine** **Plaquenil** **HCQS** **DB01611**	Aminoquinoline Antimalarial and Autoimmune disease or immune-modulating activity drug Anti-rheumatoid arthritis drug	Block viral infection by upsurging endosomal pH needed for virus cell fusion along with interfering the glycosylation of cellular receptors (ACE2) for SARS-CoV-2 inhibits heme polymerase action	EC_50_ = 1.13 μM CC_50_ > 100 μM SI > 88.50 Prevent orf1ab, orf3a, orf10 to combat the heme to form the phorphyrin and inhibit orf8 and surface glycoprotein to porphyrins 400 mg (administered as two 200 mg capsules) orally twice for 2 dosages daily	NCT04358068 (**Terminated**) (Slow enrollment and lack of community enthusiasm November 16, 2021 Phase 2 National Institute of Allergy and Infectious Diseases (NIAID) 20 participants NCT04303507 (**Active**, **not recruiting**) February 4, 2022 University of Oxford 40,000 participants
**Ivermectin-(MK-933)** **Stromectol, Sklice™** **Abbott** **DB00602**	Macroloids (Pentacyclic sixteen membered lactone disaccharide). Milbemycin type antihelmintics, antiparasitic Onchocerciasis (River blindness) against tapworms	Inhibiting IN Nuclear ingress and HIV-1 replication. Nuclear transport inhibitory activity	~ 5000 fold reduction in viral RNA at 48 h	NCT04343092 (**Completed**) November 4, 2020 Phase 1 University of Baghdad 16 participants NCT04390022 (**Completed**) December 17, 2020 Phase 2 Clinica Universidad de Navarra 24 participants
**Favipiravir (Favilavir)** **(T-705)** **Fujifilm’s or Avigan** **DB12466**	Pyrazine or Quanine analogues Anti-influenza, Drug against Ebola, Lassa, Yellow fever, Chikungunya, Noro and Entero-viruses	RdRp inhibitors hinders the flavi-, alpha-, filo- bunya-, arena-, noro and other viruses replication	EC_50_ = 61.88 μM in vero E6 cells, Inhibit the “E” and ORF7a proteins associated to porphyrin prevent viral entry from host cells and catch free porphyrins	NCT04351295 (**Completed**) November 1, 2021 Phase 2 Tanta University 92 participants
**Lopinavir-Ritonavir** **ABT-378/538** **Kaletra/Norvir** **DB01601/DB00503**	Peptidomimetic molecule, Antiviral drug, HIV-1 drug Antiretroviral protease inhibitors, CYP3A inhibitors	HIV-1 protease inhibitors-3- chymotrypsin (3CL)-like and paplin (PL-like protease) of 2019-nCOV	EC_50_ of lopinavir is about 17 µM against SARS-CoV	NCT04321174 (**Active**, **not recruiting**) December 14, 2021 Phase 3 Unity Health Toronto 123 participants
**Azithromycin** **(XZ-450)** **Zithromax** **DB00207**	Macrolide, antibiotic, anti-bacterial infection drug	HY/AZ a promising influence on clinical results and viral loads on the infected patients	Five out of nine patients with clinical manifestation of severe QTc prolongation had a normal QTc at baseline	NCT04358068 (**Terminated**) (Slow enrollment and lack of community enthusiasm) November 16, 2021 Phase 2 National Institute of Allergy and Infectious Diseases (NIAID) 20 participants NCT04332107 (**Terminated**) (**Futility**) October 7, 2021 Phase 3 Thomas M. Lietman, University of California, San Francisco
**Ribavirin** **(SCH-18908)** **Ribapak, Copegus** **DB00811**	Trizole / Guanosine nucleoside antiviral drug hepatitis C viral hemorrhagic fevers, RSV infection	Inhibits the synthesis of viral mRNA	The EC_50_ of ribavirin against SARS-CoV-2 was 109.50 µM, CC_50_ > 400 μM, SI > 3.65	NCT04392427 (**Not yet recruiting**) September 3, 2020 Phase 3 Mansoura University 100 participants
**Interferon beta-1b, Lopinavir–Ritonavir (keletra) and Ribavirin Combinational therapy**	MS drug, HIV drug antiviral drug	Triple antiviral therapy superior to L-R only in shortening virus shedding, relieving symptoms and assisting discharge of patients with mild to moderate COVID-19	EC_50_ of lopinavir is 17 μM EC_50_ of interferon beta-1 is 0.12 IU/mL EC_50_ of ribavirin is 109 μM Hazard ratio 4.37 [95% CI 1.86–10.24] *p* = 0.0010	NCT04276688 (**Completed**) April 15, 2020 The University of Hong Kong Phase 2 127 participants
**Galidesivir** **BCX4430** **GS-6620**	Adenosine analogAnti-viral drug, Yellow fever, Hepatitis C, Ebolavirus, Marburg virus, Zika virus	Nucleoside RNA polymerase inhibitors, RdRp inhibitors	CC = 500 μM	NCT03891420 (**Terminated**) (Sponsor decision to no longer pursue indications studied in this trial) Phase 1 May 27, 2021 BioCryst Pharmaceuticals National Institute of Allergy and Infectious Diseases (NIAID) 24 participants
**Nafamostat mesylate (INN)** **(Fusan)** **Berabu** **DB12598** **FUT-175**	A member of guanidine or benzoic acid analog Anti-viral, Anti-cancer Anti-coagulant	Inhibitor of SARS-CoV-2 spike protein membrane fusion Potent serine protease TMPRSS2 inhibitors of SARS-CoV-2	EC_50_ = 22.50 μM CC_50_ > 100 μM, SI > 4.44 EC_50_ = 5 nM	NCT04352400 **Recruiting** Phase 2 Phase 3 University Hospital Padova Yokohama City University, University of Zurich 256 participants NCT04418128 (**Not yet recruiting**) Phase 2 Phase 3 June 9, 2020 Gyeongsang National University Hospital 84 participants
**Imatinib mesilate (INN)** **Gleevec, celonib** **DB00619** **STI 571**	Benzamide methanesulfonateAnti-cancer, Anticolonal activity, BCR-ABL kinase inhibitor and block the entry option	Potent serine protease TMPRSS2 inhibitors	Reduced mortality from 100% to 30–35%, EC_50_ = 87 nM	NCT04394416 (**Recruiting**) Phase 3 August 12, 2021 University of Maryland, Baltimore 204 participants NCT04357613 (**Not yet recruiting**) August 7, 2020 Phase 2 Versailles Hospital 99 participants
**Comostat mesylate** **(Foypan)** **DBSALT002914** **FOY- 305**	Benzene acetic acid oxo ethyl ester Kinase inhibitor Serine protease inhibitors that blocks TMPRSS-2 mediated cell entry of SARS CoV-2	Inhibitor of SARS-CoV-2 spike protein membrane fusion SARS-CoV-2 Celluar entry blocked	Nafamostat inhibiting viral entry with potential efficacy of approximately 15-fold greater than that of camostat	NCT04321096 (**Recruiting**) April 30, 2021 Phase 1 Phase 2 University of Aarhus 580 participants
**Nitazoxanide (NTZ)** **DB00507** **PH-5776** **Alpex Nizox**	Thizolides class drug, an antiprotozonal, antiviral agents Diarrhoea treatment Nitazoxanide is based on a thiazolide for anti-infective with potential efficacy in parasitic, bacterial and viral infections	During viral infectivity like MERS-CoV infection, it acts by prevent maturation of viral protein of nucleocapsid-N- that supports viral particles formation	EC_50_ = 2.12 μM, CC_50_ > 35.53 μM, SI > 16.76 (EC_50_ = 2.12 μM in Vero E6 cells)500 MG/6 h/6d	NCT04341493 (**Terminated**) (Concerns about safety of Hydroxychloroquine) April 1, 2021 Phase 4 Hugo Mendieta Zeron 44 participants
**Penciclovir (Denavir)** DB00299 BRL-39123	Nucleoside analoguesAcyclic guanine derivatives, Antiviral activity, Herpesvirus (HSV) infection	Preventing growth of virus Polymerase inhibitor	EC_50_ = 95.96 µM, CC50 > 400 μM, SI > 4.17	No satisfactory response at in vitro SARS-CoV-2 model (**Under investigation**)
**Darunavir ethanolate** **Prezista** **33078XF0BW** **TMC-114** **Darunavir/Cobicistat**	Anti-HIV/AIDS drug, Antiretrovirals	Second generation of HIV-1 protease inhibitor- inhibited SARS-CoV-2 infection through in vitro	Phase 3 (800 mg/150 mg) 1 tablet PO once daily	NCT04252274 Unknown (**Recruiting**) April 13, 2020 Phase 3 Shanghai Public Health Clinical Center 30 participants NCT04425382 Observational (**Recruiting**) June 11, 2020 200 participants
**Arbidol (ARB)** **Umifenovir** **DB13609** **J05AX13**	Small Indole prophylaxis based dual acting direct antiviral /host targeting agent; Anti-influenza virus A and B drug and respiratory infection	Inhibit SARS-CoV-2 infection 10–30 μM by in vitro	10–30 μM in *vitro*	NCT04350684 (**Unknown**) Enrolling by invitation Phase 4 Shahid Beheshti University of Medical Sciences 40 participants
**Interferon alpha (IFN-α)(Roferon-A®)** **DB05258** **Interferon beta-1b (IFN-β1)** **DB00068** **andInterferon family**	Protein Based Therapies, Immune response against virus, Anti-heptatitis virus drug, Leukemia (lymphoid) Leukemia (myeloid) MS drug	Inhibit the SARS-CoV reproduction through in vitro	IFNβ1 may directed to a safe and easy in the early stages of infection against COVID-19, also utilized in alone or in combination with other drugs led to the treatment for severe patients with COVID-19	NCT04379518 (**Recruiting**) March 2, 2022 Phase 1 Phase 2 Roswell Park Cancer Institute 64 participants
**Disulfiram** **Antabuse** **Dupont 4472** **DB00822**	Carbamate drug to treat alcohol dependence or deterrent respiratory diffulties, inhibiting aldehyde dehydrogenase	Inhibiting the PL^pro^ of MERS and SARS in cell culture. Potentially active against M.^pro^, IC_50_ = 9.35 ± 0.18 μM	Inhibiting cystokine storms in COVID-19 FDA-approved disulfiram inhibits pyroptosis by blocking gasdermin D core formation	NCT04594343 (**Completed**) October 8, 2021 Phase 2 Spring Research Foundation 140 participants
**Ebselen** **DB12610** **DR-3305**	Organoselenium drug, anti-inflamatory, anti-oxidant, Glutothione peroxidase activity, Cytoprotective activity, Meniere’s disease, Type 1&2 Diabetes mellitus	Penetrate cellular membrane to access their target IC_50_ = 0.67 ± 0.09 μM	EC-50 values of 4.67 ± 0.80 μM, Ebselen exhibit low cytotoxicity (LD_50_ in rats 4,600 mg/kg, per os) and its protection for humans evaluated in many clinical trials. These data strongly recommend for the clinical test of ebselen for CoV treatment	NCT04485130 (**Recruiting**) May 10, 2021 Phase 2 University of California, San Francisco 60 participants
**Famotidine** **Pepcid** **DB00927**	Anti-viral Peptic or gastric ulcer disease Histamine-2 receptor antagonist	PL.^pro^ is encoded by the SARS-CoV-2 genome and essential to the viral entry of SARS-CoV	Histamine-2 receptor antagonist famotidine is a putative therapy for COVID-19.This high-dose oral famotidine is well tolerated and associated with improved patient-reported outcomes in non-hospitalised patients with COVID-19	NCT04389567 (**Completed**) June 4, 2020 Observational North well Health Cold Spring Harbor Laboratory 10 participants
**Tocilizumab (TCZ)** **Atlizumab or Actemra DB06273** **MSB-11456** **Sarilumab-(Kevera) DB11767**	Protein based thrapies Inflammatory cytokine IL-6. Monoclonal-antibody (mAb) antagonists of the IL-6 receptors are normally for treat Rheumatoid arthritis	IL-6, a pro-inflammatory cytokine created through cells containing T-cells, B-cells, lymphocytes, monocytes, fibroblasts. Indeed, they activates C-reactive protein, serum amyloid A, fibrinogen, haptoglobin, and α-1-antichymotrypsin, however inhibiting the fibronectin, albumin, transferrin, antibody productions, cytotoxic T-cell differentiation, and inhibits regulatory T-cell differentiation. Finally the tocilizumab impasses soluble and membrane contains IL-6 receptors, inhibit IL-6 facilitated inflammation	The CT results of a person gained a treatment against COVID-19 using tocilizumab drug of IL-6, associated with the overactive inflammatory response in the lungs of severe/critical affecting COVID-19 patients	NCT04363853 (**Active**, **not recruiting**) November 30, 2020 Phase 2 Instituto Nacional de Cancerologia de Mexico 200 participants NCT04372186 (**Active**, **not recruiting**) September 27, 2021 Phase 3 Genentech, Inc 377 participants NCT04346355 (**Terminated**) (Based on interim analysis for futility and given an enrolment rate almost nil) June 22, 2020 Phase 2 Azienda Unità Sanitaria Local Reggio Emilia 126 participants NCT04320615 (**Completed**) June 30, 2021 Phase 3 Hoffmann-La Roche 452 participants NCT04327388 (**Completed**) March 17, 2022 Phase 3 Sanofi Regeneron Pharmaceuticals 420 participants NCT04357860 (**Completed**) July 7, 2021 Phase 2 Maimónides Biomedical Research Institute of Córdoba 120 participants NCT04317092 (**Active**, **not recruiting**) March 3, 2021 Phase-2 National Cancer Institute, Naples, 402 participants
**Sotrovimab (VIR-7831) GSK4182136** (**GlaxoSmithKline LLC)**	Sotrovimab, it is based on monoclonal antibody used for treatment against COVID-19 patients in a milder to moderate stage at more risk for demise / clinical treatment	Sotrovimab, it is a recombinant human IgG1k monoclonal antibody acts by interacting with a preserved epitope present on the spike protein receptor-binding domain of SARS-CoV-2. Since this conserved epitope depressing the improvement of viral resistance to the antibody. Thus impedes the spike protein facilitated interaction of SARS-CoV-2 and their accessibility into human cells. Sotrovimab never participate with human ACE2 receptor binding and inhibits an unknown process, which ensues later viral addition and previous viral and cell membranes fusion. The Fc sotrovimab constituent contains amino acid of M428L / N434S (LS mutant) products increase the antibody half-life	On account of the FDA approved GSK and Vir biotech company emergencies used the authorization for sotrovimab in the treatment of COVID-19 based on interim outcomes from a clinical test representing that practice with sotrovimab resulting 85% decreased dangerous factors of demise / hospitalization with COVID-19 patients at high risk	NCT04545060 (**Completed**) September 27, 2021 Phase 2 Phase 3 Vir Biotechnology, Inc Glaxo SmithKline 1057 participants
**Propofol** **ICI 35–868, (Diprivan)** **Sevoflurane**	Generally, Propofol (2,6-diisopropylphenol) is a sedative-hypnotic component which is useful for both induction as well as maintaining of such sedative action during critical care conditions	The propofol’s systematic action implicates a positive variation on the characteristic inhibiting activities of the neurotransmitter gama-aminobutyric acid (GABA) via GABA-A receptors	Propofol conceivably utilized to be the prior problem-solving processes on needful anaesthesia during treatment of refractory position epilepticus, then for induction and/or sustaining anaesthesia before and throughout surgeries. Regaining from propofol based anaesthesia is usually faster and associated with lesser side effects (drowsy, nausea like factors), than combined with etomidate, methohexital, and thiopental	NCT04359862 (**Terminated**) (Low recruitment ratio) July 27, 2021 Fundación para la Investigación del Hospital Clínico de Valencia 19 participants NCT04355962 (**Completed**) July 19, 2021 Phase 3 University of Zurich 68 participants
**Bamlanivimab**	Monoclonal antibodies are laboratory-made proteins that mimic the immune system’s ability to fight off harmful antigens such as viruses. Bamlanivimab is a mAb that is specifically directed against the spike protein of SARS-CoV-2, designed to block the virus’ attachment and entry into human cells	It is a recombinant human IgG1k monoclonal antibody which can neutralizing and functioning against the spike protein after screening the recovering patient’s antigen-specific B-cells. X-ray crystallography and cryo-EM structural determination suggest that bamlanivimab binds the receptor-binding domain (RBD) of the S protein at a position overlapping the ACE2 binding site and which is accessible in both the up and down conformations of the RBD.1 Specifically, bamlanivimab binds to the S protein with a KD of 0.071 nM and blocks S protein-ACE2 interactions with an IC50 value of 0.025 μg/mL.7		November 09, 2020 Today, the U.S. Food and Drug Administration issued an emergency use authorization (EUA) for the investigational monoclonal antibody therapy, Bamlanivimab for the treatment of mild-to-moderate COVID-19 in adult and pediatric patients
**Fluvoxamine Maleate** **(Luvox)** **DB00176**	Anti-depressant Obsessive –compulsive disorder	Selective serotonin-reuptake inhibitor (SSRI) binds to σ-1 receptor to shut down the inflammatory cascade from the endoplasmic reticulum in cells	Fluvoxamine may prevent sepsis a deadly inflammatory disease characterized by overactive immunue response. Decrease the generation of cytokines300 mg perday	NCT04342663 (**Completed**) July 9, 2021 Phase 2 Washington University School of Medicine 152 participants
**Thalidomide** **DB01041** **NSC-66847**	Piperidinyl isoindole Non-barbiturate hypnotic Immunono suppressive and anti-angiogenic, anti-inflammatory activities	Degradation of mRNA in blood cells and reduce the tumor necrosis factor-α and increase the secretion of interleukins such as IL-12 and activate natural cell killers	Thalidomide (100 mg) will be used for two weeks to treat lung inflammation in 100 participants with COVID-19	NCT04273581 (**Unknown**) (**Not yet recruiting**) February 21, 2020 First Affiliated Hospital of Wenzhou Medical University Phase 2 40 participants NCT04273529 (**Unknown**) (**Not yet recruiting**) Phase 2 Verified February 2020 by First Affiliated Hospital of Wenzhou Medical University First Affiliated Hospital of Wenzhou Medical University 100 participants
**Glucocorticoid** **DBCAY0002235** **Calcort, Deflazacort**	Steroids hormone Treat asthma, inflammatory pruritic dermatoses, nonallergic rhinitis	Potent inflammatory inhibitors, Glucocorticoids inhibit many inflammation related molecules as cytokines, chemokines, and arachidonic acid, adhesion molecules Binding of glucocorticoid receptor complex to GC responsive elements in promoters region of the genes	Glucocorticoid therapy may increase the risk of death in patients with nCoV-2 infections with mild symptoms. Clinical trials with low dose systemic glucocorticoid therapy may be acceptable for a short duration	NCT04244591 (**Completed**) June 16, 2020 Phase 2 Phase 3 Peking Union Medical College Hospital 80 participants NCT04273321 (**Completed**) May 11, 2020 Beijing Chao Yang Hospital Phase 3 86 participants
**Methylprednisolone (MP) acetate** **Dopo-Medrol** **DBSALT001157** **NSC-19987**	Prednisolone derivative, anti-inflammatory, immunomodulating properties, suppress the immune system and reduce the inflammation, Skin diseases, rheumatic disorders,MS, TB and COPD	The growth of diseases associated with the proinflammatory host response prompted us to evaluate the role of early corticosteroid therapy in COVID-19 patients with moderate to severe affect	Early, low-dose and short-term application of methylprednisolone associated with better clinical outcomes in severe COVID-19 patients, and should be considered before the existence of ARDS methylprednisolone treatment at an approximate 80 mg dose	NCT04323592 (**Completed**) June 24, 2020 Observational University of Trieste 173 participants
**Dexamethasone Flurorinated corticostreoid DB01234 or MK-125 Dextenza Dectancyl, Ozurdex Steroid drug Dexamethasone emerges as a life-saving drug**	Steroid drug Anti-inflamamatory,anti-allergic, skin disease asthma Rheumatic Arthritis chronic obstructive lung disease, eye pain	Reduce inflammation at various conditions including its disorders	Dexamethasone, the first drug used to reduce mortality from COVID-19. During treatment shown the reduced deaths in severely affected CoV patients by WHO report, The benefit has only seen in patients seriously ill with COVID-19, and was not observed in patients with milder disease, WHO prescribed 6 mg once per day 10 days	NCT04327401 (**Terminated**) Phase 3 (The Data Monitoring Committee recommended to stop the trial based on the Recovery Trial results, which was accepted by the CoDEX Steering Committee.) Hospital Sirio-Libanes August 7, 2020
**N3 inhbitors** **Michael acceptor inhibitor (N3)**	Peptide based drug	N3, a potent irreversible inhibitor of COVID-19 viral M.^pro^	EC_50_ = 16.77 ± 1.77 μM CC50 > 133 μM	*
**Thiadiazolidinone-8 (TDZD)** **Glitazones** **DBCAT002198** **Tideglusib** **DB12129** **NP-031112**	Thiazoles heterocyclic compounds Type 2 diabetes Alzheimer's disease	Tideglusib is a potent inhibitor of COVID-19 virus M.^pro^ Irreversible GSK-3 inhibitor with IC_50_ s of 5 nM	IC_50_ = 1.55 ± 0.30 μM	NCT04317092 (**Active**, **not recruiting**) March 3, 2021 Phase 2 National Cancer Institute, Naples 402 participants
**Sofosuvir** **Sovaldi** **DB08934** **GI-7977** **GS-7977**	Nucleotide analog, anti-viral, triphosphate (HCV) polymerase Inhibitor of Hepatitis Cvirus	Sofosbuvir may be a potential inhibitor of COVID-19 due to the resemblance between the replication mechanisms of the HCV and the coronavirus	Sofosbuvir (SOF)	NCT04773756 (**Completed**) February 26, 2021 Phase 4 Alexandria University 54 participants
**Plitidepsin** **Dehydrodidemnin B** **Aplidin** **NSC638719**	Anticancer drug Didemnins class of compounds Small molecule, Peptide Pancreatic, stomach, bladder and prostate cancers drug	Plitidepsin usage led to specific decrease in subgenomic RNA expression	Plitidepsin inhibited SARS-CoV-2 replication with an IC_90_ of 3.14 nm and selectivity index of 40.4 suggests they have potent antiviral activity in primary Human lung cells	NCT04784559 (**Recruiting**) April 1, 2022 Phase 3 PharmaMar 609 participants
**Clofazimine** **Lamprene** **NSC-141046**	Clofazimine is indicated for the treatment of lepromatous leprosy Anti-microbial riminophenazine dye	Clofazimine decrease SARS-CoV-2 infection at a post-entry step as shown by the observed reduction of viral infection when clofazimine was added at 5hpi, Clofazimine inhibited the spike mediated cell fusion activity and also inhibits post-entry steps of viral replication	Clofazimine reduces MERS-CoV replication in Vero E6 cells with an EC_50_ of 1.48 ± μM; The effective concentration of clofazimine against SARS-CoV-2 in vitro (EC_50_ 0.31 μM in Vero E6 cells) is clinically achievable with a single dose of 200 mg/day per man (C_max_ 0.86 μM).(EC_90_ 0.81–2.35 μM)	NCT04465695 (**Recruiting**) July 15, 2020 The University of Hong Kong Phase 2 81 participants
**Bepridil** **CERM-1978**	Tertiary amine Anti-arrhythmia drug Vasodilator agent Antihypertensive agent Calcium channel blocker	Bepridil exhibited strong inhibitory effect of SARS-CoV-2 from entry and replication in Vero E6 and A549 cells at a low molar micromolar concentration	IC_50_ value for bepridil (72 μM) is comparatively high, the better indicator of the efficiency of an antiviral candidate, EC_50_ values to be 0.86 and 0.46 μM in Vero E6 and A549/ACE2 cells respectively	^a^Bepridil is potent against SARS-CoV-2 in vitro
**Brilacidin** **(PMX-30063)**	Synthetic, non-peptide, small molecule mimetic of HDPs (Host Defence Protein) Magainin	Brilacidin main mechanism appears to distrupt viral integrity and impact viral entry	Brilacidin efficiently inhibits SARS-CoV-2 in an ACE2 positive human lung cell line It achieved high selectivity index of 426 (CC_50_ = 241 μM/IC_50_ = 0.565 μM)	NCT04784897 (**Completed**) August 13, 2021 Phase 2 Innovation Pharmaceuticals, Inc 120 participants
**Opaganib** **Yeliva** **ABC294640**	Anti-inflammatory, anticancer and anti-viral activity Chlorobenzenes, benzenoids, organic oxides, Azacycle, Aromatic heteropolycyclic compounds Aryladamantane	Sphingosine kinase- (SK2) inhibitor in COVID-19 Pneumonia By inhibiting SK2 the drug potentially blocks viral replication and pathological inflammation	Preliminary results from the patients have shown an improved C-Reactive Protein (CRP) an inflammatory biomarkers, with four of the five patients also provides measurable clinical improvements of reduced oxygenation and high lymphocyte counts in days treatment initiation of drug	NCT04467840 (**Completed**) July 20, 2021 Phase 2 Phase 3 Red Hill Biopharma Limited 475 participants
**Nelfinavir (NFV) (Viracept)** **Molnupiravir MK-4482 (EIDD-2801) and convalescent serum** **AG1343**	Antiretroviral protease inhibitor, Aryl sulfide, organic hetero-bicyclic compound, Benzamide members of phenols isopropylester, N4-hydroxycytidine	Inhibit the viral replication	Nelfinavir (NFV) (Viracept) Molnupiravir (EIDD-2801) and convalescent serum 614 resulting better efficacy and minimum toxicity for the treatment of SARS-CoV-2 than drugs alone against SARS-CoV-2 infection in lung epithelial A549 cells	This trial was registered in Japan Registry of Clinical Trials (jRCT) (jRCT2071200023) on July 21, 2020 NCT04575597 (**Active, not recruiting**) November 4, 2021 Phase 2 Phase 3 Merck Sharp & Dohme Corp 1850 participants
**Methotrexate (MTX)** **Amethopterin** **Teexall, Otrexup, Rasuvo** **BDSALT000115**	FDA approved Inhibitor of purine biosynthesis Chemotherphy agent, Immune system suppressant Anti-rheumatic drug Antimetabolite	By inhibiting the host-encoded pathway, which provided ribinucleotides to viral RNA synthesis. Drug methaotrexate efficiently blocks SARS-CoV-2 replication at post-entry stages of the SARS-CoV-2 infection Viral release	MTX severely inhibits the accumulation of cellular ATP	NCT04352465 (**Unknown**) (**Not yet recruiting**) April 29, 2020 Phase 1 Phase 2 Azidus Brazil 42 participants
**Pluristem** **PLX cells** **Pluristem, their placenta-derived so-called PLX cells**	Allogeneic mesenchymal-like cells have immunomodulatory properties, Immunomodulatory properties	Trigger the immunue system’s Natural regulatory T- cells and M2 macrophages natural killer (NK) cells can destroy cancer and by extension also virus-infected cells of CoV patients	PLX cells showed therapeutic benefit in pulmonary hypertension, lung fibrosis, actute kidney, and gastrointestinal injuries	NCT04389450 (**Active**, **not recruiting**) January 11, 2022 Phase 2 Pluristem Ltd 66 participants
**Blood Plasma therapy for COVID-19**	Antibodies are found in part of the blood called plasma. Plasma from blood donated from recovered patients, which contains COVID-19 antibodies	Convalescent plasma is a promising treatment that could help patients whose bodies aren’t producing enough antibodies to curb the disease. This trial will help us understand whether the treatment should be used widely to treat COVID-19	Convalescent plasma therapy involves transfusing certain components from the blood who have recovered from a viral attack into people who are very sick with the virus or at high risk	NCT04356534 (**Completed**) October 26, 2021 Phase 3 Royal College of Surgeons in Ireland—Medical University of Bahrain, 40 participants NCT04372979 (**Terminated**) (Reluctance of patients and physicians in this transfusion study setting. Blood products have expired. New variants have appeared since the plasma collection period. Some recent publications question the effectiveness of transfusion) April 14, 2022 Phase 3 Direction Centrale du Service de Santé des Armées University Hospital, Grenobl 18 participants

^a^Some of the drugs/inhibitors are to be included or excluded; National Clinical Trial (NCT) number from https://Clinical Trials.gov.https://clinicaltrials.gov. - ClinicalTrials.gov is a database of privately and publicly funded clinical studies conducted globally; ClinicalTrials.gov is a resource provided by the U.S. National Library of Medicine; Clinical trials: Phase 4: Arbidol, Sofosuvir, Nitrozoxanide; Phase 3: Remdesivir, Lopinavir-Ritonavir, Darunavir ethanolate, Ribavirin, Sotrovimab, Sovoflurane, Dexamethasone, Plitidepsin, Blood Plasma; Phase 2/3: Azithromycin, Nafamostate mesylate, Tocilizumab, Glucocorticoid, Opaganib, Moulpinavir, mexthotrexate, Imatinib mesilate; Phase 2: Hydroxychloroquine, Favipiravir, Interferon, Disulfiram, Ebselen, sotrovimab, Thalidomide, Thiadiazolidione-8, Clofazimine, Brilacidin, Pluristem, Fluroxamin; Phase 1/2: Ivermectin, cosmostate mesylate, Interferon alpha; Phase 1: Galidesivir; Observational: Darunavir/Cobicistat, Famotidine, Methylprednisolone; Terminated: Remdesivir, Hydroxychloroquine, Azithromycin, Galidesivir, Nitrazoxanide, Tocilizumab, Propofol, Dexamethasone; Study type: Describes the nature of a clinical study. Study types include interventional studies (also called clinical trials), observational studies (including patient registries), and expanded access; Recruitment status: Not yet recruiting: This study has not started for recruiting the participants; Recruiting: This study is currently recruiting the participants; Enrolling by invitation: This study is selecting its participants from a population, or group of people, decided on by the researchers in advance. These studies are not open to everyone who meets the eligibility criteria but only to people in that particular population, who are specifically invited to participate; Active, not recruiting: The study is ongoing, and participants are receiving an intervention or being examined, but potential participants are not currently being recruited or enrolled; Suspended: The study has stopped early but may start again; Terminated: The study has stopped early and will not start again. Participants are no longer being examined or treated; Completed: The study has ended normally, and participants are no longer being examined or treated (that is, the last participant's last visit has occurred); Withdrawn: The study stopped early, before enrolling its first participant; Unknown: A study on ClinicalTrials.gov whose last known status was recruiting; not yet recruiting; or active, not recruiting but that has passed its completion date, and the status has not been last verified within the past 2 years. Latest update posted: The most recent date on which changes to a study record were made available on ClinicalTrials.gov. There may be a delay between when the changes were submitted to ClinicalTrials.gov by the study's sponsor or investigator; Phase: The stage of a clinical trial studying a drug or biological product, based on definitions developed by the U.S. Food and Drug Administration (FDA). The phase is based on the study's objective, the number of participants, and other characteristics. There are five phases: Early Phase 1 (formerly listed as Phase 0), Phase 1, Phase 2, Phase 3, and Phase 4. Not Applicable is used to describe trials without FDA-defined phases, including trials of devices or behavioral interventions; Sponsor: The organization or person who initiates the study and who has authority and control over the study. Enrollment: The number of participants in a clinical study. The "estimated" enrollment is the target number of participants that the researchers need for the study.

## Environmental implications of antiviral drug candidates

So novel drugs and existing drugs with an aim to repurpose are being produced in large amounts by the pharma industries. Due to pharmaco kinetic effects the administered drugs are bio-transformed inside the human body and then expelled out through urine and/or feces appearing in large quantities in the municipal waste water, In addition many of these manufactured products may be leaving the waste water plants from the pharma industries. Both these effluents may be polluting the ground water as well as the rivers and lakes. The degradation properties and toxicity of the novel drugs which are being discovered at a tremendous pace, by the pharma companies may not still be fully understood, thereby creating new problems. For example, anthracycline based drugs, which are recalcitrant, harmful, also act as teratogen and carcinogen, and thus carry lifelong risk to the aquatic species, soil environment, and eventually threats to human beings.

Antivirals have been observed at wastewater treatment plants (WWTP). Influenza pandemic simultaneously leads to increase in antiviral drugs in the water bodies. Chloroquine is potent against malaria and other viral pathogens, however 70% of this drug is excreted out as urine and feces which may spread through the wastewater, and is probably harmful to soil and water microbiota and also to soybean plants. These non-biodegradable drugs exhibit adverse properties on the aquatic biota and produce groundwater toxicity. Similarly hydroxychloroquine (HCQ) which was tested for the current pandemic gets bio transformed in the body and the hydrolyzed and glycosylated products are excreted through the urine and feces. Substantial escape of ivermectin through the WWTP, could be a serious threat. Dexamethasone prescribed for patients with chronic symptoms, pose a smaller risk to the environment. Azithromycin would increase the environmental impact and is already seen as a threat in some countries. The toxicity data for Favipiravir is minimal, although it is known for teratogenicity. It is lethal for animals. Ribavirin based nucleoside agents is usually practiced in China, based on PNEC (Predicted No Effect Concentration), *i.e*. amount tested against blue green- / green- algae. Increased dosages may distress the environmental development of green algae. Umifenovir possess chronic toxicity against aquatic organisms along with ecotoxicological hazard. Tocilizumab, based on SDS display no adverse effects observed at higher concentration [176].

### Gene therapy

The potential resuscitative treatments in the COVID-19 period is the *cell therapy*, involving allogeneic (Allo) and autologous (Auto) hematopoietic cell transplant methods. *Gene therapy* used to pursue / transform / activate the genetic expression/ to change the biological activities of living cells for therapeutic utility. I*mmunomodulators* are useful to pushing down the body’s specific immune reactivity to the virus, in some cases the body’s reaction which drives severe and start assaulting the patient’s own organs. The *neutralizing antibody therapies* may assist those who fight with the virus which includes contrived antibodies, animal-based antibodies and blood-derivatives (convalescent plasma and hyper-immune globulin) containing antibodies, obtained from previously affected COVID-19 individuals. Variety of therapeutic methods are under investigation, which are potential treatment strategies [177–180].

## Conclusions

Here we have assembled literature data regarding antiviral drug candidates. As on 2019, the emergence of the newly arrived *β*-coronavirus as SARS-CoV-2 from the Chinese region, has quickly spread across the globe to more than 396,558,014 confirmed cases as on 8 February 2022 along with 10,095,615,243 vaccine doses administered worldwide. SARS-CoV-2 illness has resulted in pneumonia and possibly may extend to multi-organ malfunctions fatality (COVID-19 syndrome), though this may be exaggerated, owing to insufficient information of the factual cases from all countries. Majority of cases have emerged as gentle or even not exhibiting any symptoms of disease, as a result of which exact number of cases remains unidentified. Because insufficient approved drugs or vaccines exist to majority of the populations till date, there is a compulsion to discover approved off-labeled and clinically proven drugs against this pandemic infection. Aforementioned drug candidates may represent inhibitors, whether traditional / existing / recognized / conventional anti-malarial, anticoagulant, anti-inflammatory, antiviral or other drugs / inhibitors of viral RNA polymerase, proteases and combining virus/host cell membrane system with antiviral activities. Testing individually or in combination/drug repurposing with interferon *β*-1a for COVID-19 is also the need of the hour.

Very recently, the FDA provided the Emergency Use Authorizations (EUAs) approval for two antiviral candidates (orally) for non-hospitalized COVID-19 patient’s treatment who are at very high risk of changing over to severe disease at milder to moderate stage. They are ritonavir-developed nirmatrelvir (commercially named as Paxlovid) and molnupiravir. These can be used for treating non-hospitalized patients with the currently available therapies. Once the resources are inadequate, treatment ought to be systematic for highly critical COVID-19 patients. Higher spreading of B.1.1.529 (Omicron) as variant of concern (VOC) has led to several complications.

The panel suggested the recent treatments in the following order: paxlovid (in oral dosage) everyday two times for five days. Sotrovimab, directed as intravenous (IV) infusion. Remdesivir on day 1, followed by remdesivir IV on Days 2 and 3, molnupiravir orally taken as daily twice for 5 days. Omicron VOC emerging as the prevailing variant at various regions of USA in recent days, since, they obviously minimized liability against SARS-CoV-2 monoclonal antibodies (mAbs) (bamlanivimab + etesevimab and casirivimab + imdevimab). But, sotrovimab, an additional mAbs, including Convalescent Plasma, Interferons, Colchicine, Fluvoxamine, IL-6 Inhibitors, Kinase Inhibitors: Janus Kinase Inhibitors and Bruton’s Tyrosine Kinase Inhibitors and molnupiravir may be expected to exhibit the potential activity against such SARS-CoV-2 and other variants. Remdesivir is anticipated to maintain its potential activity against the Omicron VOC. Similarly, nirmatrelvir, an antiviral drug (Developer: Pfizer) acts as an orally active 3CL protease inhibitor. Combined nirmatrelvir enclosed with ritonavir was approved by the US-FDA for the treatment against COVID-19. Besides, Evushe (AZD7442) with an aid of federal government, AstraZeneca isolating the couple of antibodies such as tixagevimab and cilgavimab, exhibited effective response against coronavirus, however the primary test results of this potent drug candidate against Omicron variant, indicates that it maintains its capability to induce an immune response. Range of drug candidates removed from clinical trials: Accordingly, both chloroquine and hydroxychloroquine are FDA-approved drugs for the treatment against malaria, and likewise against autoimmune conditions including rheumatoid arthritis, systemic lupus erythematosus in adults and chronic discoid lupus erythematosus. Moreover these drugs are safer and active after utilized for such diseases as per FDA-authorized labeling, but WHO revokes chloroquine, hydroxychloroquine and lopinavir/ritonavir, ivermectin are management weapons for COVID-19. Some of the removed drug candidates from the clinical trials are also listed concisely in this report (https://www.fda.gov/drugs/coronavirus-covid-19-drugs/coronavirus-treatment-acceleration-program-ctap , https://www.covid19treatmentguidelines.nih.gov/, https://clinicaltrials.gov/ct2/covid_view) [181–189]. 

## Supplementary Information

**Additional file 1: Figure S1.** An overall viral targets against the representative ninehuman viruses (HIV, HCV, RSV,IV, HBV, HPV, HCMV, HSV, VZV). **Figure S2.** Characteristic potential targets and drugs against SARS/MERS virus. **Figure S3.** Representative potential targets and drugs against zika virus. **Figure S4.** A potential target and drugs against dengue virus. **Table S1.** Representative antiviral drugs/inhibitors and their significant characteristics. **Table S2.** Different infectious diseases outbreaks during COVID-19 era. **Table S3.** Range of vaccines clinically approved by WHO*.

## Data Availability

Supplementary data is available.
