# Supplementary material for: Significant perspectives on various viral infections targeted antiviral drugs and vaccines including COVID-19 pandemicity
Source: Mol Biomed. 2022 Jul 15;3:21. doi: 10.1186/s43556-022-00078-z (PMC9283561; doi:10.1186/s43556-022-00078-z)
Supplement: Supplementary file 1 — Additional file 1: Figure S1. An overall viral targets against the representative ninehuman viruses (HIV, HCV, RSV,IV, HBV, HPV, HCMV, HSV, VZV). Figure S2. Characteristic potential targets and drugs against SARS/MERS virus. Figure S3. Representative potential targets and drugs against zika virus. Figure S4. A potential target and drugs against dengue virus. Table S1. Representative antiviral drugs/inhibitors and their significant characteristics. Table S2. Different infectious diseases outbreaks during COVID-19 era. Table S3. Range of vaccines clinically approved by WHO*. [file 43556_2022_78_MOESM1_ESM.docx]

**Supplementary Information**

**Significant perspectives on various viral infections targeted antiviral drugs and vaccines including COVID-19 pandemicity**

Gandarvakottai Senthilkumar Arumugam,^1†^ Kannan Damodharan,^1,2†^ Mukesh Doble,^1,3^*

Sathiah Thennarasu^2^

^1^Bioengineering and Drug Design Lab, Department of Biotechnology, Indian Institute of Technology Madras (IITM), Chennai, Tamilnadu, India - 600036.

^2^Department of Organic and Bioorganic Chemistry, CSIR- Central Leather Research Institute (CLRI), Chennai, Tamilnadu, India - 600020.

^3^Saveetha Dental College & Hospitals, Saveetha Institute of Medical & Technical Sciences, Chennai, Tamilnadu, India - 600077.

Correspondence: Mukesh Doble, [mukesh.doble0@gmail.com](mailto:mukesh.doble0@gmail.com)

^†^Gandarvakottai Senthilkumar Arumugam and Kannan Damodharan contributed equally to this work

**OVERVIEW OF NINE HUMAN VIRUSES**

**Human Immunodeficiency Virus (HIV)**: It is a lentivirus, which belongs to family of Retroviridae and discovered on 1983 with the leading cause of AIDS (*Acquired Immuno Deficiency Syndrome*). An advanced genetic sequential modification has perceived in the genomic HIV, thus creating the HIV is rapidly-developing organisms. Owing to the manifold zoonotic holding, it is identified to be spreading from *Pan troglodytes* (chimpanzee) (HIV-1 sets M and N), similarly *Gorilla gorilla* (gorilla) (HIV-1 sets P and O), and *Cercocebus atys* (sooty mangabey) (HIV-2) to human beings. Moreover HIV (blood-borne virus) spread mostly by its infected blood, or else body fluids; thus, HIV infectivity through sexual relationship, sharing of needles, transfusions of blood, or spreading through maternal. Several complications in clinical aspects have been documented as cardiovascular illness, gingivitis, kidney disorder, lung cancer, lymphoma, osteoporosis, papulosquamous problems and psychiatric turmoil etc., [1, 2].

**Hepatitis C Virus (HCV)**: In 1989, HCV (fam: Flaviviridae) was discovered, and it is a hepacivirus. This virus particle have the dimension of 68 nm in diameter, with a linear, positive-strand ssRNA genomic sequential viral proteins. In terms of concerning the HCV viral source still remains unknown, however, non-human primates, mammalians may have apparent zoonotic reservoirs. Since, the blood-borne HCV disease is mostly infected as similar to HIV. Throughout the virus acuteness, the incubation duration has more than a month (~7 weeks). Several clinical complications have examined as mainly liver-cirrhosis, malfunction, and high blood pressure and/or led to cancers in liver as hepatocellular carcinoma [3].

**Influenza Virus (IV)**: It belongs to the family of Orthomyxoviridae, and basis on the initial identification of this pandemic flu during 1510, however, the IV have isolated initially on the year 1933 that includes the various factors of the viral particle, therein it is a linear, negative-strand ssRNA genome with encoding eleven/twelve proteins, depends upon the viral strain. Besides, the influenza viruses are classified into three categories, such as influenza A, B, and C types, wherein the IV-A type 1 led to epi-/pan-demicities, for example the various flu infections and their year of findings were indicated as Spanish flu (1918), Asian flu (1957), and HongKong flu (1968), are subclassified into wide-ranging of serotypes as H1N1, H1N2, or H3N2 along with spreading potential from animal reservoirs. For example, *Aves* (e.g., H2N2, H5N1, H7N3, and H9N2), *Sus* (e.g., H1N1 and H3N2), or *Pinnipedia* (H7N7) to humans. Similarly, the IV-B type 2 sourced human outbreaks are separated into only strains; whereas, IV-C type 3 viruses did not responsible for any outbreaks, due to they generally transmit a disease to humans with mild poor health. During this IV associated viral infection has the characteristic incubation period of less than a week with an average of two days, furthermore, various clinical complications of bronchitis, dehydration, ear infections, encephalitis, pneumonia and sinusitis have noted [4, 5].

**Respiratory Syncytial Virus (RSV)**: It initially found in 1957, falls under the genus: Pneumovirus; family: Paramyxoviridae. This viral particle holds a linear, negative-strand ssRNA genomic sequence encodes 11 proteins. Apart, this viral strains have been divided into couple of antigenic subtypes A and B, further they subdivided on the basis of genotypes into 11 RSV-A and 23 RSV-B respectively. This virus receives respiratory path used for spreading through straightly infected aerosols/drops, however, they don’t have any animal reservoir, the incubation stage during infection ~5 days, and major complications held clinically including otitis bronchiolitis, pneumonia, respiratory tract infections and sinusitis are reported [6].

**Hepatitis B Virus (HBV)**: It was found in 1963 (genus: Orthohepadnavirus and family: Hepadnaviridae), hence the viral particle have a spherical dsDNA genomic array encoding six viral proteins and the strains behold eight genotypes and over 24 subtypes. About the beginning of this virus, which continues mysterious to be disclosed except bat may possess the hereditary basis of primate heap DNA viruses. [7] Similar to HIV and while a blood-borne virus, HBV can be spread through sexual relationship and other factors. Besides, the incubation period of infectivity three months with a range of two to five months. Many of the HBV infectivity lead clinical complications have perceived as abdominal pain, anorexia, arthralgia, hepatitis, liver cancer (hepatocellular carcinoma), nausea, rash, vomiting etc.,

**Human Papillomavirus (HPV)**: In 1965, it was apparently discovered, since the family of Papillomaviridae, which be the leading representative of cervical cancers, this viral particle, have aclosed, spherical, dsDNA genomic sequential array with nine viralproteins and moreover its strains have divided by 200 types on the basis of chain difference in the capsid protein L1. Since this virus associated infectivity responsible for anal-cervical- and penile- carcinoma. While less-threatening types of HPV-6 and 11 lead peripheral genital warts with periodic respiratory papillomatosis. Obviously, this HPV broadly observed in birds, mammals, reptiles and so forth. Besides, the duration of incubation by HPV infectivity to clinical warts differs in an average of ~ 70 days. Also, this viral diseases are spread mostly by closure skin-skin connectivity [8].

**Human Cytomegalovirus (HCMV)**: It was found in 1956, therein it have genus: Cytomegalovirus and family: Herpesviridae. This viral particle comprising of a linear dsDNA genetic material harboring ~ 200 to 250 orf's (open reading frames). This viral strains have four different geno types including gB1, gB2, gB3, and gB4 on the basis of series difference of the particular (UL55) glycoprotein B (gB) genetic codes. Without animal reservoir, this viral pathogen travel entirely to inhabitants, this virus have spreading ability by similar viral characteristics like transfusion of blood, fluids presents in the body, feeding in breast, transplantations of organ or sexual transmittances. Apart, the incubation stage during viral infections is more than ~21-84 days. Through this viral infectivity cause many clinical problems such as antigenemia, allograft infectivity, carditis, colitis, encephalitis, ependymitis, esophagitis, CNS/gastrointestinal disorder, hepatitis, nephritis, pancreatitis, pneumonia, retinitis etc. [9].

**Herpes Simplex Virus (HSV)**: This virus (genus: Simplex virus; family: Herpes viridae), was found on or before 1900, this viral particle comprises a linear dsDNA genetic material holding 84 genomes, and divided majorly into two types as HSV-1 and HSV-2 respectively. Amidst, the HSV-1 causes the herpes infectivity in oral region, lead to skin lesions and cold sores; whereas, the HSV-2 is generally liable for herpes infectivity in genital sections led to pain while urination and blistering sores. Later, the non-existence of animal reservoir, this virus travels to human inhabitants absolutely; spreading factors are intervened directly from polluted aerosols/droplets. Even if the viral infectivity lead the duration for incubation of HSV have ~96 hours, which typically the viral type-1 resulting encephalitis, keratitis, orofacial blisters or pneumonia, whilst the viral type-2 typically causes inflammation of the meninges or genital diseases [10].

**Varicella-Zoster Virus (VZV)**: Primarily, isolated on tissue culture way in 1953, from the genus of Varicella virus and the family of Herpes viridae. It is a linear dsDNA genetic material loading 73 genes, and viral strains are categorized into five clades along with nine genotypes. In the absence of animal reservoir, VZV pass through completely in human residents, spreading through respiratory pathway in straight manner by aerosols and droplets or lesions. Also, the viral infections associated incubation stage is ~ over 1 to 3 weeks, caused chickenpox with shingles or herpes zoster. Several clinical complications reported as cellulitis, cranial / peripheral nerve palsies, encephalitis, myelitis, neuralgia, and pneumonia [11].


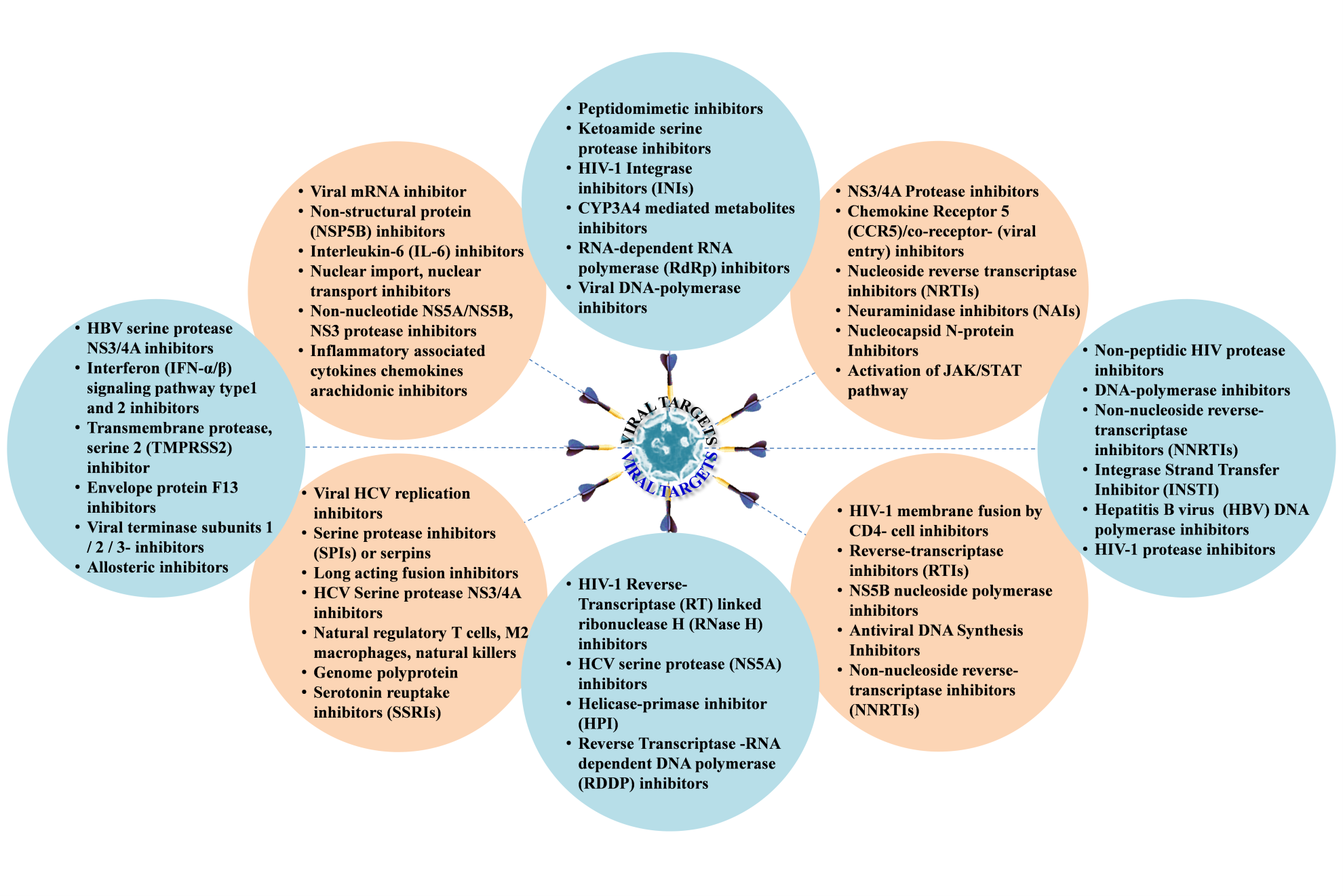


Supplementary Fig. 1. An overall viral targets against the representative nine human viruses (HIV, HCV, RSV, IV, HBV, HPV, HCMV, HSV, VZV)


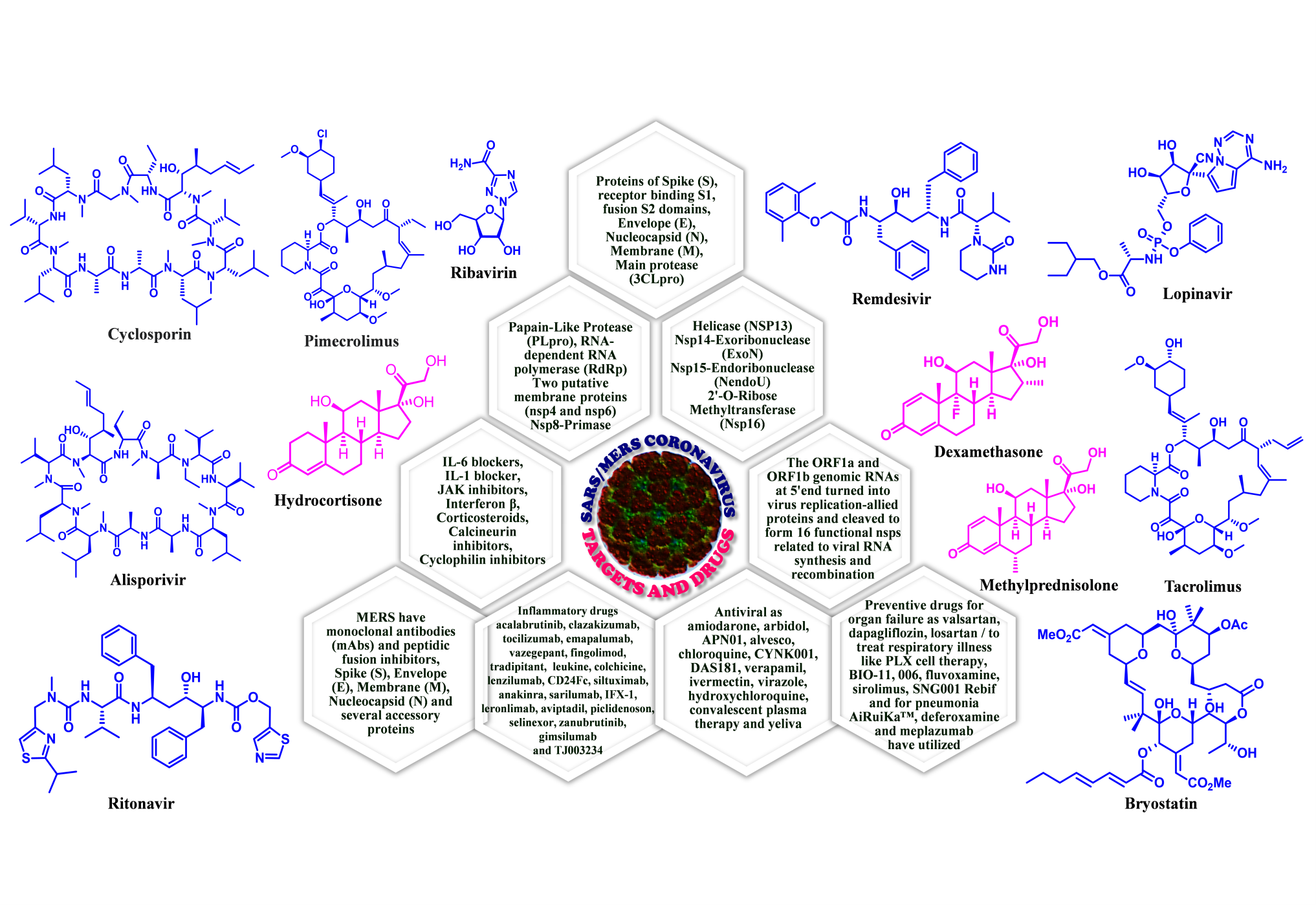


Supplementary Fig. 2. Characteristic potential targets and drugs against SARS/MERS virus as follows: SARS-CoV: Spike protein (S), Receptor binding S1 and the fusion S2 domains, Coronavirus Envelope (E) Protein, Nucleocapsid (N), Membrane (M) protein, Main Protease (3CLpro), Papain-Like Protease (PLpro), RNA-dependent RNA polymerase (RdRp), Two putative membrane proteins (nsp4 and nsp6), Nsp8-Primase, Helicase (NSP13), Nsp14-Exoribonuclease (ExoN), Nsp15-Endoribonuclease (NendoU), 2’-O-Ribose Methyltransferase (Nsp16); MERS-CoV: Specific therapeutic targets in S protein include N-terminal domain and RBD in S1, and heptad repeat 1 (HR1) and 2 (HR2) in S2. S1-RBD and S2-HR1 are main targets for developing anti MERS-CoV therapeutic monoclonal antibodies (mAbs) and peptidic fusion inhibitors respectively. MERS-CoV genome is a single, positive-stranded RNA encoding at least 10 open reading frames (ORFs), nine of them are expressed from seven sub genomic mRNAs (sgmRNAs), then translated into four major viral structural proteins, including spike (S), envelope (E), membrane (M), and nucleocapsid (N), as well as several accessory proteins, such as 3, 4a, 4b, 5 and 8b with unknown origins and functions. The ORF1a and ORF1b genomic RNAs at the 5’-end are translated into virus replication-related proteins and cleaved to produce 16 functional nonstructural proteins (nsps) that are related to viral RNA synthesis and recombination. Associated drugs are Pimecrolimus, Lopinavir, Ribavirin, Alisporivir, Cyclosporin, Ritonavir, Remdesivir, Bryostatin, Tacrolimus, Methylprednisolone, Dexamethasone, Hydrocortisone; Many of the drugs may have the inhibition capability with systemic hyper inflammatory activity for COVID-19 includes *IL-6 blockers*: Tocilizumab, Sarilumab; *IL-1 blockers*: Anakinra, Canakinumab, Heparins, Intravenous Immunoglobulins (IVIG (n), Hyper-immune Immunoglobulins eutralizing antibodies); *JAK inhibitors*: Ruloxitinib, Bariticinib; Corticosteroids: Methylprednisolone, Dexamethasone, Statins and recombinant human angiotensin-converting enzyme 2 (rh ACE2). Some of the single and combinations of drugs/inhibitors are influenced as Interferon β, Lopinavir and ritonavir, Lopinavir and ritonavir and Interferon β, Significant antiviral drugs and corticosteroids used for the treatment of SARS; Remdesivir is an active drug for MERS/SARS, and also may be effective for COVID-19 patients; *Calcineurin Inhibitors* (CNI), *Cyclophilin inhibitors*; Characteristic studies have been carried out against MERS-CoV and SARS-CoV; now, several actions are in practice to combat SARS-CoV-2, Treatments based on anti-viral drug development as arbidol, hydroxychloroquine, chloroquine, DAS181, amiodarone, verapamil, ivermectin, APN01, alvesco, CYNK001, virazole, convalescent plasma therapy and Yeliva. Based on anti-inflammatory drugs like leukine, tocilizumab, lenzilumab, CD24Fc, colchicine, tradipitant, siltuximab, anakinra, sarilumab, IFX-1, emapalumab, vazegepant, leronlimab, aviptadil, fingolimode piclidenoson, selinexor, acalabrutinib, clazakizumab, zanubrutinib, gimsilumab, TJ003234, galidesivir and convalescent plasma therapy. Several drugs employed for the treatment of pneumonia ensuing to viral infection as danoprevir + ritonavir, darunavir, ruxolitinib, bevacizumab, AiRuiKa™, tofacitinib, deferoxamine, and meplazumab. Certain drugs for preventing organ failure viz. valsartan, dapagliflozin, losartan or to treat respiratory illness, like PLX cell therapy, BIO-11,006, fluvoxamine, sirolimus, SNG001 and Rebif also been in utility

**Bryostatin**

**Alisporivir**

**Tacrolimus**

**Hydrocortisone**

**Dexamethasone**

**Methylprednisolone**

**Ribavirin**

**Remdesivir**


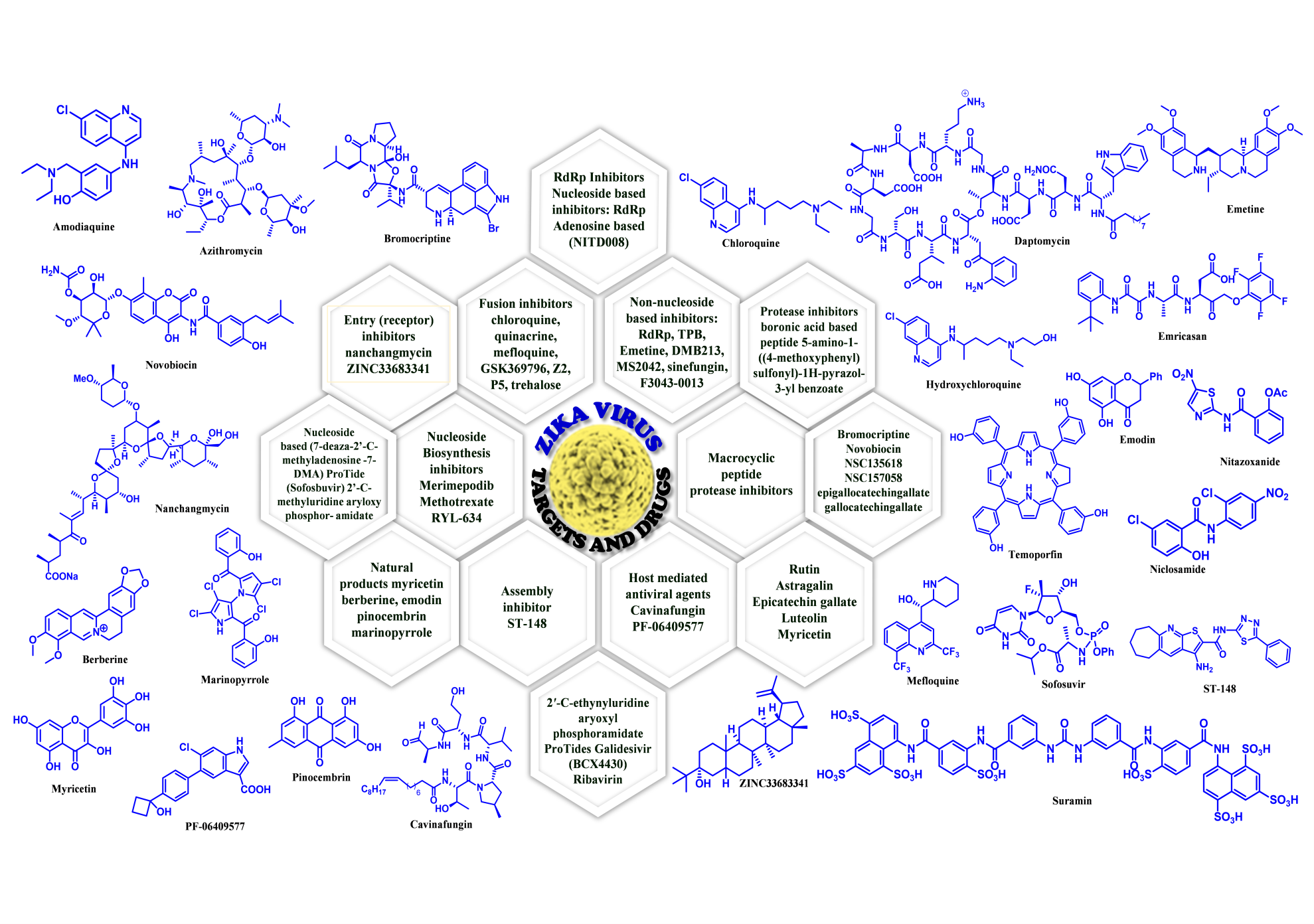


Supplementary Fig. 3. Representative potential targets and drugs against zika virus are as follows: Targets as RdRp Inhibitors, Nucleoside based inhibitors: RdRp Adenosine based (NITD008); Entry (Receptor) inhibitors: nanchangmycin ZINC33683341; Fusion inhibitors: chloroquine, quinacrine, mefloquine, GSK369796, Z2, P5, trehalose; Non-nucleoside based inhibitors: RdRp, TPB, emetine, DMB213, MS2042, sinefungin, F3043-0013; Protease inhibitors: boronic acid based peptide 5-amino-1-((4-methoxyphenyl) sulfonyl)-1H-pyrazol-3-yl benzoate; Nucleoside based (7-deaza-2’-C-methyladenosine -7-DMA) ProTide (Sofosbuvir) 2’-C-methyluridine aryloxy phosphor- amidate; Nucleoside Biosynthesis inhibitors: merimepodib, methotrexate, RYL-634; Macrocyclic peptide protease inhibitors; bromocriptine, novobiocin, NSC135618, NSC157058, epigallocatechin gallate, gallocatechin gallate; Natural products: myricetin, berberine, emodin, pinocembrin, marinopyrrole; rutin, astragalin,epicatechin gallate, luteolin, myricetin; Assembly inhibitor: ST-148; Host mediated antiviral agents: cavinafungin, PF-06409577; 2′-C-ethynyluridine aryoxyl phosphoramidate, ProTides, galidesivir (BCX4430), ribavirin; Associated drugs are amodiaquine, azithromycin, bromocriptine, chloroquine, daptomycin, emetine, novobiocin, hydroxychloroquine, emricasan, nanchangmycin, temoporfin, niclosamide, berberine, emodin, marinopyrrole, mefloquine, sofosuvir, ST-148, myricetin, PF-06409577, pinocembrin, cavinafungin, ZINC33683341, suramin


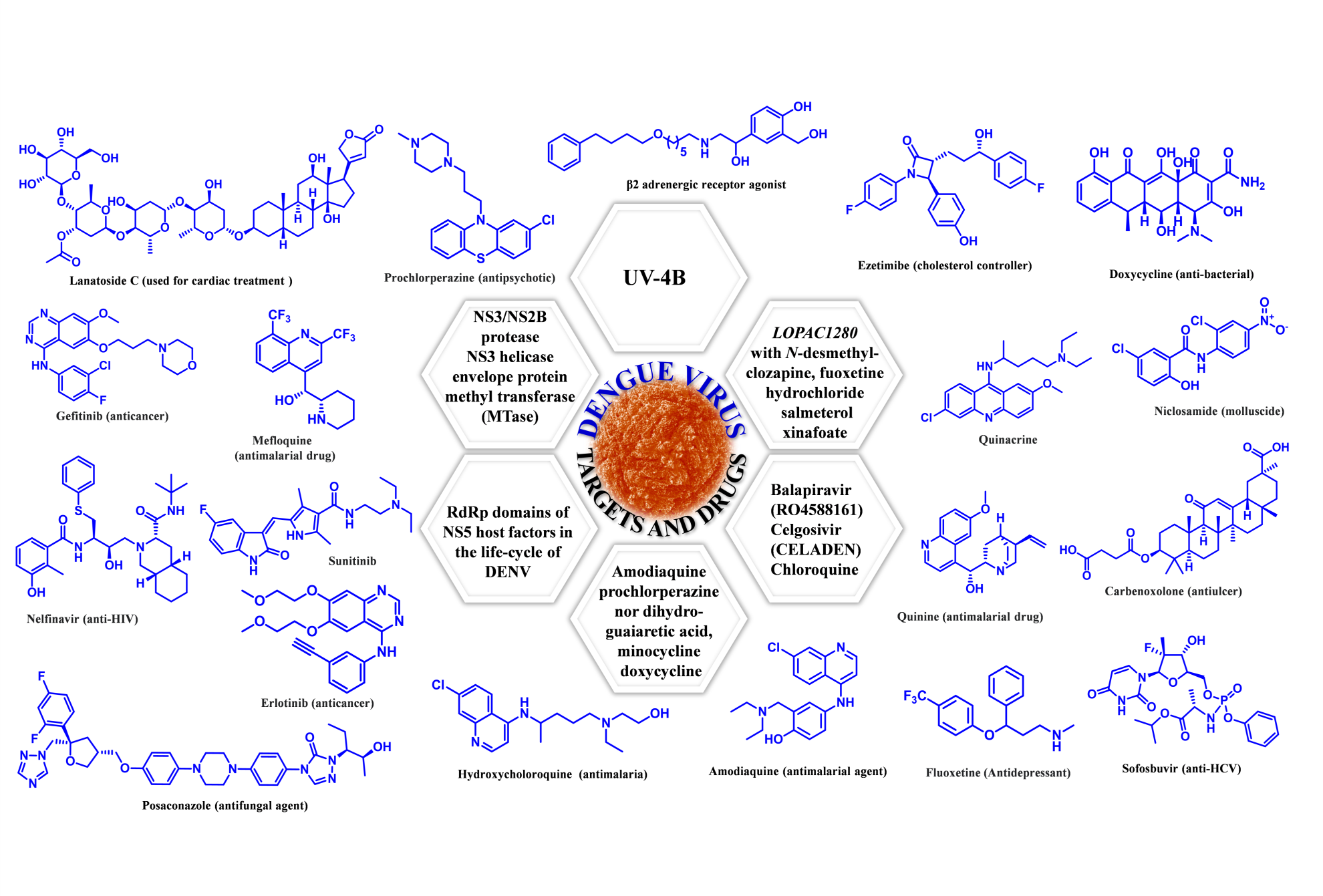


Supplementary Fig. 4 **a** Potential target and drugs against dengue virus as illustrated as UV-4B, LOPAC1280 with N-desmethyl-clozapine, fuoxetine hydrochloride salmeterol xinafoate, Balapiravir (RO4588161), Celgosivir (CELADEN), Chloroquine, amodiaquine, prochlorperazine, nordihydro-guaiaretic acid, minocycline, doxycycline, RdRp domains of NS5 host factors in the life-cycle of DENV, NS3/NS2B protease, NS3 helicase, envelope protein, methyl transferase (MTase); **b** Drugs and their significances are as follows: Lanatoside C (used for cardiac treatment), Prochlorperazine (antipsychotic), β2 adrenergic receptor agonist, Ezetimibe (cholesterol controller), Doxycycline (anti-bacterial), Gefitinib (anticancer), Mefloquine (antimalarial drug), Sunitinib, Quinacrine, Niclosamide (molluscide), Nelfinavir (anti-HIV), Erlotinib (anticancer), Quinine (antimalarial drug), Carbenoxolone (antiulcer), Posaconazole (antifungal agent), Hydroxycholoroquine (antimalarial), Amodiaquine (antimalarial agent), Fluoxetine (Antidepressant), Sofosbuvir (anti-HCV)

| **Supplementary Table 1** Representative antiviral drugs/inhibitors and their significant characteristics | | | | | |
| --- | --- | --- | --- | --- | --- |
| **Generic name** | **Trade name** | **Type** | **Mechanism of Action** | **Diseases** | **Year of**  **Approval** |
| **2-DEOXYURIDINE ANALOGUES** | | | | | |
| **Idoxuridine**  **Allergan 211** | Dendrid | Nucleoside analogues  Deoxyuridine  Iodinated thymidine analogues | Inhibit the viral replication by substituting itself against viral replication as thymidylate in viral DNA, Thymidine Kinase | HSV-1 infection | 1963 |
| **Vidarabine**  **DB00194** | Vira-A | Nucleotide antibiotic  Purine nucleoside analogues | Inhibition of viral DNA polymerase catalytic subunit; Thymidine kinase inducer; Termination of the growing viral DNA chain | HSV, VZV infection | 1976 |
| **Trifluridine (TFT)DB00432** | Viroptic  GSK | Fluorinated pyrimidine nucleoside | Thymidylate synthase inhibitor  Trifluridine gets incorporated into viral DNA during replication | HSV-1 | 1980 |
| **BrivudineRP-101** | Zostex, Mevir  Brivir | Pyrimidine-2-deooxy ribonucleosides | Thymidine kinase 2; the action of DNA polymerase blockage | HSV-1, 2  VZV virus infection | 2000 |
| **Entecavir**  **BMS200475-012** | Baraclude  (BMS) | Guanosine nucleoside analogues | Inhibits the activities of HBV DNA polymerase;  Base priming, reverse transcription of the negative strand from the pregenomic mRNA, Synthesis of HBV DNA in positive strand | HBV infection | 2005 |
| **Telbivudine**  **LDT-600** | Sebivo | Thymidine nucleoside analog | Inhibits HBV DNA polymerase (reverse transcriptase) / viral replication | Hepatitis B chronic infection | 2006 |
| **Clevudine**  **DB06683** | Levovir/ Revovir | Pyrimidine 2-deooxyribonucleosides analogues | Inhibition of viral with-standard DNA synthesis; Clevudine is phosphorylated by cellular kinases led to clevudine-triphosphate in target cells, reduces woodchuck hepatitis virus (WHV) replication | Hepatitis B (HBV) infection | 2007 |
| **PYROPHOSPHATE ANALOGUES** | | | | | |
| **Foscarnet**  **DB00529** | Foscavir | Pyrophosphate analogues  Phosphonomethanoic acid | DNA polymerase catalytic subunit inhibitor; Inhibiting the pyrophosphate binding region on virus specifically DNA polymerase, (Human herpes virus) HHV-5, HHV-1 | HCMV,HSV-type 1 and 2 | 1991 |
| **NUCLEOSIDE REVERSE TRANSCRIPTASE INHIBITORS (NRTIS)** | | | | | |
| **Zidovudine Azidothymidine (AZT)**  **BW-A-509U** | Retrovir | Nucleoside analogues  Synthetic deoxy- nucleoside | Inhibiting the action of HIV-1 reverse transcriptase via DNA termination after inclusion of nucleotide analogues (RNaseH) | HIV-1/AIDS  Mother to child spread | 1987 |
| **Didanosine (DDI)**  **BMY-40900** | Videx | 2', 3'-dideoxy nucleoside | Inhibits the HIV reverse transcriptase enzyme completely by competing with natural dATP and acts as DNA synthesis chain terminator (RNaseH) | HIV-1/AIDS/  HAART | 1991 |
| **Zalcitabine**  **(ddC)**  **Dideoxycystine**  **RO-24-2027/000** | Hivid | Nucleoside analogues  pyrimidine analogues | The dideoxycytidine 5′-triphosphate (ddCTP) impedes with viral RNA that focused DNA polymerase by contrasting with the natural dCTP deduction as well as inclusion of viral DNA (RNaseH); prevent the growth of viral DNA. | HIV-1/AIDS | 1992 |
| **Stavudine**  **BMY 27857** | Zerit | Dideoxynucleoside  thymidine analogues | Inhibiting the activity of HIV-1 reverse transcriptase together by competing with the natural dGTP and by addition in to viral DNA (RNaseH) | HIV-1/AIDS | 1994 |
| **Lamivudine**  **(3TC)**  **GR-109714X** | Epivir | Synthetic nucleoside analogues (-)-L-2',3' dideoxy-3-thiacytidine | Nucleoside units incorporated in to viral DNA by HIV reverse transcriptase and HBV polymerase resulting to DNA chain termination (RNaseH), protein inhibitor / HBV-F | HIV-1, HIV-2  AIDS/HBV | 1995 |
| **Lamivudine+**  **Zidovudine**  **(ZDV)** | Combivir | Nucleoside analogues  Dideoxynucleoside  (-)-L-2’,3’ dideoxhy-3-thiacytidine  Azidothymidine (AZT) analogues | Potent inhibitor of HIV-1 reverse transcriptase (RT) through DNA chain termination after integrating the nucleotide analogues. It competes with natural dGTP deduct and includes in to viral DNA; Inhibit and reduce the action of RT causing HIV infected cells | HIV infection in adults and children | 1997 |
| **Abacavir**  **1592U89** | Ziagen | Carbocyclic synthetic nucleoside analogues  Nucleoside analogues | Guanosine analogue, which have phosphorylate - carbovir triphosphate (CBV-TP), they enters in to the viral molecule and integrated with viral DNA, Once included into the viral DNA transcription then HIV-RT is inhibited (RNaseH). | HIV-1/AIDS | 1998 |
| **Tenofovir**  **Disoproxil**  **Fumarate** | Viread | Nucleotide analog | Reverse transcriptase inhibitors (NtRTIs) | HIV infection | 2001 |
| **NON-NUCLEOSIDE REVERSE TRANSCRIPTASE INHIBITORS(NNRTIS)** | | | | | |
| **Nevirapine**  **BIRG-0587** | Viramune | Dipyridodiazepinone | Nervirapine interact directly to reverse transcriptase with inhibiting RNA dependent and DNA dependent polymerase activity | HIV-1/AIDS | 1996 |
| **Delavirdine**  **(DLV)**  **U-90152 S** | Rescripter | N-acylpiprazine, Sulfonamide, aminopyridine  Indolecarboxamide | Delvairdine relates directly to viral reverse transcriptase and blocks RNA- dependent and DNA dependent polymerase actions by distrupting enzyme catalytic site (RNaSeH) | HIV-1 | 1997 |
| **Efavirenz**  **DMP-266** | Sustiva  Bristol-Myers-Squibb (BMS) | Synthetic purine derivatives | Inhibition of the viral RNA-directed DNA polymerase (RT) activities (RNaseH) | HIV-1/AIDS | 1998 |
| **Etravirine**  **TMC-125** | Intelence  Johnson & Johnson | 2,6-diammino pyrimidine | Inhibits the HIV-1 RT enzyme that openly attributes to reverse transcriptase and RNA dependent polymerase activity Gag-pol polyprotein | HIV-1 | 1998 |
| **Abacavir+**  **lamivudine +Zidovudine** | Trizivir  [Ziagen+  Epivir+  Retrovir] | Carbocyclic synthetic nucleoside analogue +Synthetic nucleoside analogues + Synthetic deoxynucleoside | Inhibits the viral reverse transcriptase (RT) and viral DNA growth is inhibited | HIV-1 | 2000 |
| **Emtricitabine**  **524-W-91** | Emtriva/  coviracil | 2,3'-dideoxy-5-fluro-3'-thiacytidine,  cytidine analogue | Emtricitabine (cytidine motif), once phosphorylated to emtricitabine 5'-triphosphate combats to deoxycytidine 5-triphosphate for HIV-1 RT as it incorporates emtricitabine into DNA strands formation, new nucleotides are incapable to incorporate, thus lead to viral DNA chain termination (RNaSeH) | Prevention and Treatment ofHIV-1 infection | 2003 |
| **Etravirine TMC125** | Intelence Johnson & Johnson | Diaryl pyrimidine | NNRTI- Gag-Pol polyprotein directly binds to RT and so obstructs DNA- and RNA dependent polymerase activity | Treatment of HIV type 1 (HIV-1) infection. | 2008 |
| **Rilpivirine Hydrochloride**  **TMC-128** | Edurant  Tibotec | Diarylpyrimidine derivative, | Binding of NNRTI resulting the blockage of RNA- DNA dependent DNA polymerase activity (RNaSeH) | HIV-1 infections | 2011 |
| **Doravirine**  **MK-1439** | Pifeltro  Merck | Pyridione  Oxybenzonitile | (HIV-1)-NNRTI, Reverse transcriptase (RNaseH); Doravirine hinders replication of HIV-1 through non-keenly inhibit HIV-1 RT | Treatment of HIV/AIDS | 2018 |
| **Elsulfavirine**  **TMC278** | Elipida Tibotec | Diaryl-pyrimidine | Non-nucleoside Reverse Transcriptase Inhibitors (NNRTI) used as a potential inhibitors | To prevent the HCV infection | 2017 |
| **PROTEASE INHIBITORS** | | | | | |
| **Saquinavir**  **(SQV)** | Invirase  /Fortovase | Member of quinolines and a L-asparagine derivative | By preventing the HIV-1 protease enzymatic activity, so the proteolysis of the Gag polyprotein saquinavir results the formation of immature, non-infectious viral particle; also act as the potent HIV-1 protease enzymatic inhibitor | HIV/AIDS | 1995 |
| **Ritonavir**  **A-84538** | Norvir | L-valine derivative  Peptidomiment | Ritonavir, an efficient cytochrome P450 CYP3A4 inhibitor, containing isoenzyme exist in intestinal tract and liver, Ritonavic have inhibiting HIV viral proteinase, normally separates the structured and replicating proteins emanate from main HIV genes of gag and pol | HIV-1, HIV-2/AIDS | 1996 |
| **Indinavir**  **DB0024** | Crixivan  Merck | Synthetic hydroxyl-aminopentane amide agent, Piperazinecarboxymide and dicarboxylic acid diamide | Inhibition of indinavir as the HIV-viral protease enzyme that prevents the gag-pol polyprotein cleavage resulting to non-infectious immature viral particles | HIV/AIDS | 1996 |
| **Nelfinavir**  **(NFV)**  **AG1343** | Viracept/  Agouron Pfizer pharmaceuticals Inc | Aryl sulfide  Member of benzamide,  a member of phenols,  a secondary alcohol, tertiary aminocompound, Organic heterobicyclic compound | Nelfinavir, a CYP3A inhibitor, polyproteins cleavage occurs due to protease, a useful step in the HIV life cycle. Later cleavage, the undeveloped viral proteins accumulated into particles grown from the cell as mature infectious virions, Protease inhibitors strive the active cleavage site on the protease, which hindering the polyproteins cleavage and new viral particles development | HIV-1 | 1997 |
| **Amprenavir**  **141W94** | Agenerase  GSK | Tetrahydrofurfuryl ester  A sulfonamide  A carbamate ester | Amprenavir inhibits the HIV viral protein enzyme through preventing the gag-polprotein cleavage resulted the noninfectious embryonic viral particles; Amprenavir forms complex with HIV protease due to an inhibitor-enzyme , thus prevents the usual maturation process of HIV and the developed infectious virions formation | HIV | 1999 |
| **Lopinavir-Ritonavir**  **ABT-378/538** | Kaletra/  Norvir  DB01601 | Peptidomimetic molecule antiretroviral | Lopinavir is the HIV-1 protease enzyme, an inhibitor whose design is based on the principle of peptidomimetic process, where mimics the molecule containing hydroxy ethylene scaffold. | antiviral,  HIV-1 drug, | 2000 |
| **Atazanavir**  **BMS-232632** | Reyataz | Azapeptide | Selectively atazanavir inhibiting the specific virus process of viral Gag and Gag-pol polyproteins in HIV-1 infected cells through binding the active site of HIV-1 protease, thus prevents the mature virions formation | HIV-1 infection | 2003 |
| **Fosamprenevir**  **GW-433908** | Lexiva | Aminobenzenesulfonamide | A prodrug that is promptly hydrolyzed to amprenavir by cellular phosphatases in the epithelial gut as it is absorbed. Amprenavir is a HIV-1 protease inhibitor. During the replication of HIV, this viral protease cleaves in to polypeptide viral products of the Gag-Gag-pol genes towards structural proteins virion core formation and necessary viral enzyme. Interference of amprenavir to the active binding sites of HIV-1 protease, so inhibit the processing of viral Gag and Gag-pol polyprotein precursors, results the immature non-infectious viral particles formation | HIV-1 infection | 2003 |
| **Tipranavir**  **DB00932** | Aptivus | Sulfonamide-containing dyhydropyrone | Tipranavir (TPV), a non-peptide HIV-1 protease inhibitor used for Gag and Gag-pol polyprotein processings in HIV infectious cells, prevents the mature virions growth | Treat HIV infection. | 2005 |
| **Darunavir**  **TMC 114** | Prezista | Aminobenzene sulfonamides | Darunavir is an inhibitor (HIV-protease), which prevents the replication of HIV-1 through binding, prevents the dimerization and catalytic activity of HIV-1 protease, particularly it inhibits the HIV-encoded Gag-pol proteins cleavage in cells that have been infected in the virus, prevent the formation of mature virus particles transmitting the infection | HIV-1 infection | 2006 |
| **Boceprevir**  **EBP-520** | Victrelis Schering-Plough, Merck | Synthetic tripeptide | NS3/4a serine protease inhibitor, genotype 1b, NS3 vital substance for viral replication, the inhibitory action of asunaprevir causes a robust antiviral activity | Hepatitis C Virus infection | 2011 |
| **Telaprevir**  **AIDS-213006** | Incivek vertex pharmaceuticals | Peptidomimetics Pyrazines cyclopentapyrrole | NS3/4A protease inhibitor useful for inhibition of viral HCV replication | Infection with Hepatitis C Virus (HCV) | 2011 |
| **Cobicistat**  **GS-9350** | Tybost | Monocarboxylic acid amide | Inhibition of CYP3A-mediated metabolism by capability of its substrates, atazanavir and darunavir thus ability to enhanced antiviral activity in a minimal dosage | HIV-1 infection | 2013 |
| **Simeprevir**  **TMC 435** | Sovriad | Azamacrocycle and  a lactam | NS3/4A protease splits the HCV polyprotein, succeeding of the NS3 site generate non-structural viral proteins NS3/NS4A, NS4B NS5A, NS5B and successive formation of mature proteins; Prevent viral maturation by protein synthesis inhibition | Hepatitis C virus (HCV) infection | 2013 |
| **Vaniprevir**  **MK-7009** | Vanihep Merck | Cyclic peptides Pyrrolidine carboxylic acid Isoindoline | Reversible HCV macrocyclic inhibitor of NS3/4Aprotease enzyme have potentially active against genotypes 1 and 2 NS3/4A protease | Macrocyclic Hepatitis C virus (HCV) Infection | 2014 |
| **Boceprevir**  **Simeprevir**  **Asunaprevir**  **Ribavirin+Peg-IFN-2b** | Victrelis  Olysio  Sunvepra  Vanihep | Synthetic tripeptide  Azamacrocycle and a lactam methoxy isoquinoline carbamate  Synthetic guanosine analogues | Protease enzymatic drugs of HCV able to  inhibit the proteolytic of HCV NS3/4a protease inhibitor that prevent viral HCV replication | HIV | 2011/2013  2014/2014 |
| **Paritaprevir**  **ABT 450** | Viekira Pak IBM Micromedex | Cyclopropyl-sulfonyl carboxamide phenanthridine | Inhibiting the NS3/4A serine protease of  HCV | Infection with Hepatitis C Virus (HCV) | 2014 |
| **Asunaprevir BMS-650032** | Sunvepra Bristol-Myers Squibb | Methoxyisoquinoline Carbamate | Inhibitor of the HCV serine protease enzyme NS3 genome polyprotein | Infection hepatitis C virus (HCV) | 2014 |
| **Darunuvir +**  **cobicistat** | Prezcobix | Aminobenzene sulfonamides+  Monocarboxylic acid amide | Selective inhibition of the cleavage spots in HIV-1 encoded Gag-pol polyproteins in infected cells, thus preventing the development of mature viral particles and stimulate CYP3A activity | HIV | 2015 |
| **Atazanavir+ cobicistat+**  **Telaprevir** | Evotaz  Incivek | Azapeptide mono- carboxylic acid amide peptidomimetics pyrazinescyclopentapyrrole | Cytochrome P450 (CYP) enzymatic inhibitor to the CYP3A family | HIV infection | 2015/2011 |
| **Grazoprevir MK 5172** | Grazyna  MERCK | Azamacrocyclic compound | Genotypes of 1 / 4 chronic HCV infect to adults. These essential enzymes responds to viral replication and assisting to split the viral encoded polyproteins in to developed proteins such as NS3, NS4A, NS4B, NS5A, NS5B | NS3/4A protein NS5Areplication complex inhibitor | 2016 |
| **Danoprevir RG-7227** | Ganovo Array BioPharma | Cyclic -α- peptide 15-memberedmacrocyclic peptidomimetic inhibitor | NS3/4A protease inhibitor genome polyprotein | HCV infection | 2018 |
| **INTEGRASE INHIBITORS** | | | | | |
| **Raltegravir**  **DB06817** | Potassium Isentress | New class of HIV drugs | Raltegravir inhibits the HIV integrase to inhibit the viral genome being combined in to human genome | HIV infection | 2007 |
| **Elvitegravir GS 9137** | Viteka Gilead Sciences | Quinoline monocarboxylic acid | Integrase strand transfer inhibitor (INSTI) | HIV-1 infection | 2013 |
| **Dolutegravir GSK572** | Tivicay | Monocarboxylic acid amide | HIV integrase strand transfer inhibitor (INSTI) | Treat HIV/AIDS | 2013 |
| **Dolutegravir+**  **abacavir+**  **lamivudine** | Trimeq | Monocarboxylic acid amide+2,6-diaminopurine+monothioazetal | Inhibit the integration of viral DNZ in to human chromosomes | HIV | 2014 |
| **Dolutegravir+**  **lamivudine** | Dutrimeq | Monocarboxylic acid amide+ monothioazetal | NRTIs be able to target HIV integrase and RT to intrude the viral replication | HIV | 2014 |
| **ENTRY INHIBITORS** | | | | | |
| **VZIG**  **Human Varicella-Zoster Immune Globulin** | VZV | Immunoglobulins | Human IgG encompassing antibodies to  varicella zoster virus affords inactive immunization intended for non-immune individuals screened to VZV thus decreasing the varicella infectivity severeness | Prevents the herpes zoster  VZV | 1981 |
| **RSV-IGIV**  **Respiratory l syncytialvirus immunoglobulin Intravenous**  **(sink-TEE-ahl)** | RespiGam | Immunoglobulins | Neutralizing antibodies of RSV may preclude fixing of its viral surface glycoprotein F and G; provisionally it works to enhance body’s immunity towards the virus, and also acts as  immunostimulants | RSV in younger children | 1996 |
| **Palivizumab** | Synagis  Medimmune | Protein based Therapies  Monoclonal antibody (mAb); By recombinant DNA technology | Palivizmab secures to the integrating glycoprotein of RSV, which prevents its tethering and acceptance by host cellular receptors; also a fusion glycoprotein F0 | RSV | 1998 |
| **Docosanol**  **IK-2** | Abreva | 22-carbon aliphatic alcohol | Docosanal systematic action by prevention of bonding between the human cell plasma membrane and the HSV envelop, so inhibiting the viral entry into cells and its replication; Envelope glycoprotein GP350 | RSV | 2000 |
| **Enfuvirtide**  **DP-178** | Fuzeon | Protein Based Therapies  Linear 36-amino acid synthetic peptide | Inhibiting HIV-1 fusion with CD4 cells  envelope glycoprotein | HIV-1 fusion | 2003 |
| **Maraviroc**  **DB04835** | Celsentri | Azabicycloalkan, triazoles organofluorine | HIV fusion inhibitor prevents HIV-1 entry through CCR5 co-receptor interaction. | Treatment of HIV infection, Liver injury | 2007 |
| **VariZig** | VZV | Immune Globulins  G (IgG) | IgG antibodies protect from VZV infection | VZV | 2012 |
| **ACYLIC GUANOSINE ANALOGUES** | | | | | |
| **Acyclovir (ACV)**  **NSC-645011** | Zovirax  Glaxo Welcom Inc | Nucleotide analog  Acylgunosine analogues  Oxopurine  2-aminopurine | Converted acyclovir to acyclovir triphosphate (ACV-TP) reasonably inhibits the viral DNA polymerase includes terminating the growth of viral DNA chain and deactivates the viral DNA polymerase | HSV-1 HSV-2 VZV, EMV  treatment, chickenpox, shingles | 1982 |
| **Ganciclovir**  **BW-759U** | Cytovene/  Zirgan | Acyclic nucleoside analogue of 2-deoxyguanosine | Ganciclovir active against CMV, in which inhibit the replication of viral DNA by ganciclovir-5’-triphosphate includes a selective potent inhibition of the viral DNA polymerase thymidine kinase | HCMV  AIDS associated withcytomegalo- virus infection | 1989 |
| **Famciclovir**  **AV 42810** | Famvir  Novartis | Guanosine analogues | *In vitro* studies shows that penciclovir triphosphate inhibits HSV-2 DNA polymerase combatively with deoxyquanosine triphosphate, then Herpes viral DNA synthesis and so replication are selectively inhibited DNA polymerase catalytic subunit inhibitors (HHV-1) | To treat herpes zoster (shingles) | 1994 |
| **Valacyclovir**  **256 U 87** | Valtrex  GSK | L-valine ester of acyclovir, Purine nucleoside analog | DNA polymerase inhibitor of  acyclovir triphosphate inhibits the virus DNZ polymerase and include this agent results in DNA chain termination prevent viral DNA synthesis and block virus replication; The inhibitory experiences of acyclovir are highly selective owing to the strong affinity of drug for thymidine kinase (TK) | HSV-1, 2, VZV, EBV, CMV | 1995 |
| **Penciclovir**  **BRL-39123** | Denavir | Guanosine analogues  Nucleoside analogues | Inhibit viral DNA synthesis, penciclovir triphosphate inhibits viral DNA polymerase by competing with deoxyguanosine triphosphate, Inhibition of DNA synthesis of virus-infected cells and replication | HSV | 1996 |
| **Valganciclovir**  **DB01610** | Valcyte | Guanine triphosphate | Ganciclovir’s virustatic activity is due to inhibition of viral DNA synthesis by presence of ganciclovir triphosphate | Cytomegalo-virus (CMV) infections | 2001 |
| **ACYLIC NUCLEOSIDE PHOSPHONATE ANALOGUES** | | | | | |
| **Cidofovir**  **GS-0504** | Vistide | Nucleoside analogue  Synthetic acyclic  Monophosphate nucleotide analogues of deoxycytidine | Cidofovir diphosphate potentially inhibiting viral replication through selectively inhibit viral DNA polymerases; since it have structural similarity together nucleotides, which competes deoxycytosine-5-triphosphate (dCTp) for DNA polymerase and gets included into the growing viral DNA strands | HCMV/AIDS  Cystomegalovirus (CMV) | 1996 |
| **Adefovir Dipivoxil** | Hepsera | Acyclic nucleotide analog  of adenosine monophosphate | Inhibitors of DNA polymerase/reverse transcriptase | Anti-HBV activity | 2002 |
| **Tenofovir disoproxil fumarate+**  **emtricitabine** | Truvada | Isopropyl L-alaninate derivative Phosphoryl group+  cytidine analogue | Emtricitabine restrains RT, an enzyme that mimics HIV RNA into new viral DNA. It also reduces the HIV amount and upsurges the immune system cells in the patient’s body. Tenofovir disoproxil fumarate blocks the RT | HBV | 2004 |
| **Tenofovir disoproxil fumarate+**  **Emtricitabine+ efavirenz** | Atripla | Synthetic nucleoside analog of cytidine  analogue of adenosine-5’-monophosphate  thio analog of cytidine  bisisoprophylcarbonyloxymethyl ester | Efavirenz is a NNRT inhibitor of HIV-1.  Emtricitabine is based on synthetic nucleoside motif, when cytidine is phosphorylated by cellular enzyme led to emtricitabine 5’-triphosphate | HIV | 2006 |
| **Tenofovir disoproxil fumarate+**  **Emtricitabine+ elvitegravir+ cobicistat** | Stribild | Isopropyl L-alaninate derivative Phosphoryl group+  Analog of cytidine+  Benzoxazin-2-one+  monocarboxylic acid amide | Tenofovir disoproxil fumarate blocks the RT,  Emtricitabine control RT, an enzyme that reproduce HIV RNA into newly formed viral DNA; Elvitegravir, an HIV-1 integrase strand transfer inhibitor; Cobicistor inhibitor of cytochrome P450 (CYP) enzymes of CYP3A | HIV | 2011 |
| **Tenofovir disoproxil fumarate+ cobicistat Emtricitabine+ elvitegravir** | Genvoya | Isopropyl L-alaninate derivative Phosphoryl group+ monocarboxylic acid amide+Analog of cytidine+Benzoxazin-2-one | Tenofovir disoproxil fumarate blocks the RT  Elvitegravir is an HIV-1 integrase stand transfer inhibitor; Cobicistor a mechanism based inhibitor of CYP enzymes of CYP3A  Emtricitabine regulate RT, an enzyme that replicate HIV RNA into new viral DNA | HIV | 2015 |
| **Tenofovir adafenamide+ Emtricitabine+**  **rilpivirine** | Odefsey | Isopropyl L-alaninate derivative Phosphoryl group+ Analog of cytidine+pyrimidinyl amino benzonitile | Inhibits the activity of HIV Reverse transcriptase (RNaseH) DNA polymerase inhibitor HCV-1  Emtricitabine control RT enzyme that reproduces HIV RNA to new viral DNA  Rilpivirine is another group of NNRTI with developed potency | HIV | 2016 |
| **Tenofovir adafenamide+**  **emtricitabine** | Descovy | Isopropyl L-alaninate derivative Phosphoryl group+ Analog of cytidine | Inhibits the activity of HIV Reverse transcriptase (RNaseH) DNA polymerase inhibitor HCV-1  Emtricitabine restrains RT, an enzyme that replicates HIV RNA into new viral DNA | HIV | 2016 |
| **Tenofovir Alafenamide Fumarate**  **(TFA-Ala)**  **GS-7340** | Vemlidy Glide Science | Isopropyl L-alaninate derivative Phosphoryl group | Inhibits the activity of HIV RT (RNaseH) DNA polymerase inhibitor HCV-1 | Chronic Hepatitis B virus (HBV) infection | 2016 |
| **NS5A AND NS5B INHIBITORS** | | | | | |
| **Sofosbuvir+**  **ribavirin** | Sovaldi | Nucleotide analog,  Guanosine analogues | Inhibition of NS5B polymerase and HCV 2, 3 replication; Inhibits viral RNA and protein synthesis | HCV | 2013 |
| **Sofosbuvir**  **GS-461203** | Sovaldi Gilead Sciences | Nucleotide analog | RdRp inhibitors NS5B polymerase | Hepatitis C Virus (HCV) infection | 2013 |
| **Sofosbuvir+**  **Ribavirin+**  **pegIFNα** | Solvadi | Nucleotide analog,  Guanosine analogues  Protein based therapies | Inhibits NS5B polymerse and replication of HCV 1, 4 | HCV | 2013 |
| **Daclatasvir dihydrochloride** | DaklinzaBristol-Myers-Squibb | Biphenyls, imidazoles, carbamate ester | Prevents RNA replication virion assembly through binding to NS5A | Chronic HCV genotype 1 and 3 infection. | 2014 |
| **Daclatasvir+**  **Asunaprevir**  **BMS-650032** | Daklinza+  Sunvepra | Biphenyls, imidazoles, carbamate ester  Oligopeptide | Inhibits NS5A and NS3/4A protease and prevent HCV 1, 4 replication | HCV | 2014 |
| **Daclatasvir dihydrochloride** | DaklinzaBristol-Myers-Squibb | Biphenyls, imidazoles, carbamate ester | Prevents RNA replication virion assembly thrrough binding to NS5A | Chronic HCV genotype 1 and 3 infection. | 2014 |
| **Daclatasvir+**  **sofosbuvir** | Harvoni | Biphenyls, imidazoles, carbamate ester nucleotide analog | Inhibits HCV NS5A and NS5B polymerase and prevent RNA replication | HCV | 2014 |
| **Daclatasvir+**  **simeprevir** | Sovodi  Olysio | Biphenyls, imidazoles, carbamate ester macrocycle | Inhibits HCV NS5B and NS3/4 protease | HCV | 2014 |
| **Dasabuvir**  **ABT 333** | Exviera | Dihydropyrimidinnaphthalen-methylsulfonyl-amide | Inhibitors of Non-nucleoside NS5B  NSP5B | Infection with Hepatitis C Virus (HCV) | 2014 |
| **Omibitasvir+**  **dasabuvir+paritaprevir+ritonavir** | Viekira park | Pyrrolidine-oxobutan-carbanmate  Synthetic acylsulfonamide  L-Valine derivative | Inhibitors of HCV NS5A, NS5B polymerase and NS3/4A protease | Infection with hepatitis C virus (HCV) | 2014 |
| **Ledipasvir**  **GS 5885** | Harvoni Gilead Sciences | Benzimidazol Fluoren carbamate | Inhibiting the HCV NS5A protein synthesis | Infection with Hepatitis C Virus (HCV) | 2014 |
| **Ombitatsvir**  **ABT 26** | Viekira Pak AbbVie | Aromatic heteromonocyclic compounds carbamate ester pyrrolidines aromatic amide | Inhibitors of NS5A, a protein need for viral replication and virion assemblage | Infection with Hepatitis C Virus (HCV) | 2014 |
| **Omibitasvir+ +paritaprevir+**  **ritonavir** | Technivie | Pyrrolidine-oxobutan-carbanmate, Synthetic acylsulfonamideL-Valine derivative | Inhibitors for HCV genotype NS5A, NS5B polymerase and NS3/4A protease | Infection with hepatitis C virus (HCV) | 2015 |
| **Daclatasivir+**  **sofobuvir** | Daklinza+  sovaldi | Biphenyls, imidazoles, carbamate ester  Nucleotide analog | Inhibits HCV genotype NS5A, NS5B polymerase and NS3/4A protease | Infection with hepatitis C virus (HCV) | 2015 |
| **Elbasvir+**  **Grazoprevir** | Zepatier | Organic heterotetracyclic compound  Azamacrocyclic compound | HCV, NS5A inhibitor selectively inhibit HCV  NS3/4a protease with wide activity through genotype and resistant variants | Infection with hepatitis C virus (HCV) | 2016 |
| **Elbasvir**  **MK 8742** | Erelsa Merck | N-acylpyrrolidine Imidazoles carbamate ester | Potentially active mechanism of NS5A inhibitors of ekbasivir include encumbering signal interactions reissuing NS5A from the endoplasmic reticulum to the surface of lipid drops and HCV replication complex change | Infection with hepatitis C virus (HCV) | 2016 |
| **Beclabuvir**  **/Asunaprevir**  **/Daclatavir**  **(DCV/ASV/BCV)**  **BMS-791325/BMS-650032/** | Multi-class combination of drugs asBristol-Myers Sqibb (BMS) | Benazepine caboxamide L-prolinamide, isoquinoline, Dicarbamate, pyrrolidine, immidazole | Allosteric inhibitor non-structural (NS5A), NS3 protease, Non nucleotide (NS5B) inhibitor | Hepatitis C virus genotype infection with compensated cirrhosis | 2016 |
| **Narlaprevir** | Arlansa | Treatment of chronic HCV | Inhibiting NS3/4A serine protease | HCV infection | 2016 |
| **Sofosbuvir/Velpatasvir GS-461203** | Epclusa  Gilead Sciences | Triphosphate nucleotide | Inhibiting non-structural protein 5A (NS5A), RdRp | Hepatitis C infection | 2016 |
| **Sofosbuvir/Voxilaprevir/Velpatavir**  **S900007740** | Vosevi  (Voh-SEV-ee)  Glide Sciences | Terta azapentacyclo carboxamide | NS5 polymerase-NS34A inhibitors | Hepatistis C genotype 1,2,3,4,5 or 6 infection, with and without Cirrhosis | 2017 |
| **Glecaprevir / Pibrentasvir** | Maviret AbbVie | Benzimidazole Pyrrolidine carbamate | HCV-NS3/NS4A/ NS5A inhibitor | Treatment for HCV infection | 2017 |
| **INFLUENZA VIRUS INHIBITORS** | | | | | |
| **Amantadine** | Gocovri  Symmrtrel  Symadine | 1-adamantylamine  Synthetic tricyclic amine | Dyskinesia linked with parkinsonism and IV-A  Antagonism of the influenza virus A M2 proton channel prevent viral shedding,  Inhibition of N-methyl D-aspartic acid (NMDA) receptor mediated inducement of acetycholine results antihyperalgesic | Influenza virus A  Antiparkinsonian  antihyperalgesic | 1966 |
| **Ribavirin** | Copegus Rebetol Virazole | Synthetic quanosine analogues | Inhibitor of HCV polymerase and possesses a wide ranging of activity against DNA and RNA;  inhibiting the mRNA synthesis | HCV, RSV, hemorrhagic fever | 1985 |
| **Rimantadine**  **(INN)** | Flumadine | Alpha methyl adamantane  methyalamine  Amantadine are derivatives of adamantine | Inhibiting the uncoating of the virus.  The protein coded by the M2 gene of influenza may play a substantial role in rimantadine susceptibility, RNA synthesis inhibitor | Influenza virus A  prophylaxis | 1993 |
| **Zanamivir**  **GG-167** | Relenza  Glaxo  Australian Biotech | Sialic acid analogues  Guanido neuraminic acid  2-deoxy-2,3-didehydro-N-acetylneuraminic acid (DANA) | Neuraminidase inhibitor  Through binds and inhibits the neuraminidase protein reduces the influenza virus unable to discharge its host cell and infect others; Possibility of change virus and inhibits the neuraminidase protein | Blocks the both entry of  Influenza virus A and B | 1999 |
| **Oseltamivir**  **GS-4104** | Tamiflu | Cyclohexene carboxylate ester that is the ester of oseltamivir acid | Antiviral neuraminidase inhibitors  Oseltamivir carboxylate is an effective inhibitor and selectively influenza virus, neuraminidase glycoprotein seen on surface of the virion; Oseltamivir activity renders viral shedding and infectivity | Prophylaxis of infection with Influenza virus A (H1N1) and B | 1999 |
| **Favipiravir T705** | Avigan | Pyrazine analog | RNA-dependent RNA polymerase (RdRp) | Influenza A and B | 2006 |
| **Laninamivir Octanoate**  **CS-8958** | Inavir | Fatty acid esters | Neuraminidase inhibitor (NAIs) | Prophylaxis of influenza A & B virus | 2010 |
| **Peramivir**  **RWJ 270201** | Peramiflu  BioCryst | Cyclopentane | Neuraminidase inhibitor (NAIs) | Activity against influenza A and B viruses | 2010 |
| **INTERFERONS, IMMUNOSTIMULATORS, OLIGONUCLEOTIDES AND ANTIMITOTIC INHIBITORS** | | | | | |
| **Pegylated interferons alfa 2b** | Peglntron  /Sylatron  Intron A | Protein based therapies  Interferons | It binds and to trigger the human interferon’s receptors lead to dimerization. This initiates the JAK-STAT signaling pathway; thus JAK/STAT method increases the expression of multiple genes in many tissues involved in the innate antiviral response and also activates the nuclear factor kB pathway | HBV/HCV/ Melanoma | 1986 |
| **Interferon**  **alfacon-1** | Infergen/IFN Alfacon-1  Valent pharmaceuticals | Protein based therapies | Interferon-*α* attributes to interferon receptor type (IFNAR-1 and IFNAR-2c), which gets dimerization motivates two jak (janus kinase), tyrosine kinase (jak-1 and jak-2). | HCV | 1997 |
| **Imiquimod (INN)**  **R 837** | Aldara  3M pharmaceuticals | Imidazoquinoline fused [4,5-c]  Carrying isobutyl and amino substituents at N-1 and C-4 | Immune response transformer act as a toll-like receptor 7 agonist, Imiquimod induce the cyctokins as well as several IFNA genes of IFNA1, IFNA2, IFNA5, IFNA6 IFNA8 and IFNB. Imiquimod also develop the expression of interleukin IL-6, IL-8 and TNF-α genes | HPV disease | 1997 |
| **Pegylated interferons**  **alfa- 2b**  **+ribavirin** | Rebetron  [Rebetol+  [Intron]  Schering Corp | Combination Therapy  Nucleoside analogues | Inhibition of HCV- RNA by combination therapy | HCV | 1998 |
| **Fomivirisen** | Vitravene  NIH/Isis pharmaceuticals | Gene therapies  Antisense oligonucleotides | Fomivirsen, a phosphorothioate oligonucleotide motif that inhibits the human cytomegalovirus (HCMV) replication through mechanism of antisense, when binding occurs to the target mRNA Formivitisen inhibits the IE2 protein synthesis and interrupts viral replication | CMV/HPV/AIDS disease | 1998 |
| **Peginterferon**  **Alfa-2a** | Pegasys | Protein Based Therapies | Activator of JAK/STAT pathway, Interferon alpha/beta pathway receptor 1 and 2 | Infection with Hepatitis C Virus (HCV) | 2001 |
| **Immuno globulin (IgG)(IV) Intravenous and subcutaneous** | Gammagard Liquid Baxter healthcare | Human plasma | It contains antibodies to protect itself against infection from disease, Immunoglobulin therapy for primary immunodeficiency | To treat primary immunodeficiency, multifocal motor neuropathy (MNN) in adults | 2005 |
| **Sinecatechins** | Veregen | Catechins Gallate | Immuno-modulator interfere with HSV related pathway | HPV disease | 2006 |
| **Immunoglobulin (intravenous) IGIV-HB** | Bivigam, Carimune | Immune globulin is sterile solution made from plasma, antibody protect against various infectious diseases | IGIV is used to treat primary immunodeficiency and increase the platelets | IGIV used to prevent certain infections in people with B-cell chronic lymphocytic leukemic | 2009 |
| **Letermovir**  **AIC 246** | Prevymis MERCK | 3,4-dihydroquina- zolinyl acetic acid | Viral terminase, subunit-1, 2, 3 inhibitors | Prophylaxis of Cytomegalovirus (CMV) infections | 2017 |
| **Ibalizumab**  **TNX-355** | Trogarzo-Tanox | Protein Based Therapies Monoclonal antibody (mAb) | Post-attachment inhibitor, viral-entry inhibitor T-cell surface glycoprotein CD4, C-C chemokine receptor type 5, C-X-C chemokine receptor type 4 | HIV infection | 2018 |
| **Rifn-2ab**  **SCH 305000** | Novaferon | Protein Based Therapies Interferons | Interferons alpha/beta receptor 2, Interferons alpha/beta receptor-1 | Treatment for Chronic Hepatitis B virus | 2018 |
| **Albuvirtide (ABT) FB006M** | Aikening Frontier Biotechnology | Meleimide modifies peptide | Long -acting Fusion inhibitor | Treatment of HIV infection | 2018 |
| **Baloxavir Marboxil (BXM)**  **S-003188** | Xofluza Scionogi Japan | Aromatic Hetero cyclic compounds Dibenzothiepins | Inhibitor of the influenza CAP-dependent endonuclease enzyme | To prevent the influenza A & B flu viruses H1N1 | 2018 |
| **Tecovirimat**  **ST-246, TPOXX** | Arestvyr | Azatetracyclo  (trifluoromethyl) benzamide | Envelope protein F13 inhibitor, Inhibiting the Orthopoxvirus VP37 envelope wrapping protein | Orthopox viruses such as smallpox and monkey pox. | 2018 |

**Note**. Despite various antiviral characteristic drugs/inhibitors are available, few are mentioned in this table; <https://go.drugbank.com/drugs>

**Supplementary Table 2**. Different infectious diseases outbreaks during COVID-19 era

| **Name of the infectious disease** | **Sources** | **Characteristic symptoms/causes of infectious disease** |
| --- | --- | --- |
| COVID-19 | SARS-CoV-2 | Fever, dry cough, and pervasive tiredness; though patients have listed aches plus pains, diarrhea plus conjunctivitis, headache, anosmia, plus ageusia as well as rashes |
| Typhoid  (Enteric fever) | Gram Negative Bacteria  *Salmonella typhi* | Continuous fever, chills, liver and spleen enlargement (hepatosplenomegaly) and abdominal pain, led to anorexia, rash, headache, diarrhoea/constipation, nausea, relative bradycardia and low consciousness level |
| Zika virus disease (ZVD) | Flaviviridae species that uses arthropod vectors for transmission. | Fever, joint and muscle pain, rash, headache, conjunctivitis, nausea, vomiting, and general malaise |
| New African strain of the ZIKV | Infected *Aedes aegypti* mosquitoes | Fever, headache, rash, pruritus, cough, myalgia, sore throat, arthralgia, hypogeusia, hyposmia, conjunctivitis, gastrointestinal symptoms (Abdominal pain, nausea, vomiting) |
| Dengu Fever (DENV) | Female mosquitoes of *Aedes aegypti* | Headaches, muscles ache, joint pains, nausea, vomiting, and rash |
| Tuberculosis (TB) | Bacteria  (*Mycobacterium tuberculosis*) | Cough, fever, night sweats or weight loss |
| Marburg virus disease (MVD) | Marburg virus (MV) | Severe hemorrhagic/high fever, headache and malaise, muscle pains are common features, Severe watery diarrhoea, abdominal pain, cramping nausea and vomiting can start on the third day |
| Ebola virus disease (EVD) | Ebola virus,  Filoviridae family | Fever, Fatigue, Muscle pain, Headache, Sore throat,Vomiting, Diarrhoea, Rash, Symptoms of impaired kidney and liver function, Low white blood cell and platelet counts and elevated liver enzymes. |
| Malaria | Female *Anopheles* mosquitoes. *Plasmodium* *falciparum* and *Plasmodium* *vivax* | Fever, chills, headache, sweats, vomiting, and body throbs. |
| Mucormycosis | Mucormycetes | Black fungus aims the sinus / lung after fungal spores, inhaled from the air and affect skin after a surface injury like a cut or a burn. Symptoms mainly depend on where the fungus growing or infiltrated in the body and can spread to eyes and brain, leading to catastrophic outcomes. |
| HIV/AIDS | Human immunodeficiency virus (HIV) | Swollen lymph nodes, weight loss, fever, diarrhoea and cough, infection increasingly weakens the immune system, if not treated led to tuberculosis (TB), Cryptococcal meningitis, severe bacterial infections, and cancers such as lymphomas and Kaposi's sarcoma. |
| Measles | Measles virus (MV)  paramyxovirus family | Usually high fever, running nose (Rhinorrhea), cough, red and watery eyes, white spots develop in cheeks initially. Serious problem contain blindness, encephalitis (an infection led to brain swelling), severe diarrhoea and related dehydration, ear infections, severe respiratory infections like pneumonia. |
| Lassa Fever (LF) | Lassa fever virus (LFV) | Sore throat, pains in the joints, back and chest, and weakness,  pharynx inflammation, conjunctivitis, abdominal pains, diarrhoea, vomiting and clinical manifestations that range from facial drops to respiratory symptoms, loss of hearing, acute hemorrhagic fever, multiple-organ failure, foetal loss and death |
| White fungus | *Candida auris* and  *Candida albicans* | Spreading of *Candida* infection in bloodstream leads systemic infection, affect organs like skin, intestines, genitals and kidneys. |
| Yellow fever (YF) | *Aedes* and *Haemagogus* mosquitoes, | Commonly fever, muscle pain with backache, headache, loss of appetite and nausea/vomiting.  High fever endures and body systems are affected, usually liver and kidneys.  People are probable to develop jaundice (yellowing of the skin and eyes, therefore termed as ‘yellow fever’ |
| Nipah | Nipah virus (NiV) | Fever, headaches, myalgia (muscle pain), vomiting and sore throat. Followed by dizziness, drowsiness, altered consciousness, and neurological signs that display acute encephalitis. Someone can also experience abnormal pneumonia and severe respiratory issues, including acute respiratory distress. Encephalitis and seizures arise in severe cases, leading to coma in 24 - 48 h. |

**Supplementary Table 3.** Range of vaccines clinically approved by WHO*

| **Vaccine category** | **Developer/** | **No. of countries got approval** | **No. of clinical trials / countries** | **Characteristic nature of vaccine** | **Country and the date of WHO sanction** |
| --- | --- | --- | --- | --- | --- |
| COVID-19 vaccine (SARS-CoV-2 rS recombinant, adjuvanted) | NUVAXOVID™  Novavax  NVX-CoV2373 | 30 | 11/7 | Protein Sub unit | Czech Rep. /Netherlands  20^th^ December 2021 |
| COVID-19 vaccine (SARS-CoV-2rS Protein Nanoparticle [Recombinant]) | Serum Institute of India, COVOVAX (Novavax formulation) | 3 | 2/1 | Protein Sub unit | India  17^th^ December 2021 |
| mRNA Vaccine (nucleoside modified) | Moderna Biotech  mRNA-1273 | 83 | **35/9** | RNA | Spain  30^th^ April 2021 |
| BioNtech Tozinameran – COVID-19 mRNA vaccine (nucleoside modified) – COMIRNATY | [Pfizer/BioNTech BNT162b2](https://covid19.trackvaccines.org/vaccines/6/) | 130 | **49/23** | RNA | Germany/Netherlands  31^st^ December 2020 |
| Janssen–Cilag International NV (Belgium) COVID-19 Vaccine (Ad26.COV2-S [recombinant] | [Janssen (Johnson & Johnson) Ad26.COV2.S](https://covid19.trackvaccines.org/vaccines/1/) | 99 | **16/18** | Non Replicating Viral vector | Belgium/Netherlands  12^th^ March 2021 |
| AstraZeneca/SKBio - COVID-19 Vaccine (ChAdOx1-S [recombinant]) | [Oxford/AstraZeneca AZD1222](https://covid19.trackvaccines.org/vaccines/4/)Vaxzevria | 134 | **52/23** | Non Replicating Viral vector | Republic of Korea  15^th^ February 2021 |
| Serum Institute of India Pvt Ltd - COVID-19 Vaccine (ChAdOx1-S [recombinant]) - COVISHIELD™ | [Serum Institute of India, Covishield (Oxford/AstraZeneca formulation)](https://covid19.trackvaccines.org/vaccines/48/) | 47 | **2/1** | Non Replicating Viral vector | India  15^th^ February 2021 |
| COVID-19 vaccine (Whole Virion Inactivated Corona Virus vaccine) | [Bharat Biotech Covaxin](https://covid19.trackvaccines.org/vaccines/9/) | 12 | **7/1** | Inactivated | India  03^rd^ November 2021 |
| COVID-19 Vaccine BIBP/Sinopharm | [Sinopharm (Beijing) BBIBP-CorV (Vero Cells)](https://covid19.trackvaccines.org/vaccines/5/) | 80 | **19/10** | Inactivated | China  07^th^ May 2021 |
| Sinovac COVID-19 vaccine (Vero Cell (Inactivated)) – CoronaVac. | [Sinovac CoronaVac](https://covid19.trackvaccines.org/vaccines/7/) | 48 | **26/8** | Inactivated | China, 01^st^ June 2021 |

*Please refer: <https://covid19.trackvaccines.org/agency/who/>

**Supplementary Table 4**. Range of nano-enabled and next-generation vaccines with significant characteristics

| **Vaccine Category** | **Vaccine Developer** | **Clinical phase and status** | **Phase III Efficacy^a^** |
| --- | --- | --- | --- |
| mRNA vaccines | Pﬁzer/BioNTech (BNT162b2) Multinational | Phase III, FDA, EUA granted^b^ | 95% |
| mRNA vaccines | Moderna (mRNA-1273) USA | Phase III, FDA, EUA granted^b^ | 94.5% |
| DNA vaccines | Inovio Pharmaceuticals DNA plasmid (INO-4800) | Phase II/III |  |
|  | Zydus Cadila DNA plasmid (ZyCoVD) India Entos proteolipid vesicle | Phase III |  |
| Protein subunit vaccines | Novavax (Nvx-CoV2373) USA | Phase III | 89% |
| Peptide subunit vaccines | Vektor State Research Center of Virology and Biotechnology (EpiVacCorona) Russia | Phase I/II approved |  |
| Virus-like particles | Medicago; GSK: Dynavax plant based vaccine DNA delivery | Phase II/III |  |

^a^Eﬃcacy data has given by those vaccine developers finished the phase III clinical trials, through heterogeneous criteria. ^b^Approval is allowed in some countries (not given here). Vaccines receiving emergency use authorization (EUA) in the USA that labeled distinctly for two mRNA-based vaccines.

**References**

1. Lin SP, Calcagno A, Letendre SL, Qing M. Clinical Treatment Options and Randomized Clinical Trials for Neurocognitive Complications of HIV Infection: Combination Antiretroviral Therapy, Central Nervous System Penetration Effectiveness, and Adjuvants. Curr Top Behav Neurosci. 2021;1-29. https://doi.org/10.1007/7854_2020_186.
2. Chu C, Selwyn PA. Complications of HIV Infection: A Systems-Based Approach. Am Fam Physician. 2011;83:395-406. PMID: 21322514.
3. Jens B. The history of hepatitis C virus (HCV): Basic research reveals unique features in phylogeny, evolution and the viral life cycle with new perspectives for epidemic control. J Hepatol. 2016;65:S2–S21. https://doi.org/10.1016/j.jhep.2016.07.035.
4. Florian K, Gavin JDS, Ron AMF, Malik P, Katherine K, Peter CD, et al. Influenza. Nat Rev Dis Primers. 2018;4: <https://doi.org/10.1038/s41572-018-0002-y>.
5. Smith W, Andrewes CH, Laidlaw PP. A virus obtained from influenza patients. Lancet. 1933;222:66-68. <https://doi.org/10.1016/S0140-6736(00)78541-2>.
6. Lea S. Eiland Respiratory Syncytial Virus: Diagnosis, Treatment and Prevention. J Pediatr Pharmacol Ther, 2009;14:75-85. <https://doi.org/10.5863/1551-6776-14.2.75>.
7. Liang TJ. Hepatitis B: The Virus and Disease. Hepatol. 2009;49:S13–S21. <https://doi.org/10.1002/hep.22881>.
8. Liqin C, Yan W, Juan D. Human Papillomavirus Vaccines: An Updated Review. Vaccines. 2020;8:391. <https://doi.org/10.3390/vaccines8030391>.
9. Jürgen G, Joel T, Afsar R, Cecilia SN, Katja V. A Review of the Potential Role of Human Cytomegalovirus (HCMV) Infections in Breast Cancer Carcinogenesis and Abnormal Immunity. Cancers. 2019;11:1842. <https://doi.org/10.3390/cancers11121842>.
10. Pamela C, Joseph VB, Andrew CK, Sheila FF. Herpes Simplex. Pediatr Rev. 2009;30:119-130. <https://doi.org/10.1542/pir.30-4-119>.
11. Arvin AM. Varicella-zoster virus. Clin. Microbiol. Rev. 1996;361-381.

<https://doi.org/10.1128/CMR.9.3.361>.
